# Japanese Classification of Esophageal Cancer, 11th Edition: part II and III

**DOI:** 10.1007/s10388-016-0556-2

**Published:** 2016-11-10

**Authors:** Japan Esophageal Society

**Affiliations:** Hirose-Building 4F, Taihei 2-3-13, Sumida-ku, Tokyo, 130-0012 Japan

**Contents**

Part II Explanations

2.1.2. Tumor location
2.1.3. Macroscopic tumor type
2.1.4. Depth of tumor invasion (T)

2.2. Metastatic lesions from esophageal cancer

2.2.1. Lymph node metastasis
  2.2.1.1. Naming and numbers of lymph node stations
  2.2.1.2. Lymph node groups

2.4. Multiple primary cancers

3.4.3. Lymph node dissection
  3.4.3.1. Types of lymph node dissection

3.7. Curativity (Cur)

4.2.1. Histological classification
  4.2.1.1. Benign epithelial neoplasms
  4.2.1.2. Intraepithelial neoplasias
  4.2.1.3. Malignant epithelial neoplasms
  4.2.1.4. Non-epithelial tumors
  4.2.1.5. Lymphoid tumors
  4.2.1.6. Other malignant tumors
4.2.2. Depth of tumor invasion (pT)
4.2.9. Pathological criteria for the effects of radiation and/or chemotherapy

6. Barrett esophagus, and adenocarcinoma in Barrett esophagus

6.1.3 Barrett esophagus
  6.1.3.1 Macroscopic findings
  6.1.3.2 Pathological findings
6.1.4 Adenocarcinoma in Barrett esophagus

Figures of Pathological Findings

Part III Response evaluation criteria in radiotherapy and chemotherapy for esophageal cancer

Introduction

1. Subjects
  1.1. Classification of tumor lesions
    1.1.1. Measurable lesions
    1.1.2. Non-measurable lesions
    1.1.3. Target lesions
    1.1.4. Non-target lesions
2. Methods for response evaluation
3. Response evaluation criteria for target lesions
  3.1. Complete response (CR)
  3.2. Partial response (PR)
  3.3. Progressive disease (PD)
  3.4. Stable disease (SD)
4. Response evaluation criteria for non-target lesion
  4.1. Complete response (CR)
  4.2. Incomplete response/stable disease (IR/SD)
  4.3. Progressive disease (PD)
5. Response evaluation criteria for primary lesion using endoscopy
  5.1. Complete response of primary lesion (primary lesion CR)
  5.2. Incomplete response/stable disease of primary lesion (primary lesion IR/SD)
  5.3. Progressive disease of primary lesion (primary lesion PD)
6. Overall Response
7. Best overall response and confirmation
  7.1. Complete response (CR)
  7.2. Partial response (PR)
  7.3. Stable disease (SD)
  7.4. Progressive disease (PD)

Part II

Explanations

*1.3.1 Principles of description and methods of abbreviation*

General findings are often described as findings. In such cases, the “f” representing general findings may be omitted, and a description such as “MtLt, 10 cm, Type 2, T2, N0, M0, Stage II” is acceptable. When clinical findings need to be distinguished from pathological findings, descriptions such as “Although surgery was performed based on a preoperative finding of cT2,cN0, the pathological finding was pT3,pN1” are acceptable.

2.1 Description of primary tumor

2.1.1. The number and size of the lesions needs to be described; if multiple peripheral tumors are present, the location, size, tumor type, and depth of invasion should be described for each lesion. In cases with multiple lesions, the major lesion is the deepest lesion (or the lesion with the largest dimension if the depth of tumor invasion is equal).

In cases where evaluation using X-ray or endoscopy is only possible in one direction, the largest dimension measured should be described as the clinical finding.

*2.1.2. Tumor location*

[Diagnostic criteria for cancer located at the esophagogastric junction]

The esophagogastric junction (EGJ) should be defined systematically in accordance with the criteria listed below. It is important that a diagnosis be made before the initiation of treatment. Endoscopic findings should be prioritized over findings obtained using other diagnostic modalities. Cancer at the EGJ means that the tumor center is located between 2 cm proximal to and distal from the EGJ. When the tumor size or tumor invasion makes it difficult to define the EGJ, the location of the EGJ should be comprehensively identified based on general findings. In such instances, the tumor location should be described with an acknowledgement that the diagnosis was not based on strict diagnostic criteria for EGJ cancer.

1. Endoscopic findings

The EGJ is defined as the lower margin of palisading small vessels (Fig. 2-1a–c).

**Fig. 2-1:**
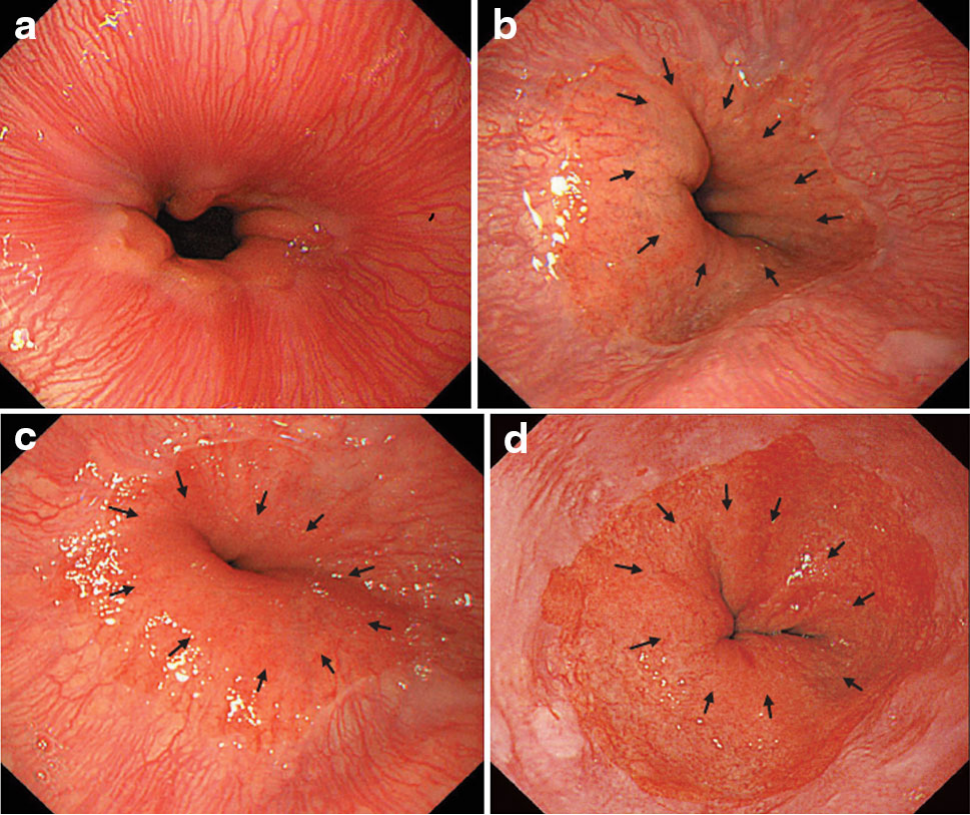
**a** The lower margin of the palisading small vessels and the oral margin of the longitudinal folds of the greater curvature of the stomach are both clearly visible and coincident at the same level. In such cases, this site of coincidence is defined as the EGJ and is nearly identical to the SCJ. **b** The lower margin of the palisading small vessels and the oral margin of the longitudinal folds of the greater curvature of the stomach are both clearly visible and coincident at the same level. In such cases, this site of coincidence is defined as the EGJ (*black arrows*). The gap between the SCJ and the EGJ is diagnosed as Barrett esophagus. **c** The palisading small vessels are visible, but the longitudinal folds are unclear. The* lower* margin of the palisading small vessels is defined as the EGJ (*black arrows*). The gap between the SCJ and the EGJ is diagnosed as Barrett esophagus. **d** The longitudinal folds are visible, but the *lower* margin of the palisading vessels is unclear. The *upper* oral margin of the longitudinal fold is defined as the EGJ (*black arrows*). The gap between the SCJ and the EGJ is diagnosed as Barrett esophagus

If the palisading small vessels cannot be clearly identified, the oral margin of the longitudinal folds of the greater curvature of the stomach can be defined as the EGJ (Fig. 2-1d).

■ Commentary: Esophageal sphincters are located at both the proximal and distal ends of the esophagus and are referred to as the upper esophageal sphincter (UES) and the lower esophageal sphincter (LES), respectively. The propria mucosa layer of these two loci contains palisading small vessels that penetrate the muscularis mucosa layer after branching from submucosal vessels. Since the sphincter muscles are considered to be a feature of the esophagus, the esophagus is defined as the tract between the upper margin of the UES and the lower margin of the LES. Because the lower margin of the LES is defined as the EGJ, the level of the EGJ can be defined as the level of the lower margin of the lower palisading vessels. With this interpretation in mind, the majority of Japanese experts define the EGJ endoscopically as the lower margin of the lower palisading vessels. The endoscopic observation of palisading small vessels should be performed under a condition in which the lower esophagus is adequately stretched after suctioning the air from inside the stomach and the examinee has inhaled deeply.

The palisading small vessels may be difficult to distinguish in endoscopic examinations of cases with gastroesophageal reflux disease or long segment Barrett esophagus (LSBE). Also, constant esophageal stretching cannot be attained by a deep inhalation if the patient has been sedated. The majority of Western experts have long used the criterion that the EGJ be defined as the upper end of the longitudinal folds of the stomach. The EGJ is defined as the upper end of the longitudinal folds of the stomach in the Prague C&M Classification, developed by the International Working Group for the Classification of Oesophagitis (IWGCO) in 2006. Endoscopic observation of the upper end of the longitudinal folds should be performed with optimal decompression of the stomach through suction; however, “optimal decompression” has not been strictly defined. The oral margin of the longitudinal folds of the stomach cannot be reliably defined if the amount of air inside the stomach varies. Thus, the diagnostic concordance rate is not very high for the detection of short segment Barrett esophagus (SSBE) extending for lengths smaller than 1 cm. Moreover, the gastric folds tend to be smaller in the presence of atrophic gastritis, which is more common in Japan, and the upper end of the longitudinal folds cannot be reliably observed if the amount of gas inside the stomach varies. With these points in mind, the upper end of the longitudinal folds can be difficult to define in some cases.

In making a diagnosis, the EGJ should be defined with a comprehensive interpretation of both the palisading small vessels and the longitudinal folds. In Japan, LSBE is less common and atrophic gastritis is more common. Therefore, in the Japanese Classification of Esophageal Cancer, the lower margin of the palisading vessels is primarily used to define the EGJ. The upper end of the longitudinal folds is used as a secondary criterion in cases where the palisading vessels are difficult to distinguish.

2. X-ray: Upper gastrointestinal series

- The EGJ is defined as the narrowest locus of the lower esophagus (Fig. 2-2).

**Fig. 2-2:**
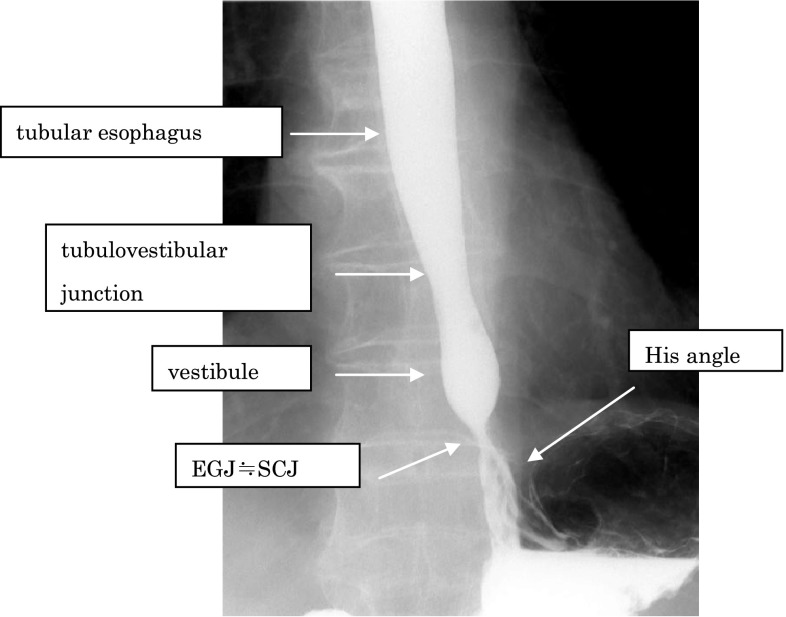
Barium contrast image of a normal EGJ
- In cases with a sliding hiatal hernia, the EGJ should be identified as the oral margin of the longitudinal folds (Fig. 2-3).

**Fig. 2-3:**
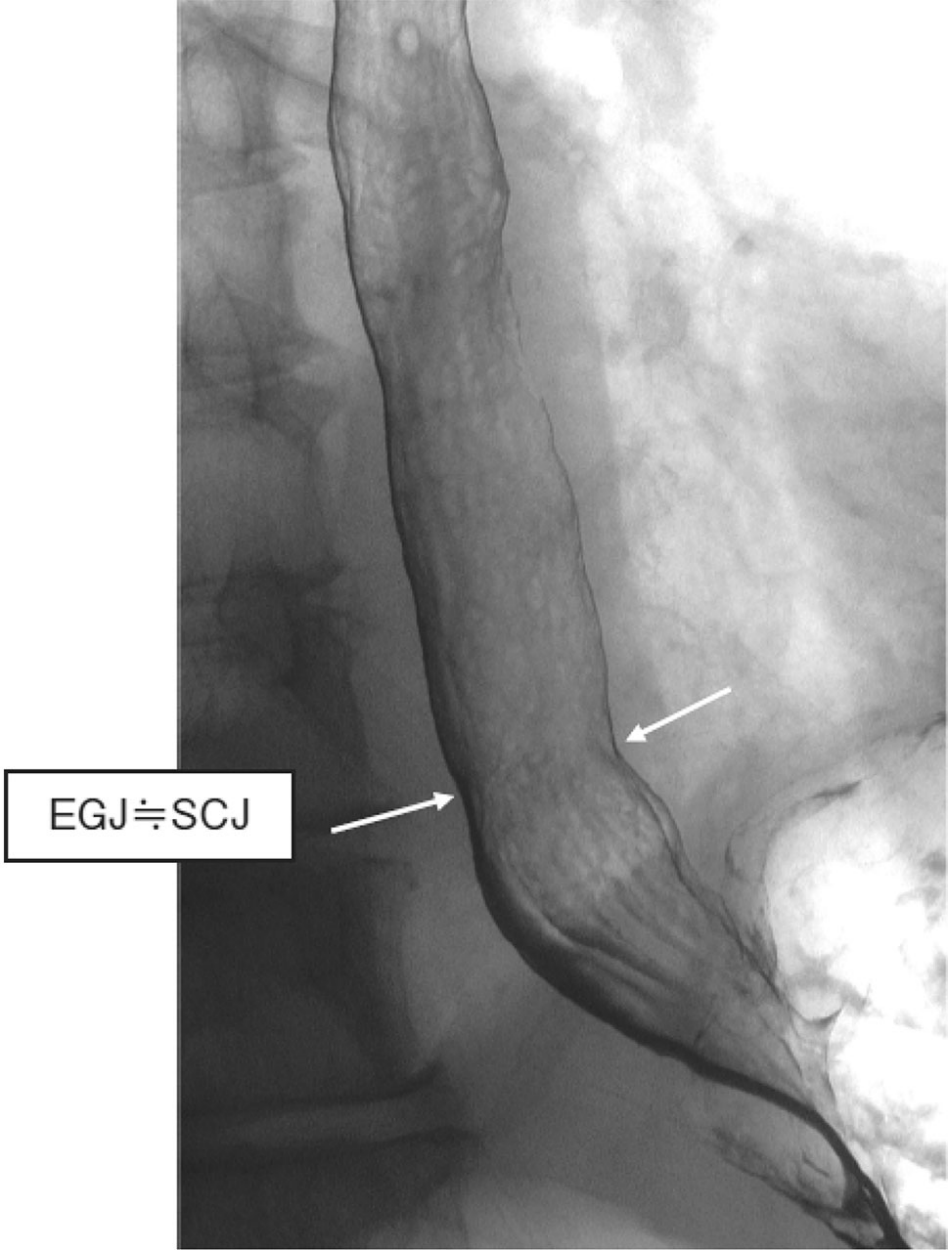
Barium contrast image of the EGJ in a subject with a hiatal hernia
- In the presence of Barrett esophagus, the SCJ is located on the oral side of the EGJ, and Barrett mucosa appears as mucosa containing reticular structures in a double-contrast study (Fig. 2-4). The EGJ is identified as the oral margin of the longitudinal folds.

**Fig. 2-4:**
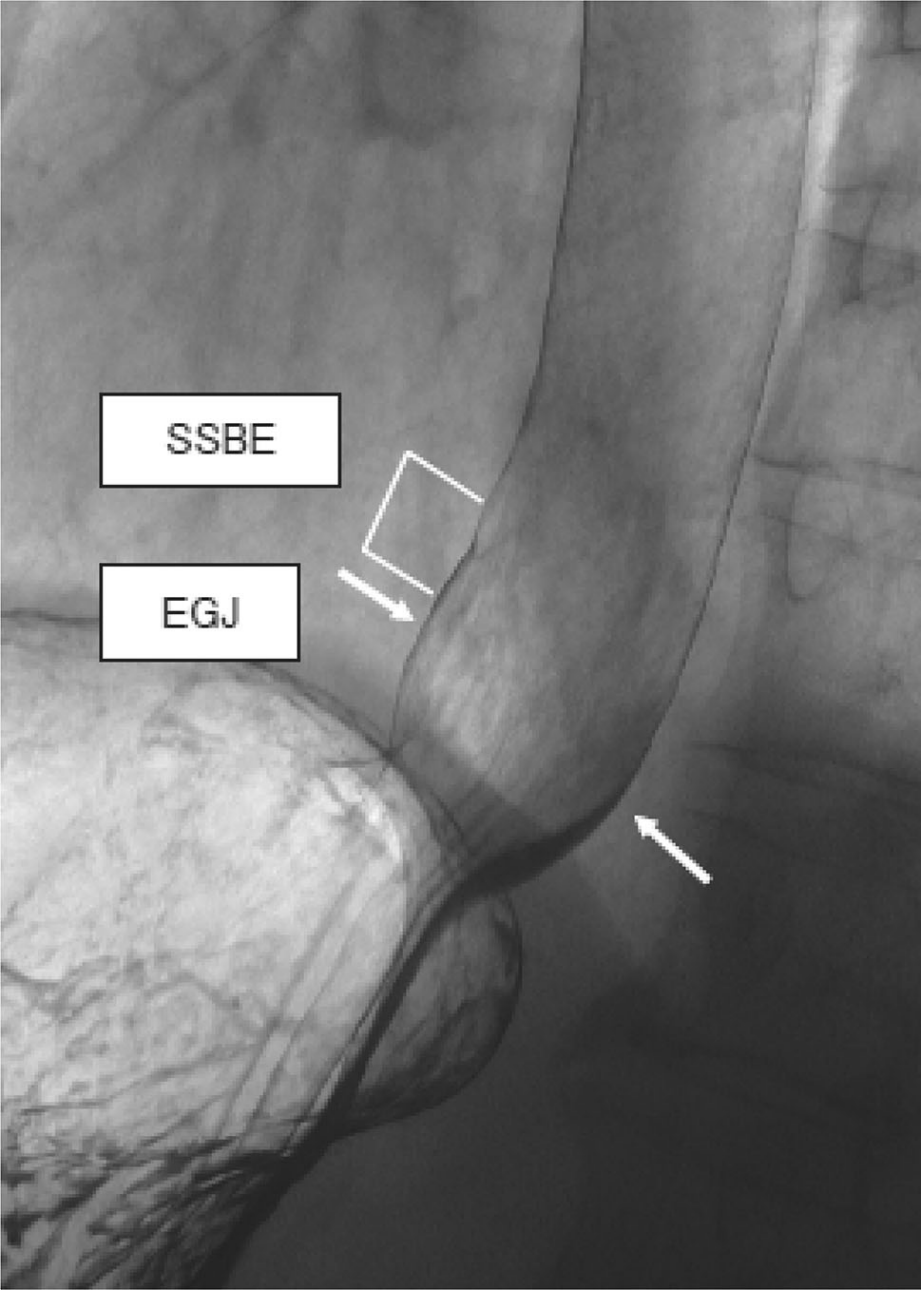
Barium contrast image of the EGJ in a subject with Barrett mucosa

■ Commentary: In a barium contrast series, the esophagus can be divided into two segments of tubular and vestibule (or saccular) esophagus with a junction called the tubulovestibular junction, where the lumen of the esophagus gradually narrows. The Angle of His designates the angle formed by the esophagus and the fornix of the stomach. In healthy subjects, the Angle of His is acute, and the EGJ is identical to the narrowest part of the lower esophageal lumen. A muscular fiber lies between the left side of the esophagus and the entry of the stomach, resulting in the acuteness of the Angle of His. This fiber is located inside the propria muscle of the stomach and is called the “sling fiber”. In healthy subjects, the level of the EGJ is nearly identical to the level of the SCJ.

Commentary: In cases with a hiatal hernia, a circular “neck” of the esophageal lumen is observed close to the hiatus or at the lower level of the pleural cavity during a barium contrast study, and this neck is referred to as a mucosal ring (B ring) or “Z-line”. Although this neck is referred to as a “ring”, the ring is not formed by a circular fiber of the muscular propria. Instead, the “ring” is presumably formed by the sling fiber and is prominent on the left side, or the fornix side, of the esophagus. In cases with a hiatal hernia, the EGJ is usually imaged as the oral margin of the longitudinal folds of the stomach.

Commentary: The “circular neck” of the esophagus accompanying a hiatal hernia is often obscured in the presence of Barrett mucosa. Therefore, the EGJ is usually defined as “the oral margin of the longitudinal folds of the stomach” in subjects with Barrett mucosa.

3. Pathological study

Macroscopic definition: The EGJ should be defined macroscopically as the point at which the luminal caliber changes in the area where the tubular esophagus is connected to the vestibule lumen of the stomach.

Microscopic definition: For a mucosal layer with intact structures, the EGJ should be defined as follows:

1. Non-Barrett esophagus: The EGJ is defined as the squamocolumnar junction.

2. Barrett esophagus: Histological structures such as proper esophageal glands and their ducts, a double-layer muscularis mucosa, or palisading small vessels should be included in the microscopic definition of the EGJ.

For a non-intact mucosal layer, the EGJ should be defined based on the macroscopic findings of the surgical specimen, and the EGJ should be presumed based on the presence of histological structures associated with the esophagus or stomach.

■ Commentary: To define the EGJ, the surgical specimen must be fully extended and optimally fixed. The histological structures must be examined using a full section of the whole specimen. If the palisading small vessels are visible in a fresh specimen, the anal margin of the vessels should be marked before the histological inspection. The EGJ is defined as “the borderline between the muscular structures of the esophagus and the stomach”; however, histological discrimination of the muscular structures of each area is difficult. The EGJ is also often histologically unclear. Therefore, the histological definition of the EGJ depends on the macroscopic findings or the histological features of the mucosal or submucosal layer.

The definition of the histological EGJ should be given individually for non-Barrett esophagus and Barrett esophagus cases.

① Non-Barrett esophagus
  The SCJ is identical to the level of the lumen where the esophagus connects to the stomach and the macroscopic luminal caliber changes; this location is approximately identical to the EGJ. The SCJ can be visualized using Lugol staining, appearing as a linear or slightly curved borderline between squamous and columnar epithelium. In cases with a hiatal hernia, the luminal caliber change tends to be obscured, but the EGJ can often be defined after a closer inspection.
② Barrett esophagus
  The SCJ is not identical to the EGJ.
  - Non-circular Barrett esophagus
    The line of the SCJ adjacent to the non-Barrett epithelium is identical to the EGJ.
  - Circular Barrett esophagus
    The EGJ can be defined as the most anal level of the lumen where any of the following histological features are observed:
  Presence of proper esophageal glands or esophageal gland tubules beneath the columnar epithelium
  Presence of squamous islands in the columnar epithelium
  Presence of a double-layer muscularis mucosae beneath the columnar epithelium
  Palisading vessels

The level of the luminal caliber change is included in the definition of the EGJ.

[Cancer at the EGJ]

Since the pattern of lymph node metastasis for EGJ cancer differs from that of carcinomas of the lower esophagus or the upper stomach, special attention should be paid to the surgical procedure. Consequently, EGJ cancer is dealt with separately in the Japanese Classification. Previously, Nishi’s classification was used for the definition of EGJ cancer. According to this classification, EGJ cancer includes lesions with a tumor center located between 2 cm proximal to and distal from the EGJ, irrespective of histology (Fig. 2-5). Accordingly, cancer of the abdominal esophagus is included in EGJ cancer.

**Fig. 2-5:**
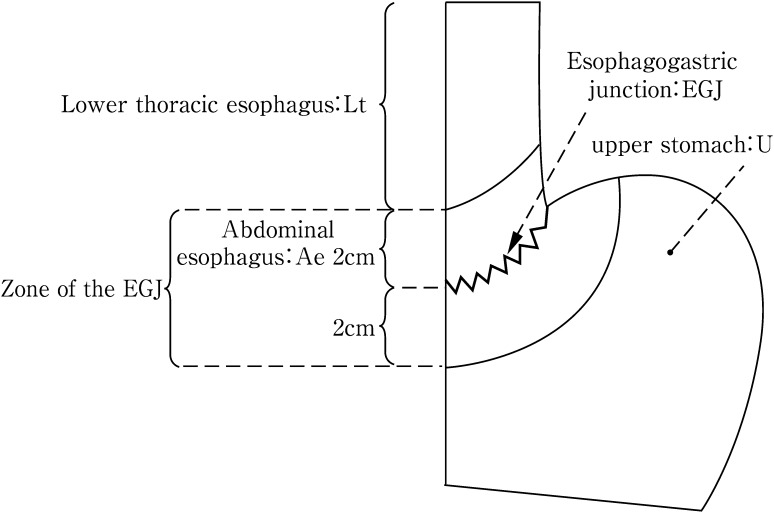
Definition and description of esophagogastric junction according to Nishi’s classification

In Western countries, Siewert’s classification is commonly used. According to Siewert’s classification, cancer at the EGJ is defined as adenocarcinoma with a tumor center located within 1 cm distal from and 2 cm proximal to the EGJ (Fig. 2-6). EGJ cancer corresponds to Siewert Type II-True cardia cancer.

**Fig. 2-6:**
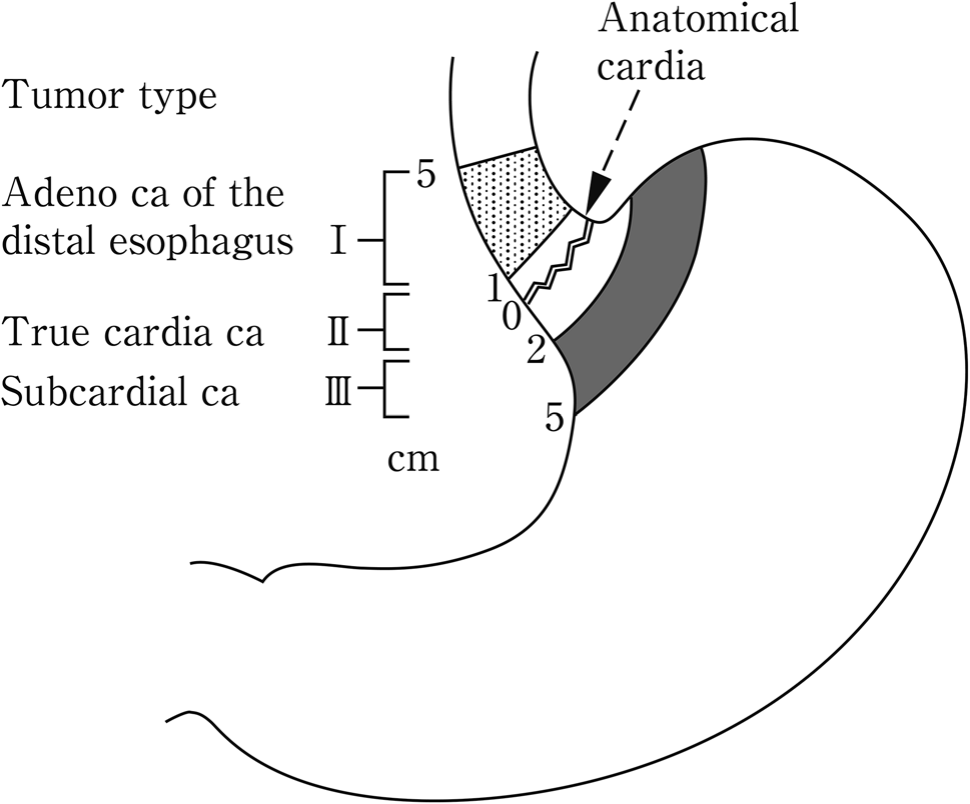
Definition and description of adenocarcinoma at the esophagogastric junction according to Siewert’s classification

Adenocarcinoma in Barrett esophagus cannot be excluded from a diagnosis of EGJ cancer located in the abdominal esophagus.

- Note 1: In cancers located at the EGJ, the oral and anal portions of the EGJ are described as “E” and “G”, respectively. As shown in Fig. 2-3, the terms “E, EG, E=G, GE, and G” can be used depending on the tumor location.
- Note 2: In cases with adenocarcinoma in Barrett esophagus, the disease should be described.
- Note 3: Siewert’s classification Types I, II and III (Fig. 2-6) should also be described for adenocarcinoma located in the lower esophagus or at the EGJ (Fig. 2-7).

**Fig. 2-7:**
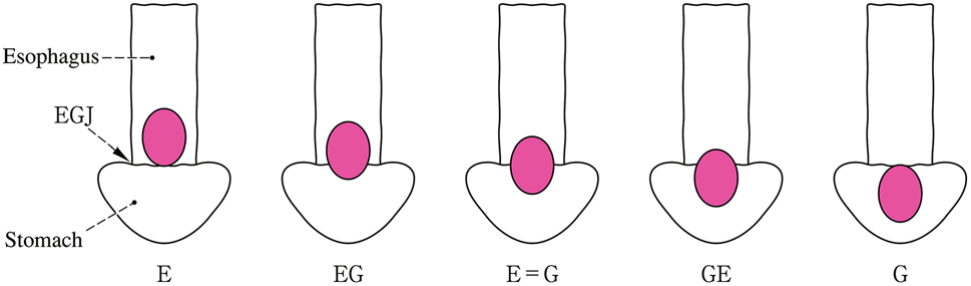
Subclassification and methods of description of cancer at the esophagogastric junction

[References]

(1) Nishi M, Kajisa T, Akune T, et al. Cardia cancer—proposal of cancer in the esophagogastric junction (in Japanese). Geka Shinryo (Surgical Diagnosis and Treatment) 1973; 15: 1328–1338.

(2) Siewert JR, Stein HJ. Carcinoma of the cardia: carcinoma of the gastroesophageal junction—classification, pathology and extent of resection. Dis Esophagus 1996; 9: 173–182.

(3) Japanese Gastric Cancer Association. Japanese Classification of Gastric Carcinoma (in Japanese). 13th ed. Kanehara Shuppan, Tokyo, 1999; 39.

2.1.3. Macroscopic tumor type

2.1.3.2. Macroscopic classification (Figs. 2-8, 2-9, 2-10)

**Fig. 2-8:**
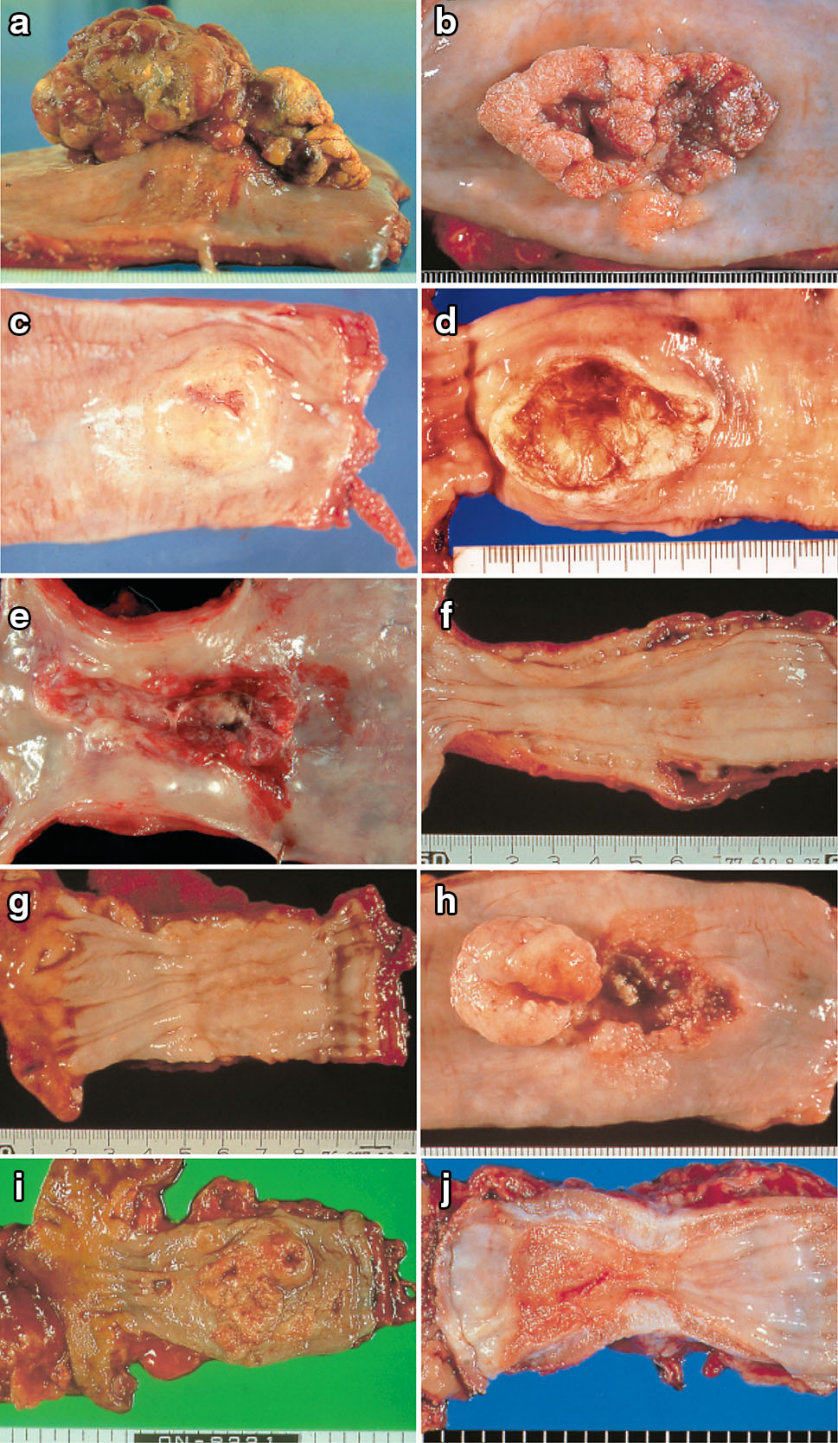
**a** Type 1: a pedunculated and tall polypoid lesion. This was judged to be advanced cancer based on its size, mobility (or cut cross section). **b** Type 1: this protruding type lesion with a clearly demarcated border has lobules or a papillary appearance on its surface. **c** Type 1: most of the surface of the protrusion is covered by non-cancerous epithelium. This was judged to be advanced cancer based on its size and immobility. **d** Type 2: this lesion is a deep ulcer with a well-demarcated surrounding ridge. Macroscopic findings: advanced type (Types 1–5). **e** Type 3: this lesion is a deep ulcer surrounded by a poorly demarcated ridge. The lesion extended circumferentially causing luminal stenosis. **f** Type 4: this diffusely invasive lesion with no clear margin makes the esophageal wall *thick* and *hard*, and causes luminal stenosis. No distinct ulcer can be seen. **g** Type 4: the *thickening* of the esophageal wall and the edematous changes of the mucosa suggest diffuse intramural extension of the lesion, but there is no finding of hardening or stenosis, and no finding of ulcer formation. **h** Combined type: this cancer showed mixed morphology of advanced Type 1 and Type 2 (0-IIc Type extension can be seen in part). **i** Type 5a: the macroscopic appearance is extremely complex with Type 1, and Type 2 and others, and it is difficult to categorize. **j** Type 5b: this macroscopic tumor (Type 5b) cannot be categorized because of preoperative chemoradiotherapy. **i** Type 5a: the macroscopic appearance is extremely complex with Type 1, and Type 2 and others, and it is difficult to categorize. **j** Type 5b: This macroscopic tumor (Type 5b) cannot be categorized because of preoperative chemoradiotherapy

**Fig. 2-9:**
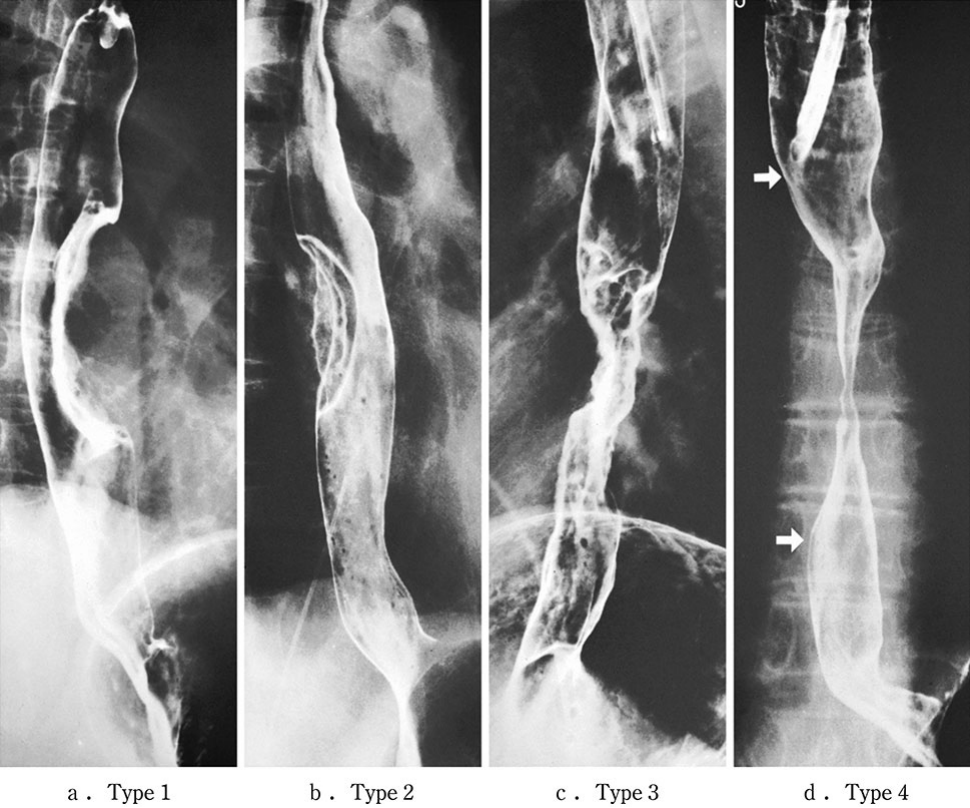
Roentgenological findings: advanced type

**Fig. 2-10:**
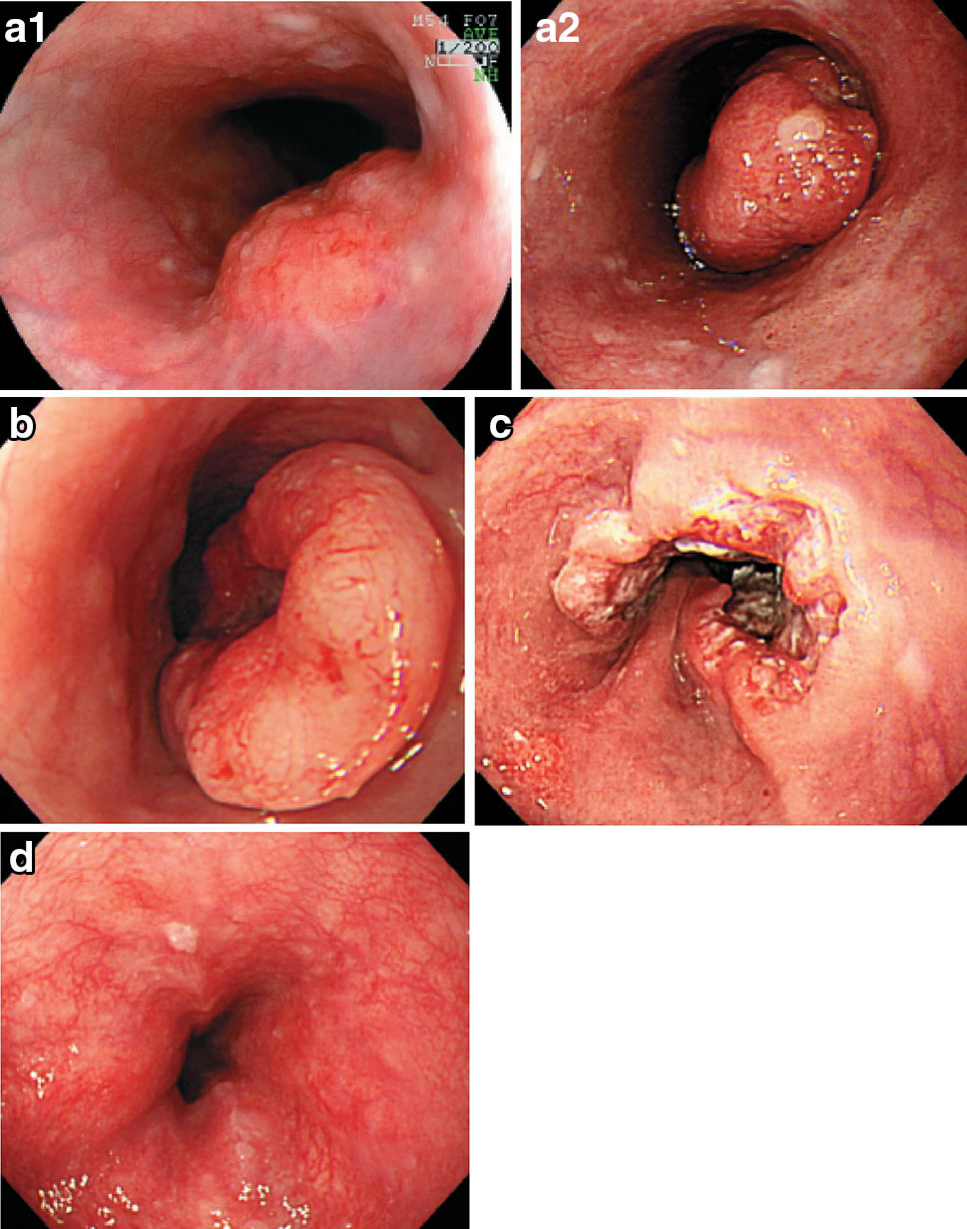
Endoscopic findings: advanced type. **a1** Type 1, protruding type (pT2): a tall lesion with a broad base. **a2** Type 1, protruding type (pT2): a tall lesion with a narrow base. **b** Type 2, ulcerative and localized type (pT3): a deep ulcerative lesion surrounded by a well-demarcated ridge. **c** Type 3, ulcerative and infiltrative type (pT3): a deep ulcerative lesion surrounded by an ill-demarcated ridge. **d** Type 4, diffusely infiltrative type (pT3): ill-defined thickening and hardening of the esophageal wall accompanied by luminal stenosis is observed. There is no remarkable ulcer formation

Type 0: Superficial type: Tumor invasion is limited to the submucosa.
Type 1: Protruding type: localized protruding lesion.
  The definitely protruding lesion which commonly has an erosive surface. However, the lesion is occasionally covered by intact squamous epithelium continuing from surroundings.

Type 2 Ulcerative and localized type: The ulcerative lesion has a well-demarcated surrounding ridge.
Type 3 Ulcerative and infiltrative type: The ulcerative lesion has an ill-demarcated surrounding ridge circumferentially or semi-circumferentially.
Type 4 Diffusely infiltrative type: Lesion with wide intra-mural invasion, and generally without conspicuous ulcer or protrusion. Even if the lesion has ulcerative and/or protruding components, it is defined as type 4.
Type 5 Unclassifiable type: The lesion with a complicated macroscopic appearance which is unclassifiable to any of macroscopic tumor types 0–4.
  5a The unclassifiable lesion without previous treatment.
  5b The lesion unclassifiable because of a changed appearance with previous treatment.

The lesion after treatment(s) should be classified into macroscopic tumor types 0–4 if possible. The lesion with previous treatment(s) should be distinguished with the sign of treatment.

2.1.3.3. Subclassification of superficial type (Figs. 2-11, 2-12, 2-13)

**Fig. 2-11:**
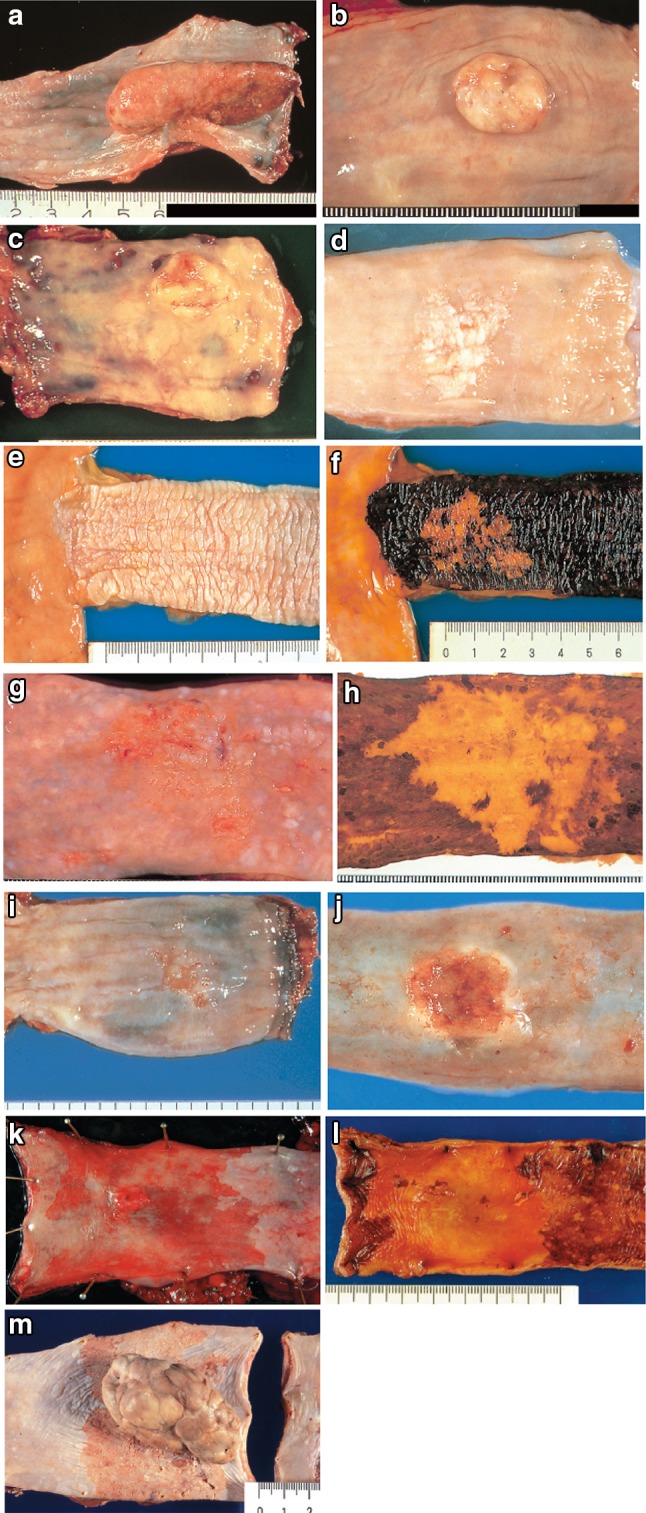
Macroscopic findings: superficial type (0 type). **a** Type 0-Ip (superficial and protruding type, pedunculated): The tumor is well demarcated and has a narrow base. **b** Type 0-Ip (superficial and protruding type, pedunculated): The well-demarcated, protruding tumor has an irregular and nodular surface. **c** Type 0-Is (superficial and protruding type, sessile): the surface of this ill-demarcated tumor is mostly covered by the normal epithelium. **d** Type 0-IIa (slightly elevated type): the tumor is only slightly elevated from the mucosa. Its *color* is generally *white*. **e** Type 0-IIb (flat type): there are only minute irregularities and no macroscopic abnormal features. **f** Type 0-IIb (flat type) (iodine-stained view of **e**) the superficial tumor can now be seen unstained by iodine. **g** Type IIc (slightly depressed type): the superficial depressed lesion has no clear margin and a finely granular surface. **h** (iodine-stained view of **g**): the superficial tumor is unstained by iodine. **i** Type 0-IIc (slightly depressed type): the superficial depressed lesion has an irregular margin. **j** Type 0-III (superficial and depressed type): the deeply depressed lesion with a slightly elevated margin suggests invasion beyond the muscularis mucosae. **k** Type 0-IIc+”0-IIa” (superficial spreading type): the widespread slightly depressed *red* lesion (0-IIc) has a slightly elevated lesion (0-IIa) in its center, suggesting invasion into the submucosal layer. The lesion, more than 5 cm in length, is defined as the superficial spreading type. **l** Type 0-IIc+”0-IIa” (superficial spreading type) (iodine-stained view of **k**): the reddish depressed lesion is not stained with iodine solution. **m** Type 0-IIc + “0-Ip”: the well-demarcated protruding tumor with a narrow base (0-Ip) has a slightly depressed lesion (0-IIc) in the surrounding area. This macroscopic appearance is characterized as carcinosarcoma

**Fig. 2-12:**
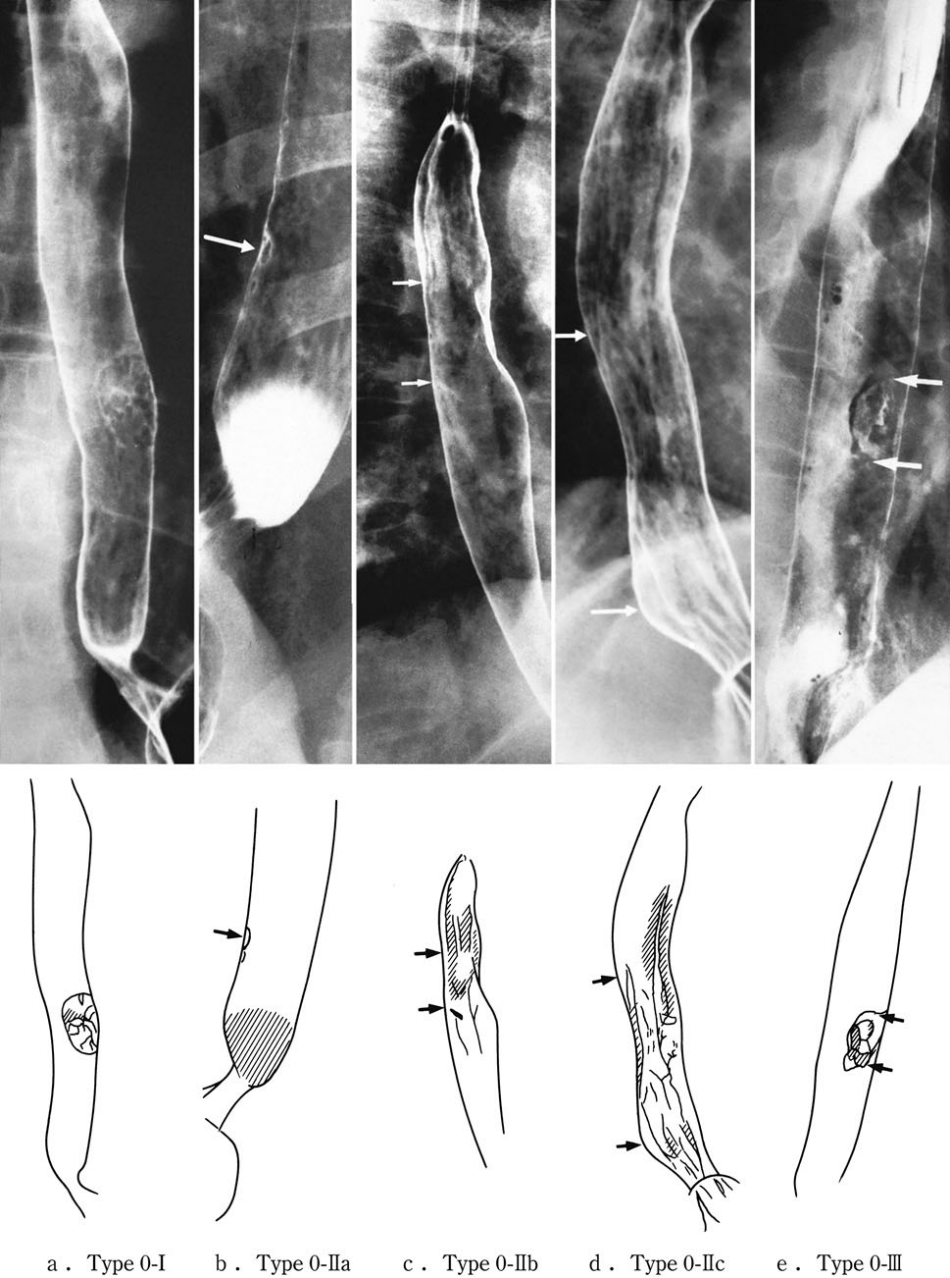
Radiological findings: superficial type

**Fig. 2-13:**
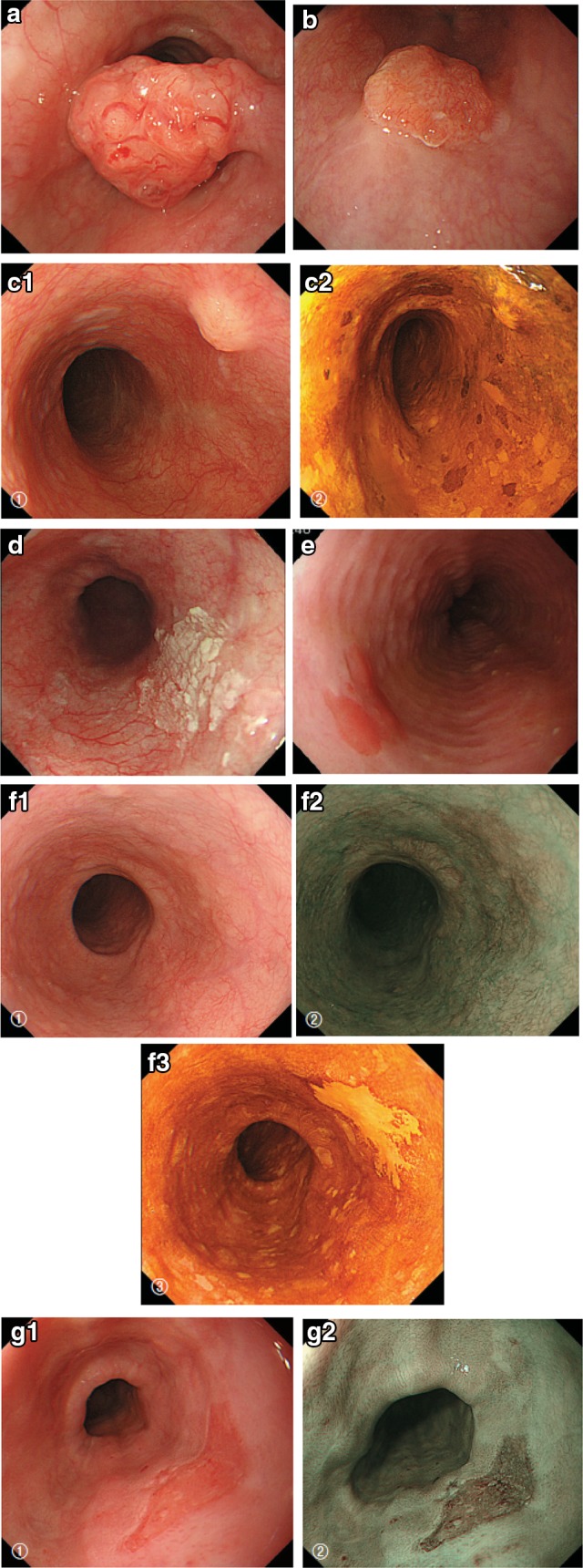

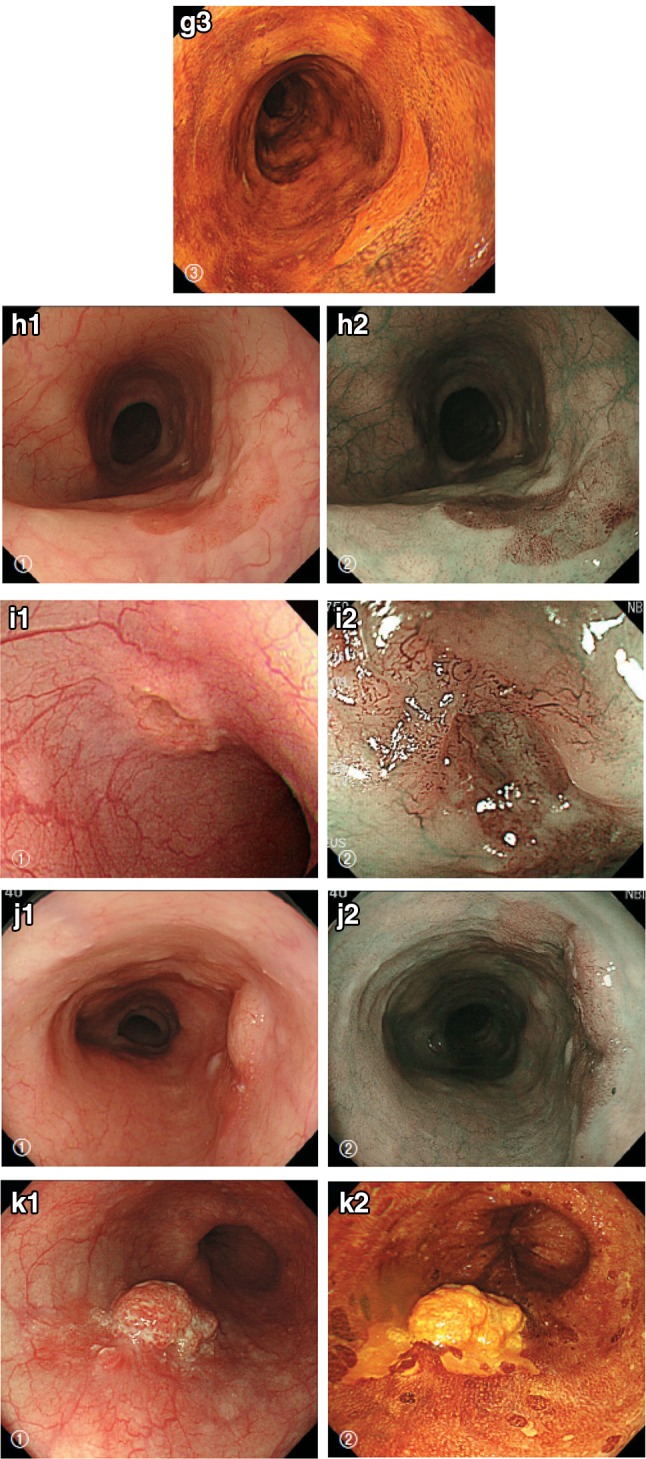
Endoscopic findings: superficial type.** a** Type 0-Ip, superficial and protruding type, pedunculated (cT1b-SM2-3): a well-demarcated protruding and pedunculated tumor shows an irregular and nodular surface.** b** Type 0-Is, superficial and protruding type, sessile (pT1b-SM2): a well-demarcated protruding and sessile tumor.** c **Type 0-Is, superficial and protruding type, sessile (pT1b-SM2): ➀ Conventional endoscopy: an ill-demarcated protruding tumor covered by normal esophageal mucosa suggests a tumor mass in the submucosa. ➁ Iodine staining: the mucosa covering the tumor is stained brown, and an unstained area at the top suggests exposed tumor tissue.** d** Type 0-IIa, slightly elevated type (pT1a-MM): a plaque-like, slightly elevated white tumor. Tumor invasion of the* white *area remained within the lamina propria, while a tiny protrusion at the distal margin of the tumor had invaded the muscularis mucosae.** e** Type 0-IIa, slightly elevated type (pT1a-EP): a slightly elevated tumor with well-demarcated* reddening* (the height of a type 0-IIa lesion is less than 1 mm).** f** Type 0-IIb flat type (pT1a-EP): ➀ Conventional endoscopy: a conventional observation could not detect the lesion. ➁ Narrow band imaging shows a* brownish* area. ➂ Iodine staining: a completely flat lesion was identified as a well-demarcated, unstained area using iodine staining.** g** Type 0-IIc, slightly depressed type (pT1a-LPM). ➀ Conventional endoscopy: an irregularly shaped mucosal* reddening* with a slight depression is visible. ➁ Narrow band imaging: the lesion is also visible as a* brownish* area. ➂ Iodine staining: a well-demarcated, unstained area is visible using iodine staining.** h** Type 0-IIc, slightly depressed type (pT1b-SM1): ➀ Conventional endoscopy: an area of mucosal reddening with a slight depression and marginal elevation is visible. ➁ Narrow band imaging: a* brownish* area suggesting a hypervascular lesion is visible.** i** Type 0-III, superficial and excavated type (cT1b-SM2-3). ➀ Conventional endoscopy, ➁ narrow band imaging (cT1b-SM2-3): a distinctly depressed lesion with a surrounding elevated area is visible using conventional observations, suggesting an ulcer reaching the muscularis mucosa.** j** Combined type 0-IIc + “0-Is” (pT1b-SM2): ➀ Conventional endoscopy: a distinct elevation with a wide base is visible. A slightly depressed lesion was also noted close to the distal margin of the lesion. ➁ Narrow band imaging: a lesion with a well-demarcated margin is visible.** k** Combined type 0-Is + 0-IIc (pT1b-SM2): ➀ Conventional endoscopy: a distinctly protruding lesion with a wide base and irregular, nodular changes is visible.* Reddening* of the esophageal mucosa close to the lesion with an ill-defined margin was suspected. ➁ Iodine staining: The margins of the mucosal changes were identified as well-demarcated, unstained areas

Type 0-I Superficial and protruding type: definitely protruding lesion diagnosed as superficial cancer, based on findings of size, height and a relatively narrow basis.
  Type 0-Ip Pedunculated: lesion with a peduncle or semi-peduncle, and generally the height is more conspicuous than the horizontal spread of the base.
  Type 0-Is Sessile (broad based): lesion without a peduncle, and generally the horizontal spread of the base is more conspicuous than the height. Types of 0-Ipl and 0-Isep in the 9th edition of the Japanese Classification are included in this type.
Type 0-II Superficial and flat type: the lesion without definite protrusion or depression.
  Type 0-IIa Slightly elevated type: lesion with a slight elevation (up to about 1 mm in height).
  Type 0-IIb Flat type: the lesion without macroscopic elevation or depression. This cancerous lesion can occasionally be recognized only by iodine staining.
  Type 0-IIc Superficial and depressed type: lesion with a slight depression, commonly accompanied with mucosal reddening. The degree of depression is equivalent to erosion.
Type 0-III Superficial and depressed type: lesion showing more distinct depression than the 0-IIc type, and bottom of the depression appears to extend beyond the muscularis mucosae (Fig. 2-14).

**Fig. 2-14:**
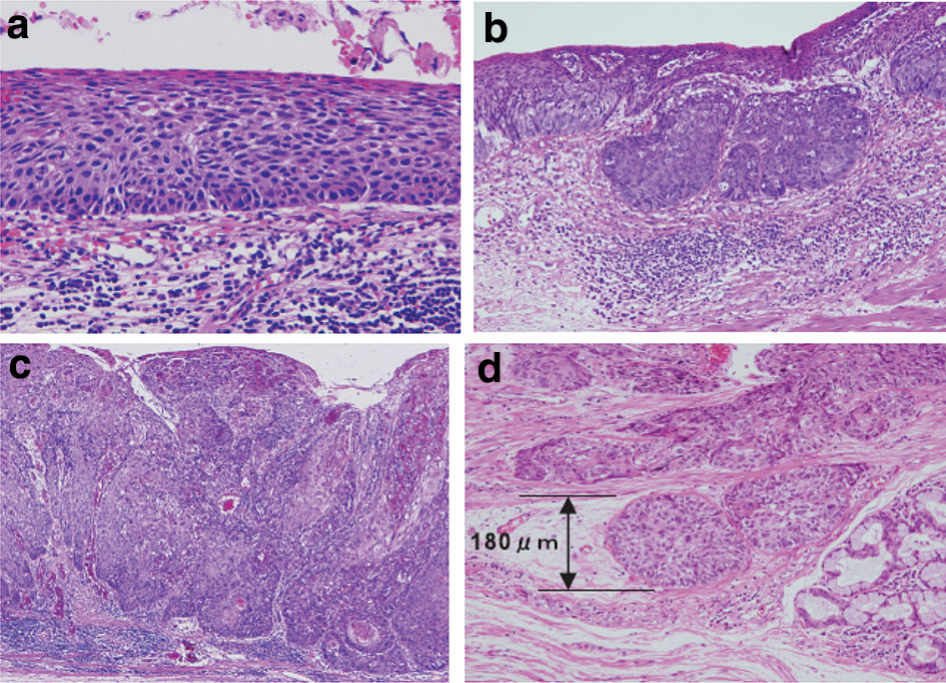
Diagnostic criteria for depth of invasion. **a** T1a-EP (Tis): carcinoma in situ. **b** T1a-LPM: tumor has invaded the lamina propria mucosae. **c** T1a-MM: tumor has invaded the muscularis mucosae. **d** T1b-SM1: tumor invasion is limited to the upper third of the submucosal layer. The *vertical* depth of invasion is 180 μm from the lower edge of the muscularis mucosae. The lesion was diagnosed as pT1b-SM1 in the endoscopically resected specimen

*2.1.4 Depth of tumor invasion (T)*

Depth sub-typing for superficial cancer

In surgically resected tissue specimens, the mucosal layer T1a is divided into three layers (T1a-EP, T1a-LPM, and T1a-MM), and the submucosal layer T1b is also divided into 3 layers (T1b-SM1, T1b-SM2, and T1b-SM3) (Fig. 1-3).

In endoscopically resected specimens, lesions located within 200 μm of the muscularis mucosae are identified as T1b-SM1, and lesions invading beyond this range are identified as T1b-SM2.

2.2 Metastatic lesions from esophageal cancer

*2.2.1 Lymph node metastasis*

In Japan, regional lymph nodes are classified as “compartment 1 to 3” according to tumor location, while distant lymph nodes are categorized as “compartment 4”. Based on this lymph node grading classification, the degree of lymph node metastasis (N classification) is defined as N0–N4. On the other hand, the UICC (International Union Against Cancer) TNM classification defines lymph nodes located in the defined area as “regional lymph nodes” regardless of the tumor location. The degree of lymph node metastasis is described as N0–N3 based on number of lymph node metastases. Metastasis to lymph nodes other than regional lymph nodes is categorized as M1.

2.2.1.1 Naming, number, range and boundary of regional lymph node

(1) Cervical lymph nodes (Fig. 2-15)

**Fig. 2-15:**
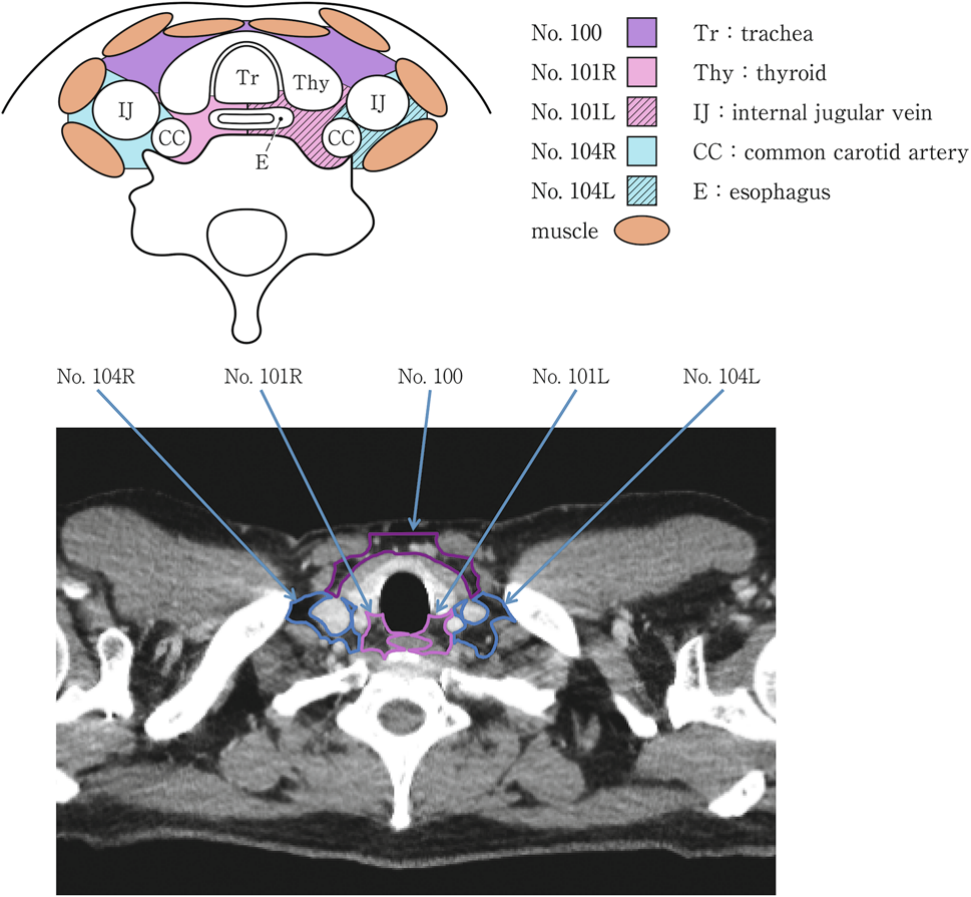
Cervical lymph nodes. *Tr* tracheal, *Thy* thyroid, *IJ* internal jugular vein, *CC* common carotid artery, *E* esophagus

No.100 Superficial lymph nodes of the neck

- The cervical lymph nodes except for the deep cervical nodes according to the General Rules for Clinical Studies on Head and Neck Cancer
- No.100spf Superficial cervical lymph nodes: Lymph nodes located along the external jugular veins and anterior jugular veins beneath the superficial cervical fascia.
- No.100sm Submandibular lymph nodes: Lymph nodes located around the submandibular glands and parotid glands, and anterior to the mylohyoid muscle.
- No.100tr Cervical pretracheal lymph nodes: Lymph nodes located in the pretracheal fatty tissue, extending from the hyoid bone superiorly, to the left brachiocephalic vein inferiorly, including the prethyroidal lymph nodes and the prelaryngeal lymph nodes.
- No.100ac Accessory nerve lymph nodes: Lymph nodes located along the accessory nerve(s), and anterior to the trapezius muscle.

No.101 Cervical paraesophageal lymph nodes

- Lymph nodes located around the cervical esophagus, including lymph nodes located along the recurrent laryngeal nerve and the cervical paratracheal lymph nodes. The lateral boundary is the medial border of the carotid sheath. A distinction between left and right must be included.

No.102 Deep cervical lymph nodes

- Lymph nodes located around the internal jugular vein and the common carotid artery.
- No.102up Upper deep cervical lymph nodes: Lymph nodes located from the caudal border of the digastric muscle superior to the carotid artery bifurcation.
- No.102mid Middle deep cervical lymph nodes: Lymph nodes located from the carotid artery bifurcation superiorly to the lower border of the cricoid cartilage inferiorly.

No.103 Peripharyngeal lymph nodes

- Lymph node located medial to the carotid sheath, extending from the caudal border of the digastric muscle superiorly to the lower border of the cricoid cartilage inferiorly. Postpharyngeal and parapharyngeal lymph nodes are included.

No.104 Supraclavicular lymph nodes

- Lymph nodes located in the supraclavicular fossa, extending from the lower border of the cricoid cartilage superiorly, to the clavicle inferiorly, including the lower internal deep cervical lymph nodes. The medial boundary is the medial border of the carotid sheath. A distinction between left and right must be included.

(2) Thoracic lymph nodes (Figs. 2-16, 2-17, 2-18, 2-19, 2-20, 2-21, 2-22, 2-23)

**Fig. 2-16:**
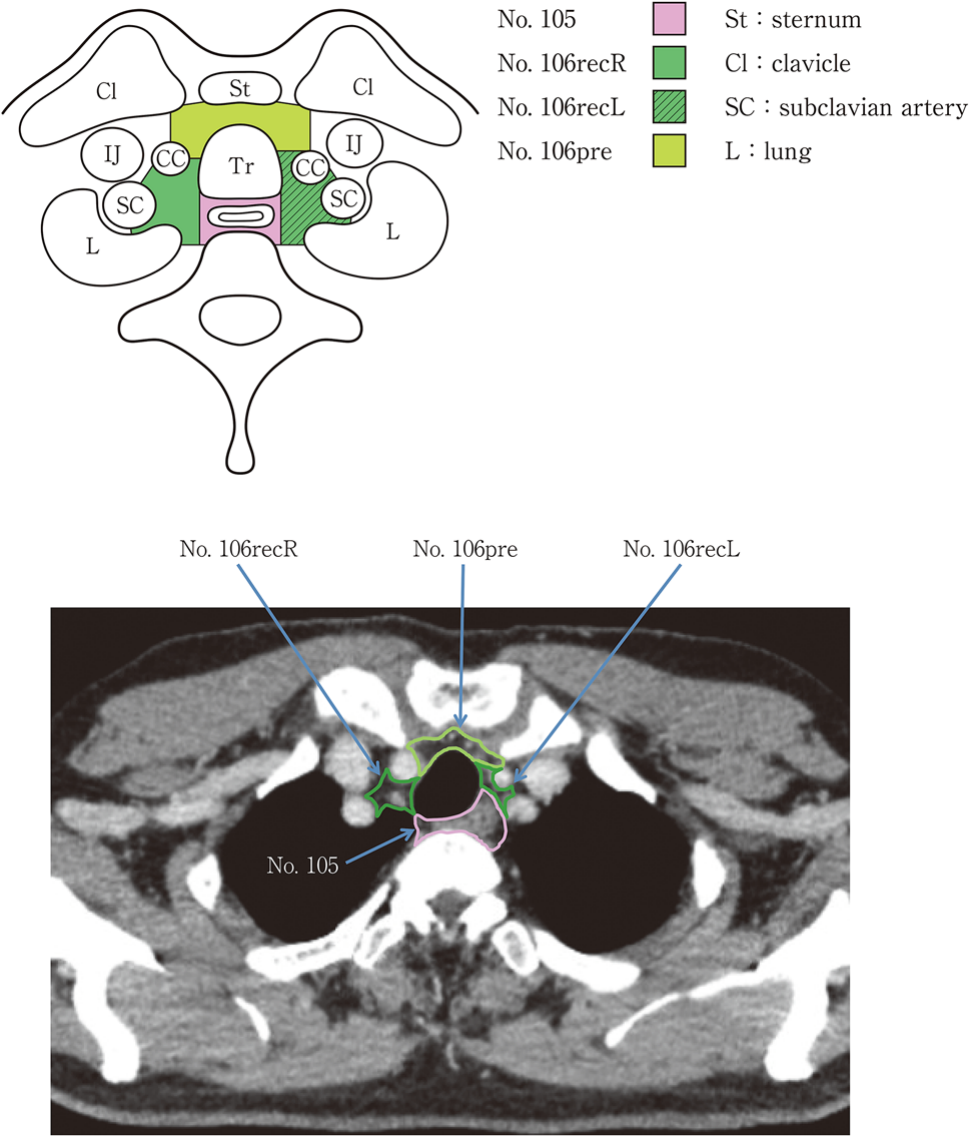
Superior mediastinal lymph nodes

**Fig. 2-17:**
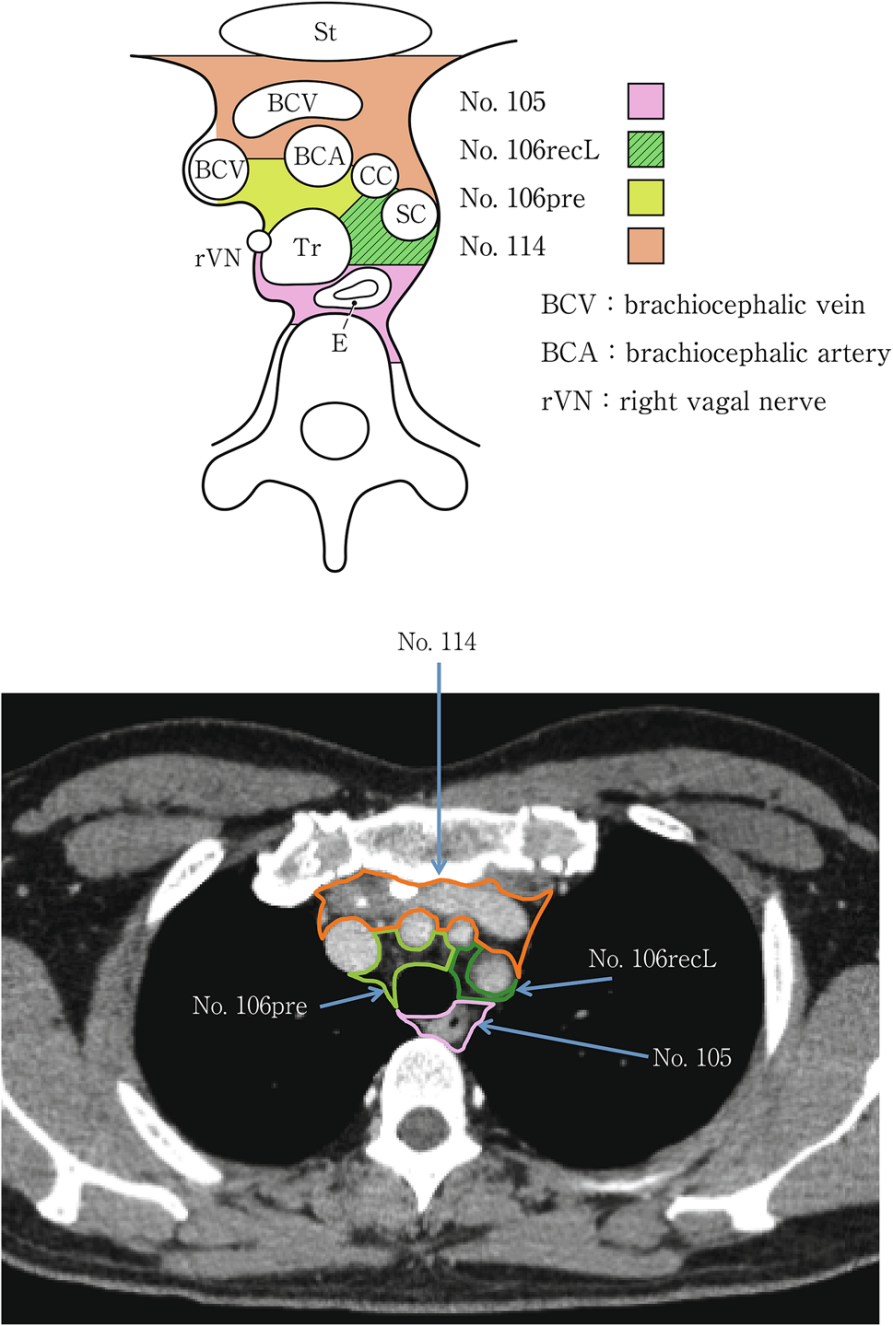
Upper mediastinal lymph nodes in the level above the aortic arch

**Fig. 2-18:**
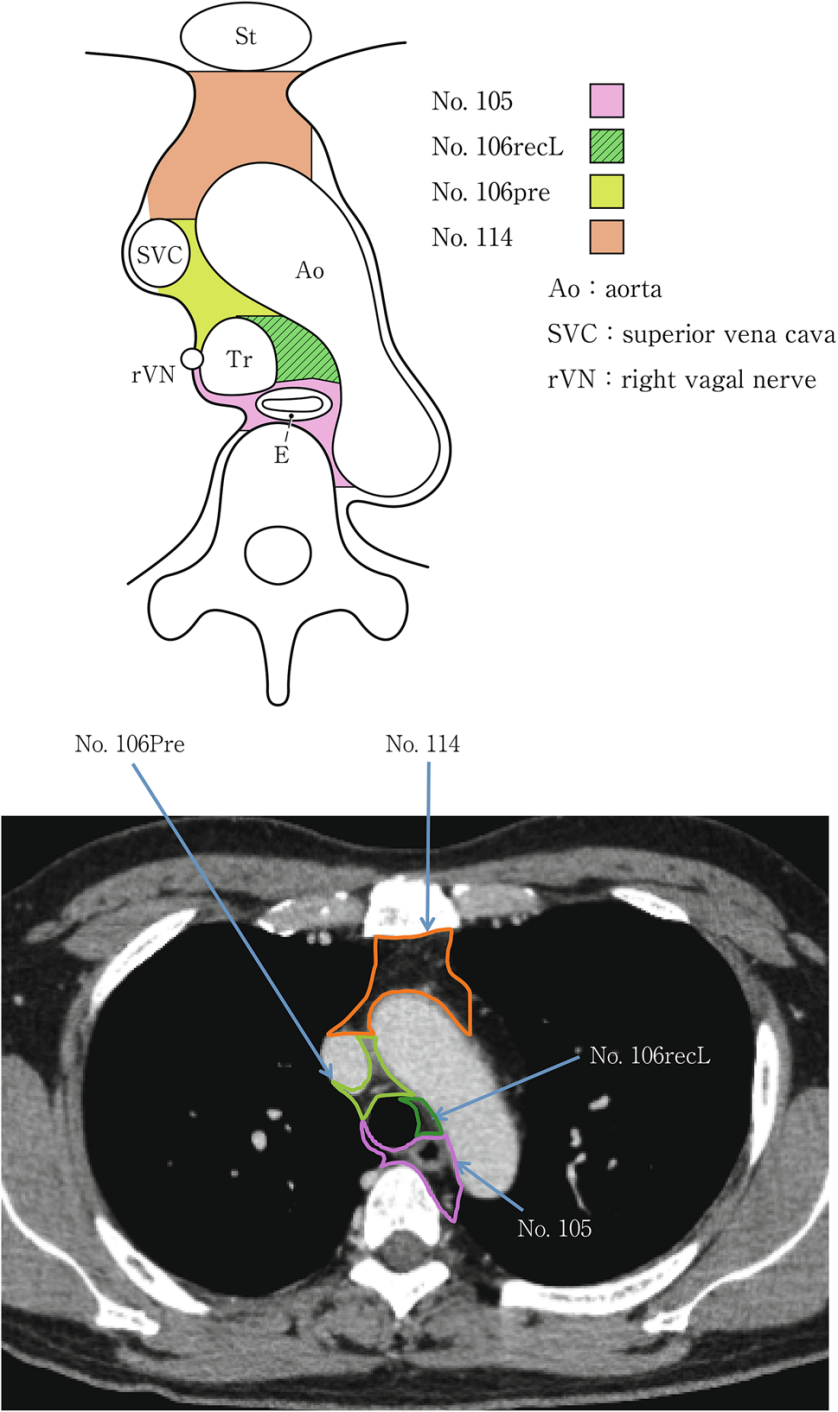
Upper mediastinal lymph nodes at the level of the aortic arch

**Fig. 2-19:**
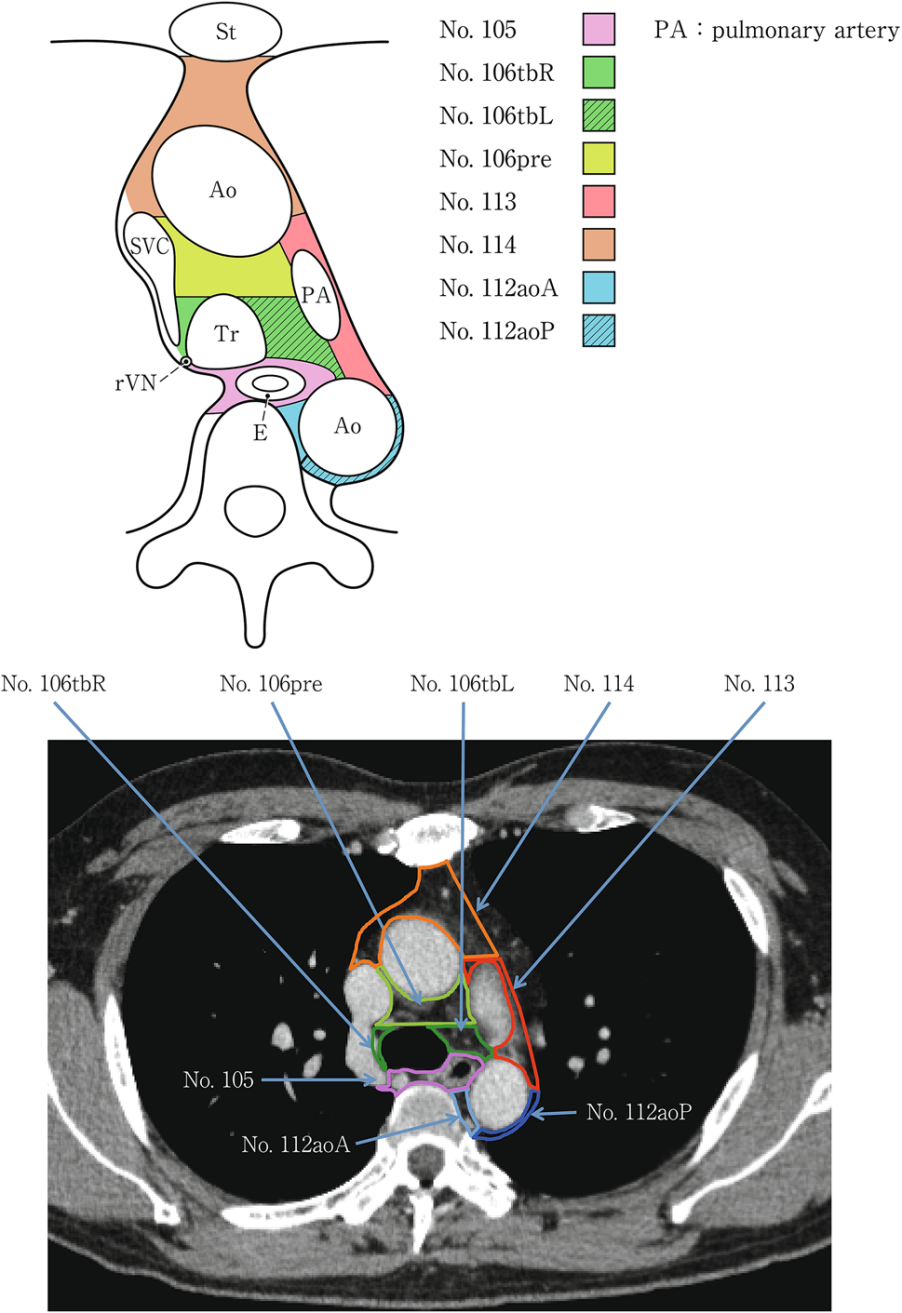
Upper mediastinal lymph nodes in the level below the aortic arch

**Fig. 2-20:**
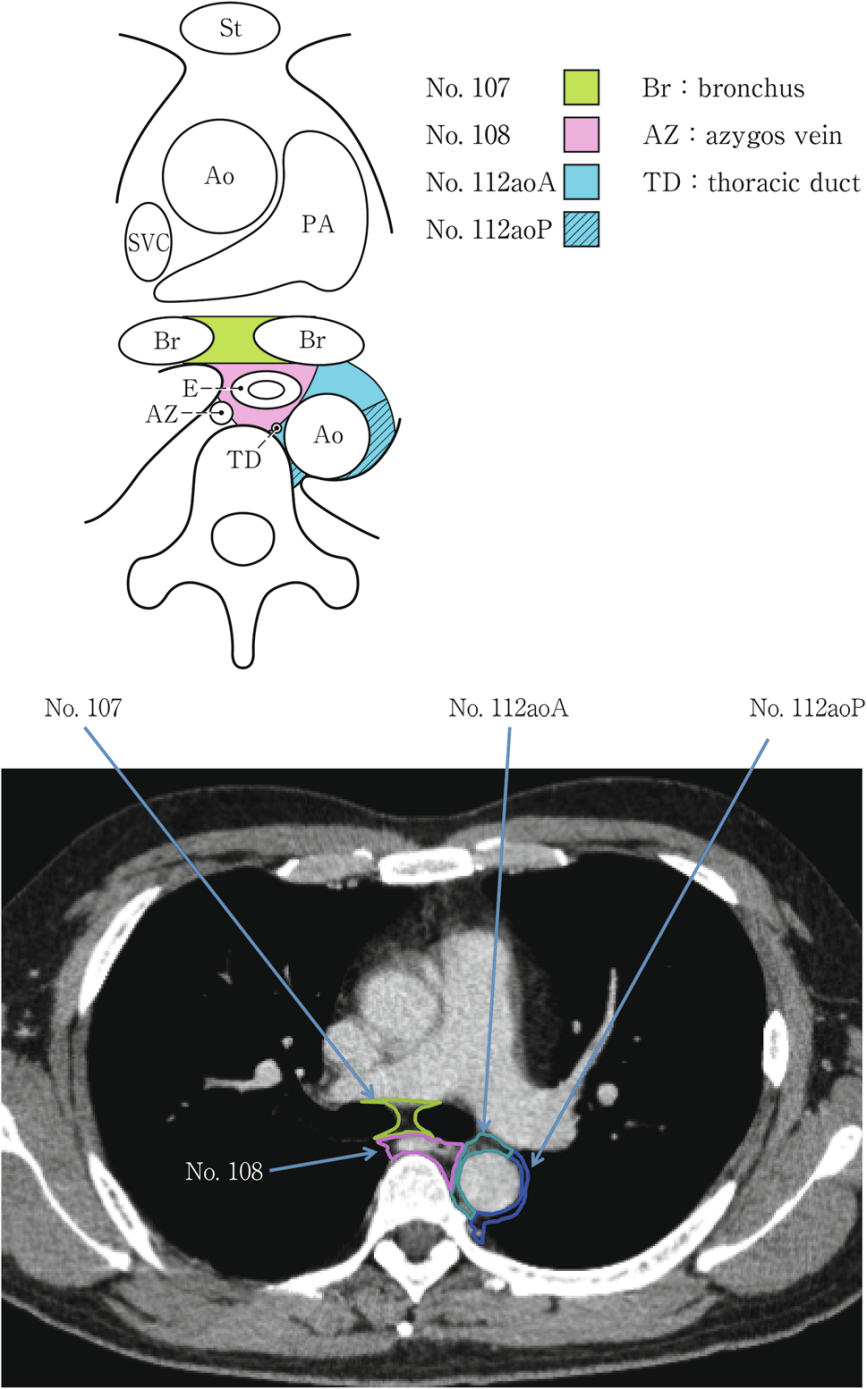
Mediastinal lymph nodes in the level below the carina

**Fig. 2-21:**
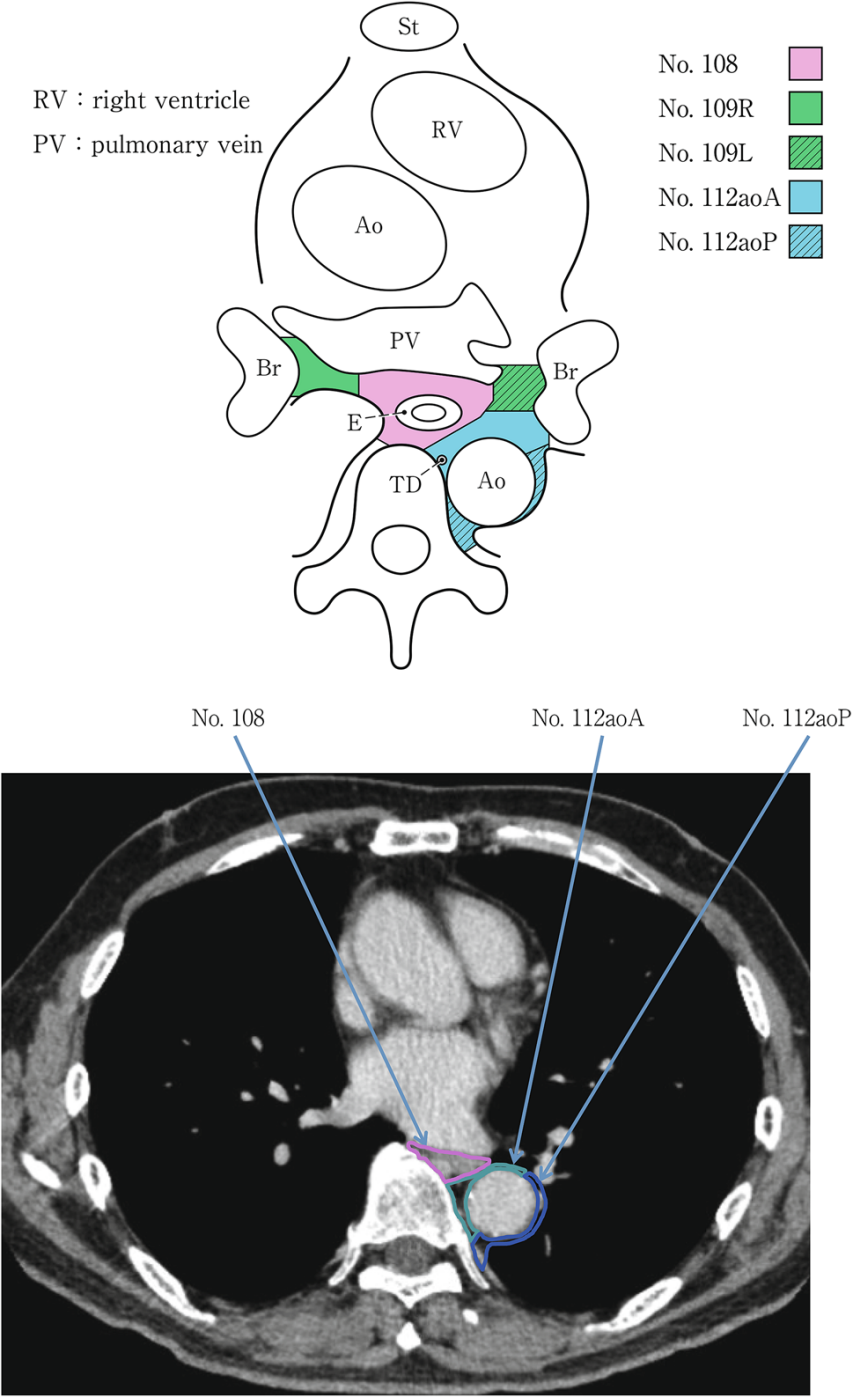
Lower mediastinal lymph nodes in the level of the inferior pulmonary vein

**Fig. 2-22:**
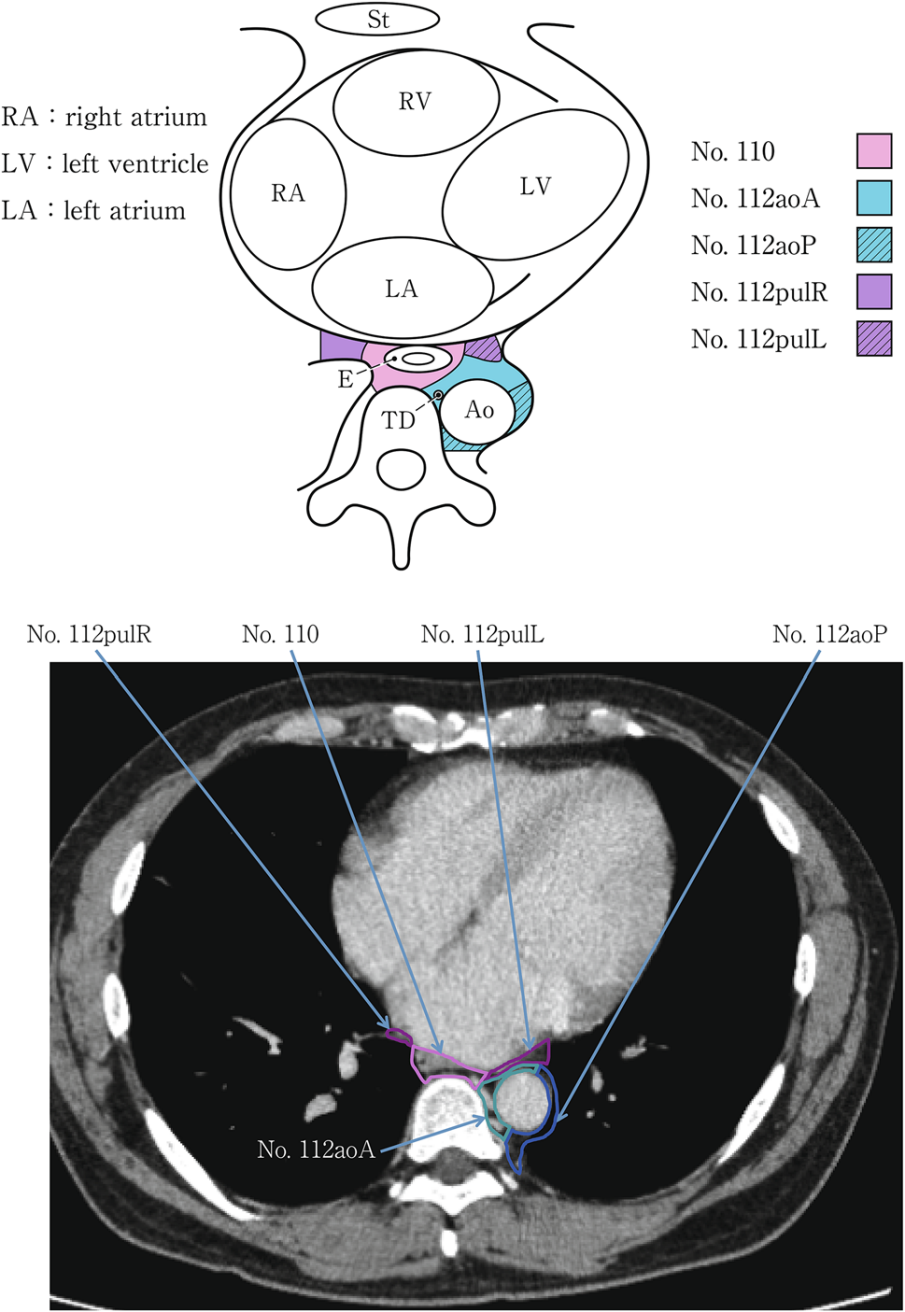
Lower mediastinal lymph nodes in the level of the right atrium

**Fig. 2-23:**
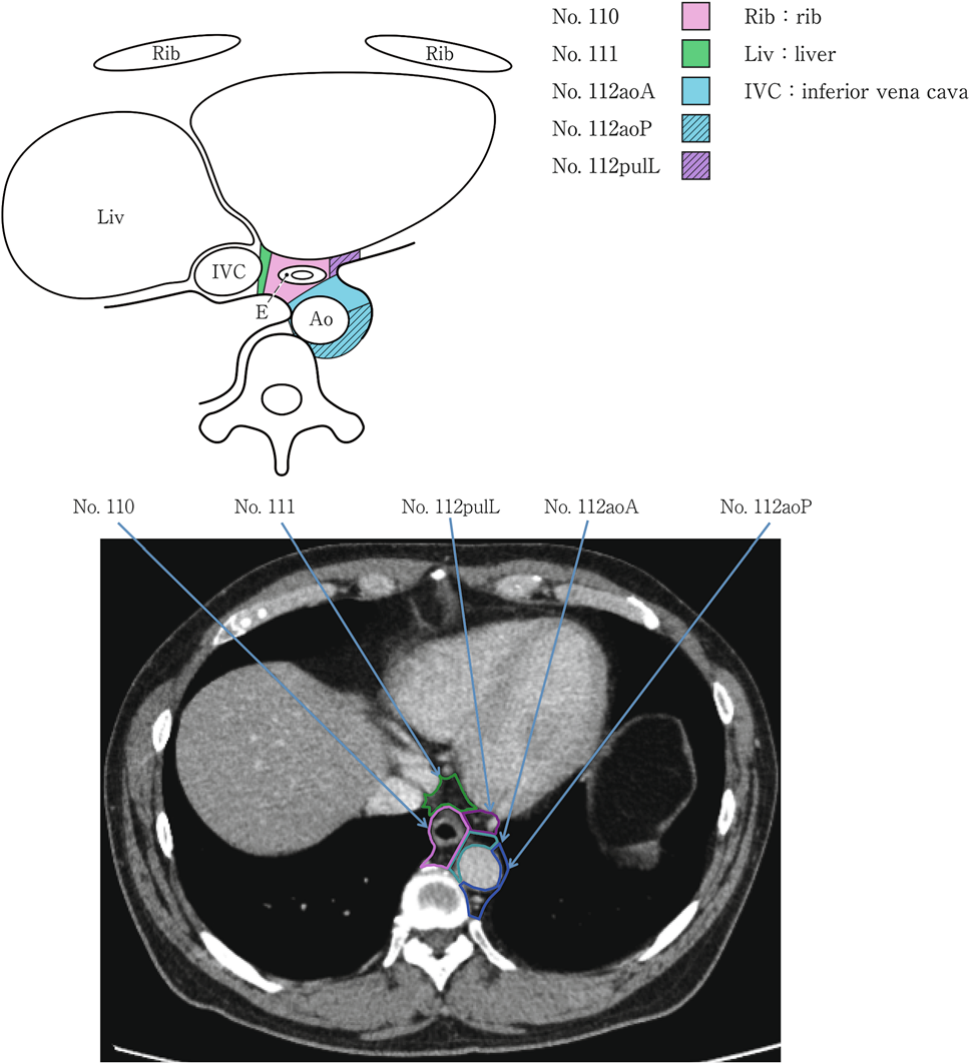
Lower mediastinal lymph nodes in the level above the hiatus

No.105 Upper thoracic paraesophageal lymph nodes

- Lymph nodes located around the upper thoracic esophagus posterior to the right vagus nerve on the right side. Lymph nodes located along the azygos vein arch and the right bronchial artery are included. The superior boundary is drawn from the cephalic border of the subclavian arteries to the suprasternal notch.

No.106 Thoracic paratracheal lymph nodes

- Lymph nodes located along the anterior and lateral wall of the thoracic trachea.
- No.106rec Recurrent nerve lymph nodes: Lymph nodes located along the recurrent laryngeal nerves in the mediastinum. The superior boundary is drawn from the cephalic border of the subclavian arteries to the suprasternal notch, and the inferior boundary is the caudal border of the recurrent laryngeal nerve curving upward on both sides.
- No.106recL Left recurrent nerve lymph nodes
- Lymph nodes located along the left recurrent laryngeal nerve
- No.106recR Right recurrent nerve lymph nodes
- Lymph nodes located along the right recurrent laryngeal nerve

No.106pre Pretracheal lymph nodes

- Lymph nodes located in front of the anterior wall of the thoracic trachea, and anterior to the right vagus nerve.
  - No.106tb Tracheobronchial lymph nodes
    - Lymph nodes located in the tracheobronchial angle.
    - No.106tbL Left tracheobronchial lymph nodes: The superior border is the inferior wall of the aortic arch, and the lymph nodes are located in the area surrounded by the medial wall of the aortic arch.
    - No.106tbR Right tracheobronchial lymph nodes: The superior border is the inferior wall of the azygos vein.
  - No.107 Subcarinal lymph nodes
    - Lymph nodes located caudal to the carina of the trachea. The lateral boundaries are the extended line of both lateral margins of the trachea.
  - No.108 Middle thoracic paraesophageal lymph nodes
    - Lymph nodes located around the middle thoracic esophagus.
  - No.109 Main bronchus lymph nodes
    - Lymph nodes located in the caudal area of the main bronchus. The internal boundary is the border of subcarinal lymph nodes, and the external boundary is the lung.
  - No.110 Lower thoracic paraesophageal lymph nodes
    - Lymph nodes located around the lower thoracic esophagus.
  - No.111 Supradiaphragmatic lymph nodes
    - Lymph nodes located in the area surrounded by the diaphragm, pericardium, and esophagus.
  - No.112 Posterior mediastinal lymph nodes: Lymph nodes located in the area surrounded by the descending aorta, inferior pulmonary vein and pericardium. These lymph nodes are divided into the following subgroups.
    - No.112aoA Anterior thoracic paraaortic lymph nodes: Among lymph nodes located around the descending aorta, lymph nodes existing on the same side of the esophagus, including lymph nodes along the thoracic duct.
    - No.112aoP Posterior thoracic paraaortic lymph nodes: Among lymph nodes located around the descending aorta, lymph nodes existing on the opposite side of the esophagus.
    - No.112pul pulmonary ligament lymph nodes: Lymph nodes located in the pulmonary ligament(s), including lymph nodes adjacent to the pericardium and the inferior pulmonary vein. A distinction between left and right must be included.
  - No.113 Ligamentum arteriosum lymph nodes (Botallo lymph nodes)
    - Lymph nodes located on the left side of the arterial ligament.
  - No.114 Anterior mediastinal lymph nodes
    - Lymph nodes located anterior to the superior vena cava, including lymph nodes of the brachiocephalic venous angle and lymph nodes around the thymus gland.

(3) Abdominal lymph nodes (Fig. 2-24)

**Fig. 2-24:**
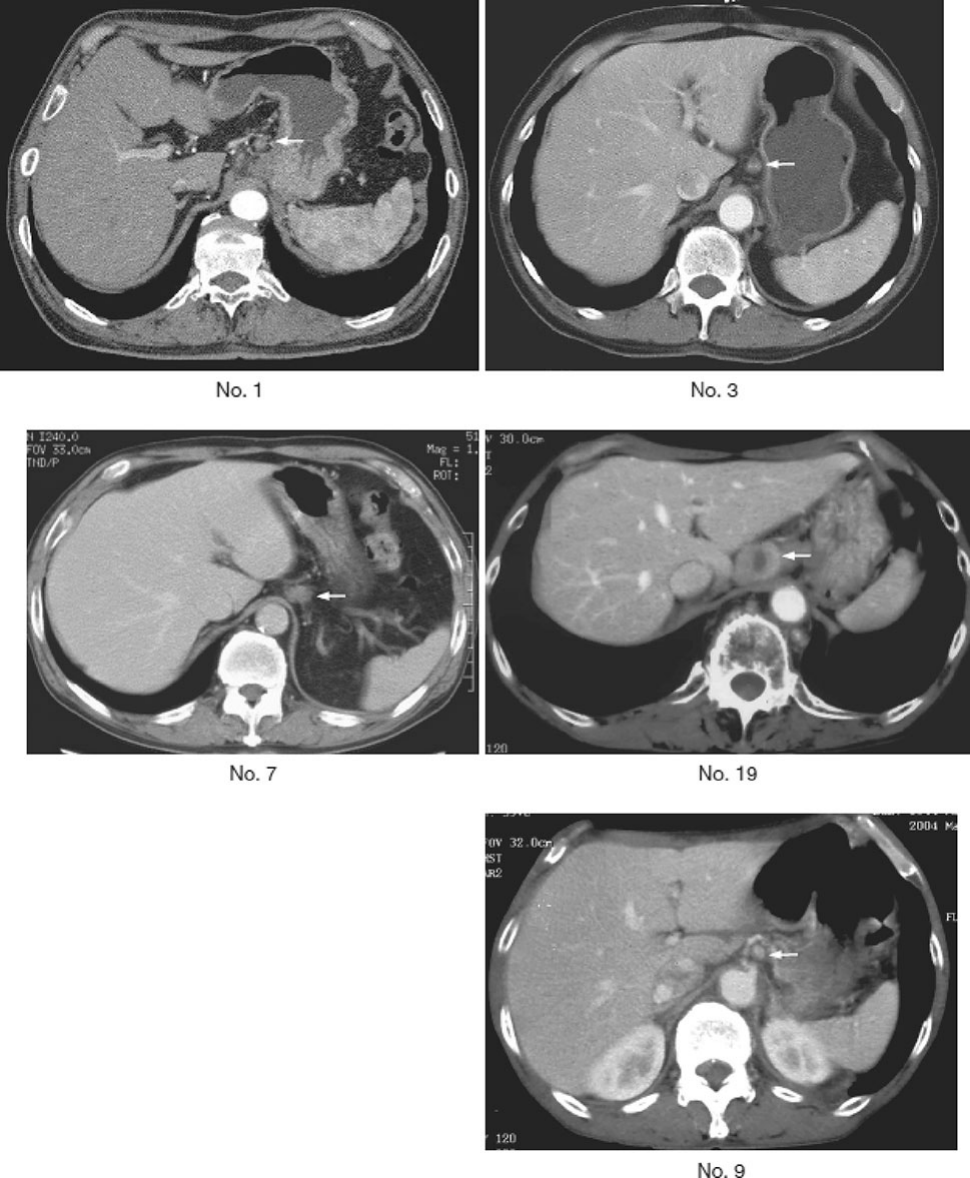
Abdominal lymph nodes

The names, numbers and extent of abdominal lymph nodes are defined by the “Japanese Classification of Gastric Cancer”.

[References]

(1) Japan Society for Head and Neck Cancer. General Rules for Clinical Studies on Head and Neck Cancer (in Japanese). 5th ed. Kanehara Shuppan, Toyko, 2012

(2) Japanese Gastric Cancer Association. Japanese Classification of Gastric Carcinoma (in Japanese). 14th ed. Kanehara Shuppan, Tokyo, 2010

2.2.1.2 Lymph node groups

In cervical esophageal cancer, lymph nodes No.105 and No.106rec are defined as those present in an area that could be dissected through a cervical incision.

2.4 Multiple primary cancers

Synchronous tumors: diagnosed within a period of 1 year.

Metachronous tumors: diagnosed after an interval of 1 year or more.

Cases with both synchronous and metachronous tumors are defined as synchronous/metachronous tumors.

3.4.3 Lymph node dissection

3.4.3.1 Types of lymph node dissection

Cervical lymph node dissection indicates bilateral dissection including No.101 and No.104.

3.7 Curativity

Curativity for transhiatal esophagectomy

The extent of lymph node dissection is defined as D0 after a transhiatal esophagectomy.

In cases with cN0 and cM0, curability is determined based on the pathological depth of tumor invasion.

pT1a (EP or LPM), D0 and pR0: fCurA.

pT1a-MM, pT1b, pT2, pT3, D0 and pR0: fCurB.

4.2.1. Histological classification

4.2.1.1. Benign epithelial neoplasms

1. Squamous cell papilloma

Squamous cell papilloma shows papillary growth of squamous epithelium with no atypism. This tumor occasionally has clear cytoplasm with vacuoles, suggesting papilloma virus infection. It is important that this lesion should be differentiated from verrucous carcinoma in biopsy specimens.

2. Adenoma

Adenoma is a rare neoplasm in squamous-lined esophagus. A few reports have described esophageal adenoma arising from a proper esophageal gland or its duct.

- Note: Several cases of a peculiar type of benign tumor in Barrett esophagus have been reported. However, there is no consensus regarding the diagnostic criteria or the methods of description for adenoma in Barrett esophagus.

*4.2.1.2. Intraepithelial neoplasia*

1. Squamous intraepithelial neoplasia (Fig. 2-25)

**Fig. 2-25:**
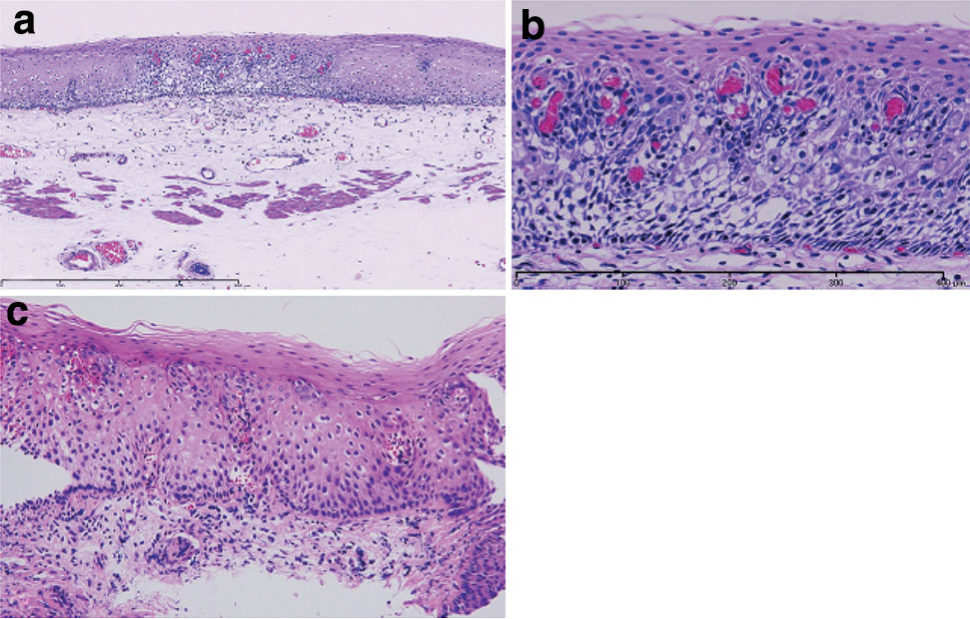
Squamous intraepithelial neoplasia. **a** Low magnification of squamous intraepithelial neoplasia. The lesion is visible as an iodine-unstained area measuring 3 mm in size. Histologically, the lesion is well demarcated. **b** High magnification. The tumor shows mild nuclear atypia with low cellular density and a regular arrangement of the basal layer. **c** Histology of biopsy specimen. The lesion is visible as an iodine-stained tan area measuring 5 mm in size. Histopathologically, a mildly thickened epithelium exhibits atypical cell proliferation in the lower two-thirds of the epithelium, but nuclear atypia is mild and the basal layer has a regular arrangement

Intraepithelial neoplasia is defined as a lesion showing structural and cytological abnormalities that can be regarded as indicating a neoplasm; however, it does not include carcinoma in situ.

- Note: There are two types of intraepithelial neoplasia; intraepithelial neoplasia of the squamous epithelium and intraepithelial neoplasia of the columnar epithelium in Barrett esophagus. The details of the intraepithelial neoplasia of the squamous epithelium are explained below. Intraepithelial neoplasia of columnar epithelium is described in the section on esophageal adenoma.

Explanation

1. Intraepithelial neoplasia is a neoplastic lesion that exhibits structural and cytological abnormalities but does not include carcinoma. Previously, this lesion was formally known as dysplasia, but it has been referred to as intraepithelial neoplasia since the publication of the WHO classification (2000) and the 10th edition of the Japanese classification (2008). Intraepithelial neoplasia has been classified into two groups: low grade and high grade. In the 10th edition, high-grade intraepithelial neoplasia included lesions that could be diagnosed as squamous cell carcinoma in situ in Japan. Since the 11th edition includes the diagnosis of squamous cell carcinoma in situ, the previous two-tier subclassification of low grade and high grade has been abolished.

2. Of note, a peculiar type of squamous cell carcinoma in situ mimicking a “low-grade intraepithelial neoplasia” according to the diagnostic criteria of the 10th edition has been reported. Although such tumor cells differentiate towards the epithelial surface, the cell density (cellularity) is increased and the cell arrangement (polarity) is irregular in the basal and parabasal layers of the epithelium. Cellularity in the epithelial surface is also increased, compared with non-neoplastic lesions. The nuclei of the tumor cells are uniform. However, they are occasionally large. Iodine-unstained lesions more than 10 mm in diameter mimicking low-grade intraepithelial neoplasia should be suspected as being a carcinoma in situ.

- Note: Differential diagnoses of intraepithelial neoplasia include lesions that exhibit reactive atypism as a result of inflammation and regeneration. For instance, regenerative squamous epithelium in cases with reflux esophagitis sometimes exhibits atypical basal or parabasal cells, but these lesions should not be diagnosed as intraepithelial neoplasia. Lesions in which the differentiation of neoplasm from reactive changes is difficult should be diagnosed as “atypical epithelium” or “atypical epithelium, indefinite for neoplasia”.

4.2.1.3. Malignant epithelial neoplasms

Histological subtyping should be done based on the predominant histological features of the tumor.

1. Squamous cell carcinoma (Figs. 2-26, 2-27, 2-28, 2-29, 2-30)

**Fig. 2-26:**
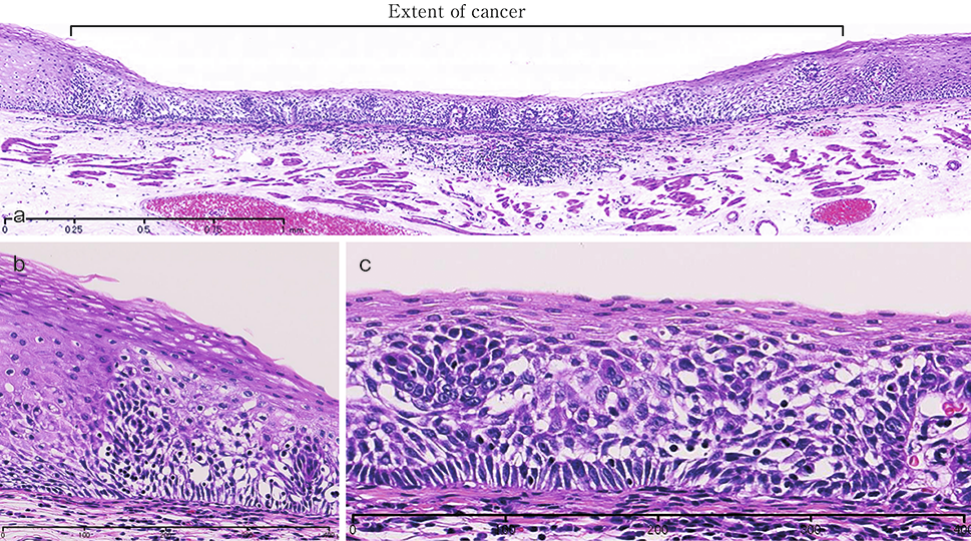
Squamous cell carcinoma (pT1a-EP). **a** Low-power view of squamous cell carcinoma. The border between the carcinoma and non-neoplastic epithelium is clear. **b** Histology of the border between the squamous cell carcinoma and the non-neoplastic squamous epithelium. The cancer tissue exhibits a high cellular density with a loss of the basal layer. **c** Histology of the central portion of cancer tissue. The cancer cells have proliferated throughout the entire epithelial layer, but have not invaded the lamina propria mucosae

**Fig. 2-27:**
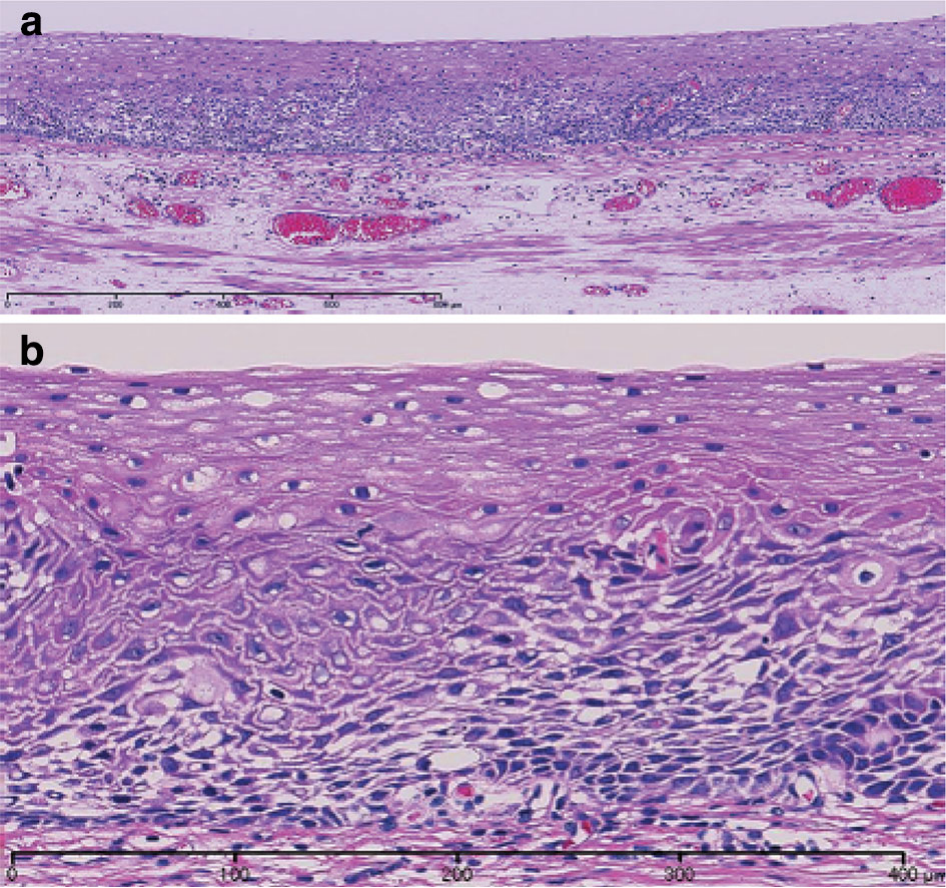
Basal layer-type squamous cell carcinoma (pT1a-EP). **a** Cancer cells are present within the *lower half* of the epithelium, whereas squamous epithelial cells with minimal atypia are present in the *upper half*. **b** High-power view of the above figure. The basal cells have disappeared and the *lower half* of the epithelium has been replaced by cancer cells with nuclear atypia and a *higher* cellular density. Squamous epithelial cells in the *upper half* are small in size and have a *lower* cellular density

**Fig. 2-28:**
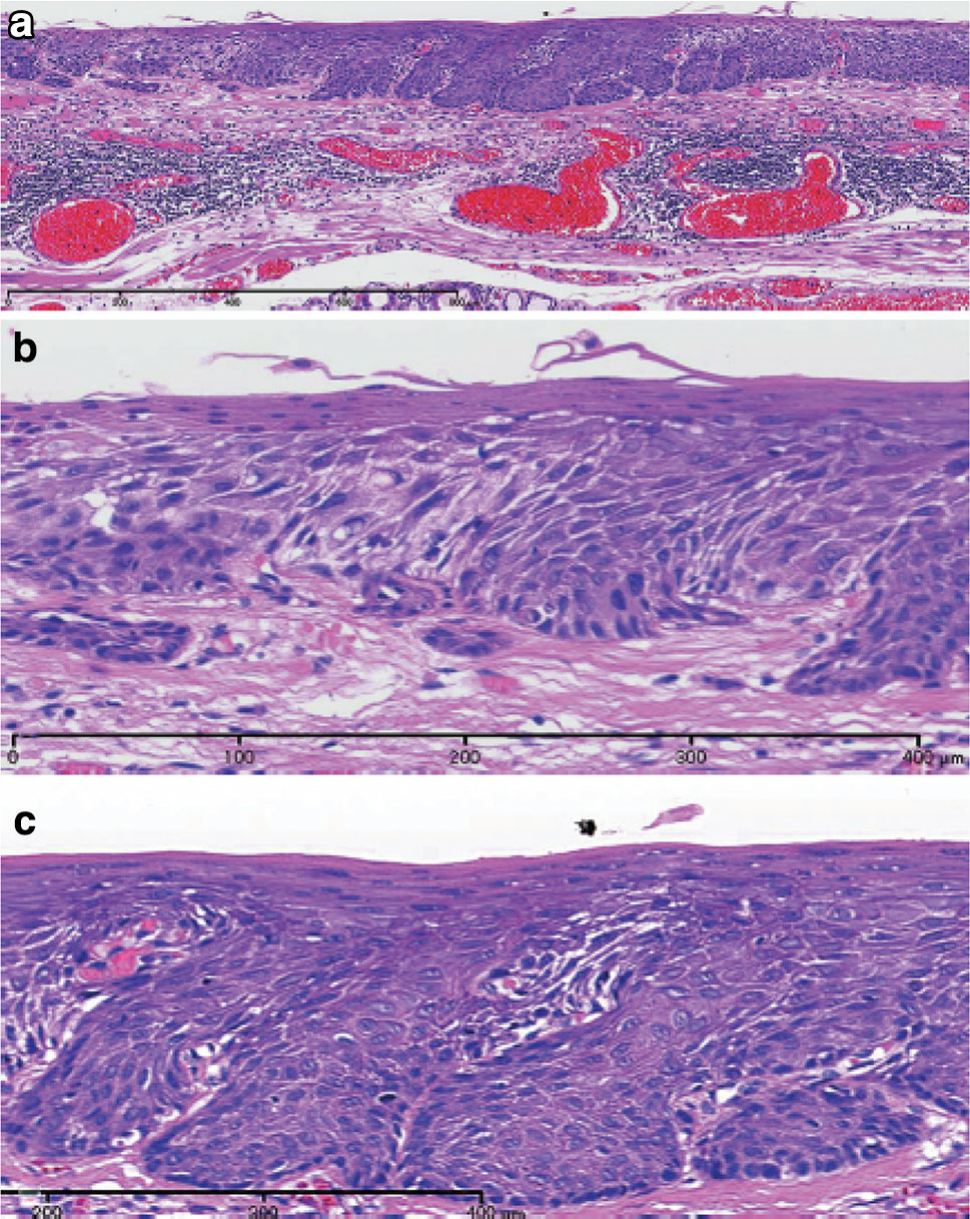
Squamous cell carcinoma with invasion into the lamina propria mucosae (pT1a-LPM). **a** Squamous cell carcinoma shows mild thickening and irregular downward growth. **b** Droplet infiltration is observed in the lamina propria mucosae. **c** Expansive growth of squamous cell carcinoma

**Fig. 2-29:**
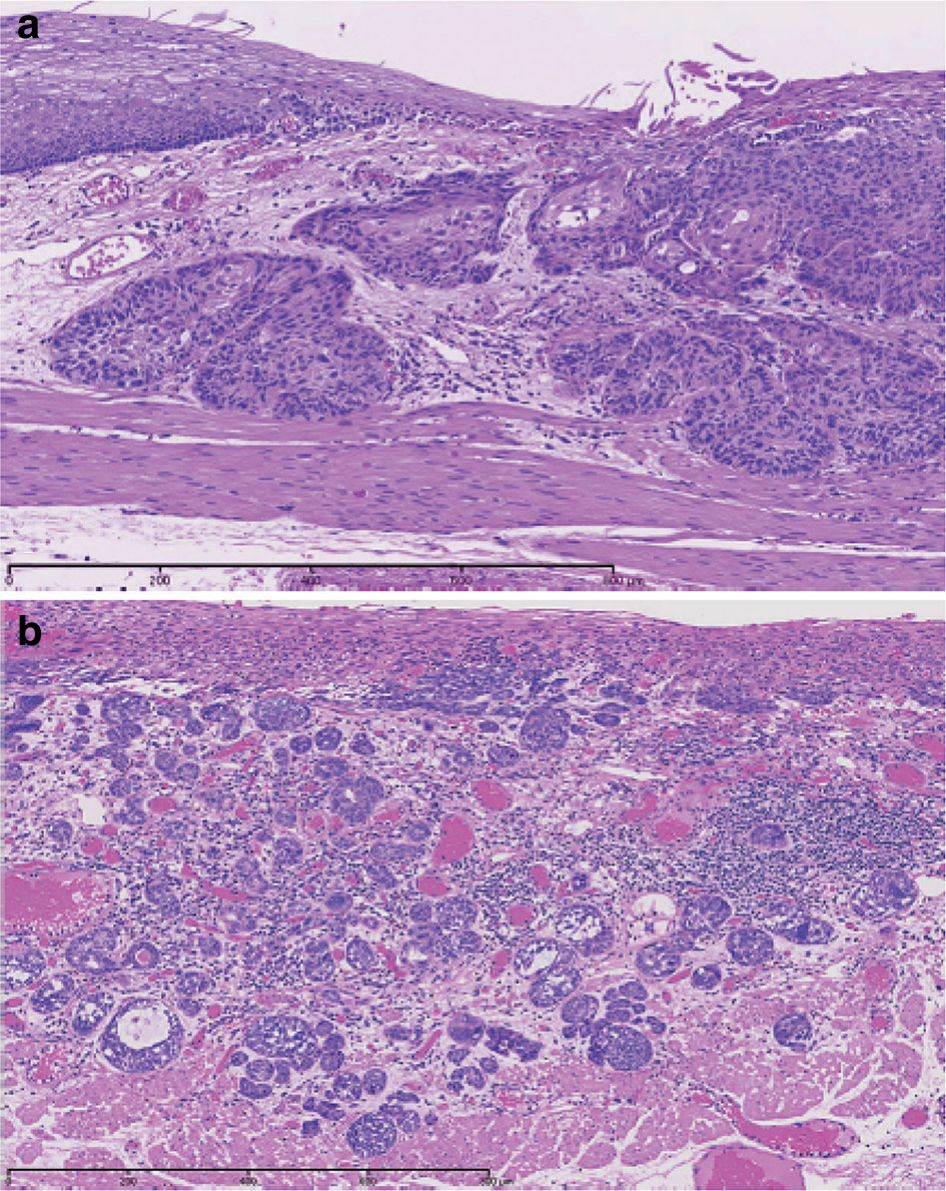
Squamous cell carcinoma with invasion into the muscularis mucosae (pT1a-MM). **a** Cancer cells have reached the upper end of the muscularis mucosae and have partly invaded the muscularis mucosae. Both situations are classified as pT1a-MM. **b** Cancer cells have invaded the muscularis mucosae, but not beyond

**Fig. 2-30:**
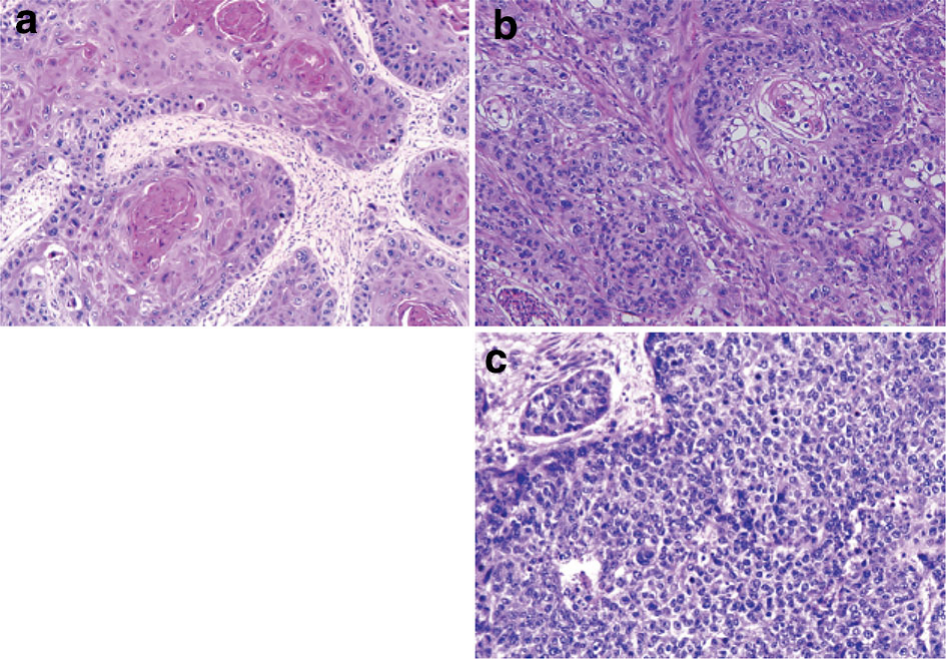
Squamous cell carcinoma. **a** Well differentiated squamous cell carcinoma: cancer pearls with marked keratinization are observed. **b** Moderately differentiated squamous cell carcinoma: sheet-like arrangement of tumor cells with slight keratinization is observed. **c** Poorly differentiated squamous cell carcinoma: keratinization is not observed, although tumor cells show sheet-like arrangement

Squamous cell carcinoma in situ is equivalent to squamous cell carcinoma showing pT1a-EP. Invasive squamous cell carcinoma forms solid nests of tumor cells that differentiate toward stratified squamous epithelium. Keratinization, stratification, and intercellular bridges are frequently observed. A few tubules and a small amount of epithelial mucus may exist in the tumor; these features should be noted, if present.

- Note: Squamous cell carcinoma in situ can be diagnosed based on structural and cytological atypia. Structural atypia includes cellular density (cellularity), cell differentiation, loss of polarity at the basal layer, etc. Cytological atypia includes variations in nuclear size and shape, hyperchromasia, loss of polarity, prominent nucleoli, an increased nuclear/cytoplasmic ratio, and mitoses. There is no need to describe the degree of differentiation in squamous cell carcinoma in situ. Invasive squamous cell carcinoma is subclassified into three groups based on squamous differentiation: well differentiated, moderately differentiated, and poorly differentiated. Verrucous carcinoma, a type of very well differentiated squamous cell carcinoma with papillary growth, is a rare tumor in the esophagus (Fig. 2-31). Poorly differentiated squamous cell carcinoma can be composed of spindle-shaped cells and may resemble sarcoma; such lesions should be classified as spindle cell carcinoma or a spindle cell variant of SCC.

**Fig. 2-31:**
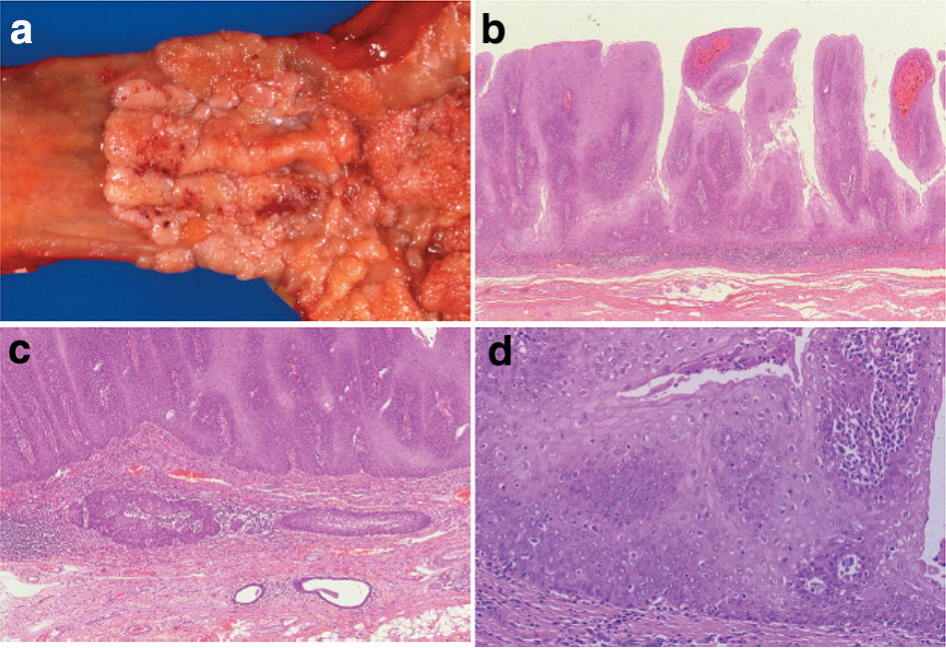
Verrucous squamous carcinoma. **a** Gross features of verrucous carcinoma. The tumor involves the cervical esophagus and lower pharynx. The tumor has a granular surface. **b** Low-power view of verrucous carcinoma. Note that papillary growth is prominent and the basal layer is generally flat. **c** Verrucous carcinoma with invasion into the lamina propria mucosae. **d** High-power view of verrucous carcinoma. The tumor has a high cellular density with an irregular arrangement at the basal site. However, squamous differentiation is apparent towards the surface

2. Basaloid (-squamous) carcinoma (Fig. 2-32)

**Fig. 2-32:**
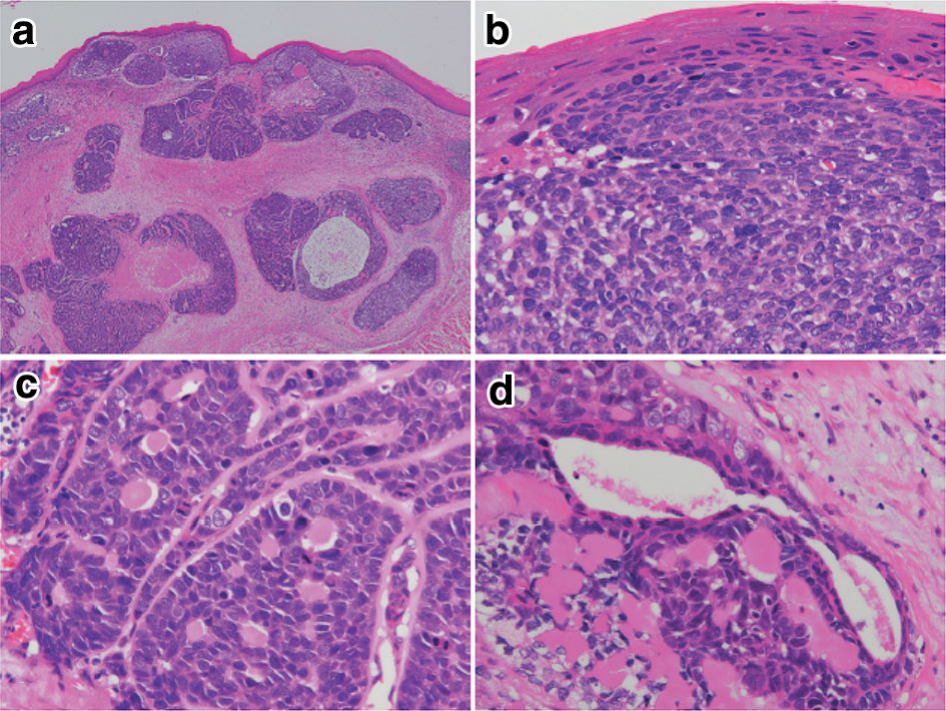
Basaloid squamous carcinoma. **a** Basaloid squamous carcinoma is often covered by non-neoplastic stratified squamous epithelium and grows downwards. The tumor forms a solid nest with occasional cyst formation and necrosis. **b** Tumor cells similar to basal cells form solid nests in various sizes under stratified squamous epithelium. **c** Tumor cells show a solid and trabecular arrangement. Eosinophilic basement membrane-like material deposits are present around and within the tumor nest. **d** Basaloid squamous carcinoma sometimes contains duct-like differentiation

Basaloid (-squamous) carcinoma consists of relatively small cells that resemble basal cells and that grow in a solid or trabecular pattern, occasionally forming irregular adenoid or small cystic structures. This entity is also characterized by hyalinization both within and outside tumor nests, and the hyaline material is the same as that in the basement membrane. Duct-like structures may be observed. Squamous cell carcinoma frequently coexists with basaloid squamous carcinoma in the intraepithelial area and is occasionally present in invasive areas.

3. Carcinosarcoma (Fig. 2-33)

**Fig. 2-33:**
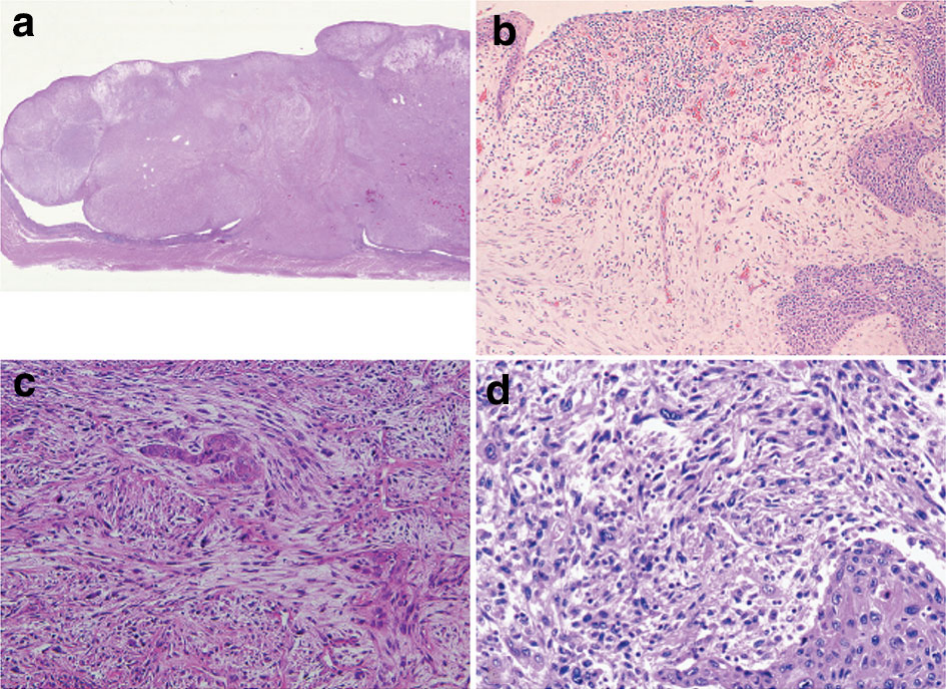
Carcinosarcoma. **a** A large tumor protrudes into the esophageal lumen. The tumor has not invaded deeply, in comparison with its size. **b** The polypoid tumor mainly consists of spindle cells, and its surface is covered by squamous cell carcinoma. **c** A large part of the tumor (sarcomatous component) is occupied by spindle-shaped tumor cells with scattered small foci of squamous cell carcinoma. **d** Tumor cells with prominent polymorphous nuclei are present in the sarcomatous component, which is similar to pleomorphic undifferentiated sarcoma (formerly diagnosed as malignant fibrous histiocytoma [MFH])

Carcinosarcoma is composed of both carcinomatous and sarcoma-like components. These tumors include carcinoma consisting of spindles or polymorphous tumor cells with a mesenchymal character, and they can contain neoplastic bone and cartilage components. When a tumor is accompanied by various differentiations such as chondrosarcoma and osteosarcoma, each component should be noted in the pathological diagnosis. This tumor often shows polypoid growth with a stalk, characterized by the presence of squamous cell carcinoma in situ surrounding the stalk. Tumors showing prominent proliferation of reactive mesenchymal cells should also be included in carcinosarcoma.

4. Adenocarcinoma (Fig. 2-34)

**Fig. 2-34:**
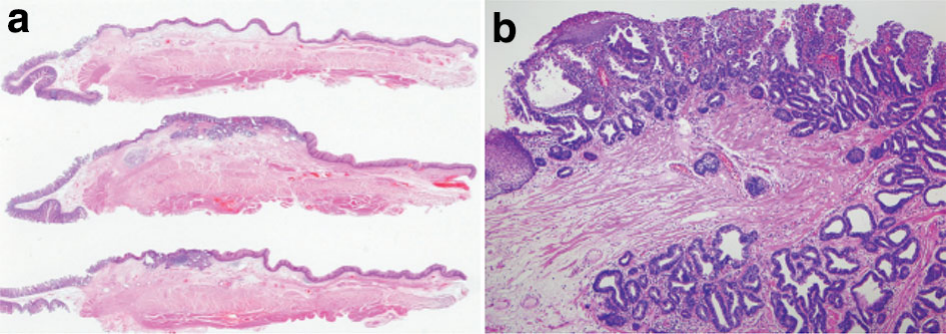
Adenocarcinoma in non-Barrett esophagus. **a** Well differentiated adenocarcinoma is observed proximal to the squamocolumnar junction. **b** Well differentiated adenocarcinoma is present beneath the squamous epithelium. Barrett mucosa is not observed, so that the origin of the tumor is unknown

Esophageal adenocarcinoma should be classified in the same manner as gastric carcinoma. Most tumors arise from Barrett esophagus. Esophageal adenocarcinoma rarely arises from ectopic gastric mucosa.

- Note: In adenocarcinoma located predominantly in the lower esophagus, if the tumor is considered to be gastric cancer with esophageal invasion, its possible origin should be noted in the pathological diagnosis.

5. Adenosquamous carcinoma (Fig. 2-35)

**Fig. 2-35:**
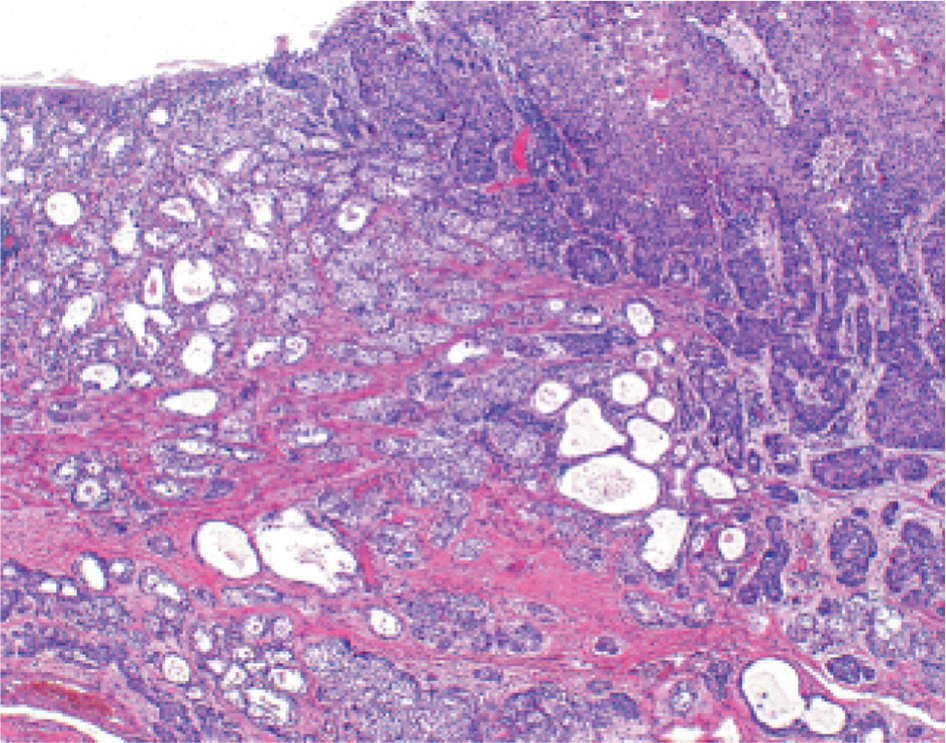
Adenosquamous carcinoma. Adenosquamous carcinoma consists of squamous cell carcinoma and adenocarcinoma. Invasive squamous cell carcinoma is present on the oral side of the tumor, and adenocarcinoma is mainly present on the anal side

Adenosquamous carcinoma has both components of adenocarcinoma and squamous cell carcinoma, each component of which can be easily recognized. When either of the two components is limited to a very small area (less than 20%), the tumor should be classified as a major component, with an additive note regarding the minor component (e.g. squamous cell carcinoma with an adenocarcinoma component).

6. Mucoepidermoid carcinoma (Fig. 2-36)

**Fig. 2-36:**
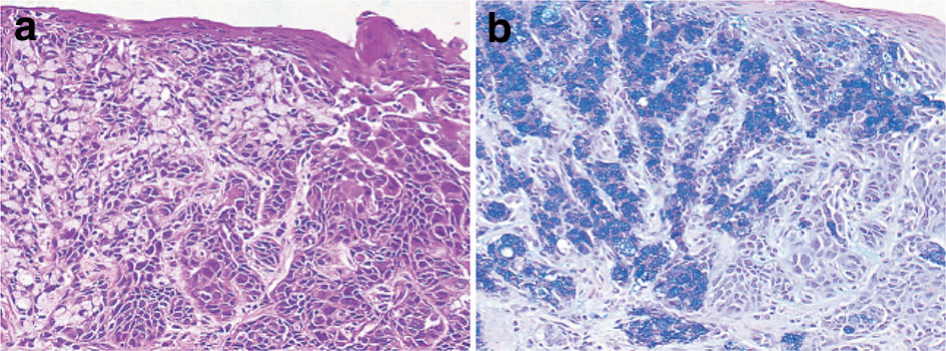
Mucoepidermoid carcinoma. **a** Signet ring cell carcinoma is observed within squamous cell carcinoma. **b** Mucus in signet ring cell carcinoma is stained *blue* by Alcian-blue staining. Serial section of ** a**

Mucus-containing cells (adenocarcinoma cells) are present within nests of squamous cell carcinoma. There are usually no distinct tubular structures. The mucus-containing cells may be goblet or signet ring cell type. Mucus is occasionally discharged into the stroma and intercellular spaces.

7. Adenoid cystic carcinoma (Fig. 2-37)

**Fig. 2-37:**
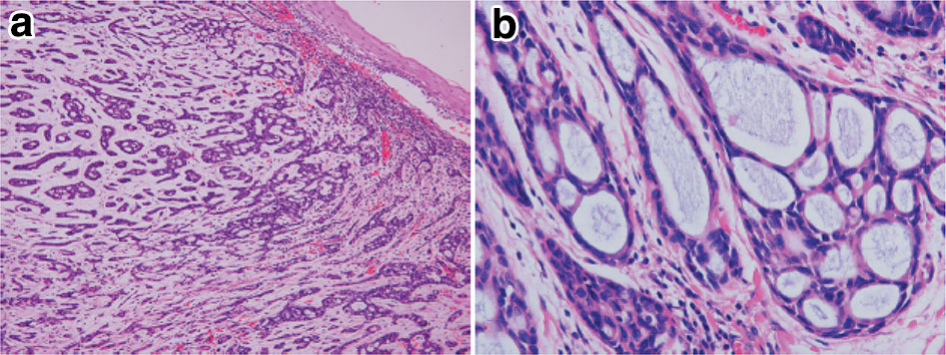
Adenoid cystic carcinoma. **a** A tumor with duct-like structures and a cribriform pattern has invaded downwards. **b** The histology of esophageal adenoid cystic carcinoma is almost the same as that with a salivary gland origin. Nuclear atypia is more prominent in the esophageal tumor than in the salivary gland tumor

Adenoid cystic carcinoma is a very rare tumor with the same histological appearance as a salivary gland. Small cells with scanty cytoplasm form cribriform structures, solid nests or trabecular structures. Mucus within small cystic spaces inside and outside the tumor nests stains light blue with Alcian blue-PAS. There is no epithelial mucus production in the cribriform areas, unlike in adenocarcinoma with a cribriform pattern. A pattern of small duct-like structures is occasionally seen in which ducts consist of tumor cells aligned in a double layer containing epithelial mucus. This tumor should be carefully distinguished from basaloid squamous carcinoma.

8. Neuroendocrine cell tumor (Fig. 2-38)

**Fig. 2-38:**
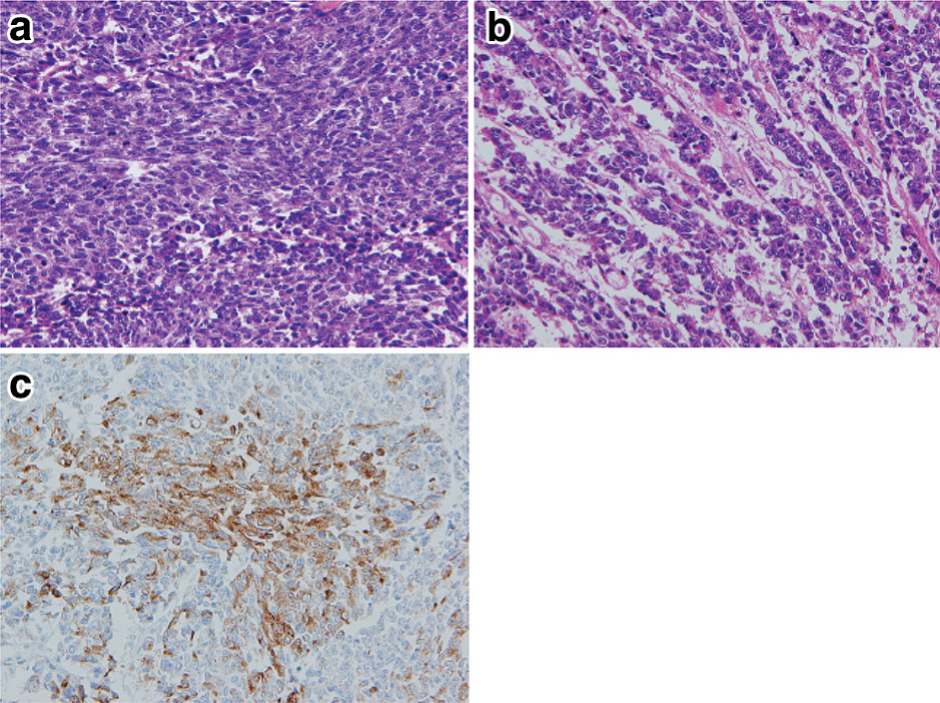
Neuroendocrine carcinoma, small cell type. **a** Small round cells with scant cytoplasm proliferate densely. **b** Tumor cells show an irregular arrangement with trabecular or ribbon-like patterns. **c** Immunohistochemically, the tumor cells are positive for chromogranin A, indicating differentiation to neuroendocrine cells

Neuroendocrine cell tumors include both neuroendocrine tumors (formerly known as carcinoid tumors) and neuroendocrine carcinoma (formerly known as endocrine cell carcinoma). Neuroendocrine tumors of the esophagus are very rare and have the same histological appearance as neuroendocrine tumors located in other organs. Neuroendocrine carcinoma forms nests of tumor cells in various sizes and occasionally shows a trabecular or ribbon-like growth pattern or rosette formation. This tumor can be classified into two types: small cell type and non-small cell type (mixed type of small and large cells). Definitive diagnosis needs immunohistochemical positivity of endocrine markers such as chromogranin A, synaptophysin, and CD56 (N-CAM).

- Note: Tumors exhibiting neuroendocrine differentiation were formerly diagnosed as undifferentiated carcinoma. Neuroendocrine carcinoma can be distinguished from undifferentiated carcinoma by the presence of neuroendocrine differentiation.

9. Undifferentiated carcinoma

This tumor shows medullary growth pattern consisting of small and large tumor cells, lacking particular cell and structural differentiation. Various histopathological methods fail to reveal any particular cell differentiation.

10. Others

This category includes malignant epithelial tumors that cannot be classified in any of the above groups.

4.2.1.4. Non-epithelial tumors

1. Smooth muscle tumor (Fig. 2-39)

**Fig. 2-39:**
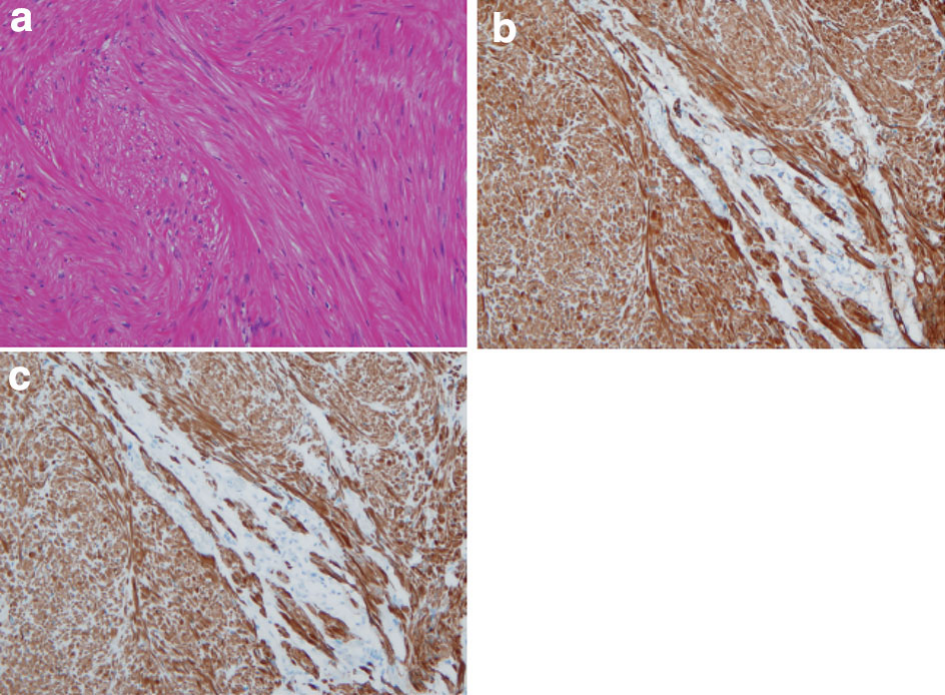
Leiomyoma. **a** Spindle cells with eosinophilic cytoplasm scantily grow in the fascicular pattern. **b** Immunohistochemically, the tumor cells are positive for α-smooth muscle actin. **c** Tumor cells are also positive for desmin

Histologically this tumor shows an interlacing pattern of spindle-shaped tumor cells having elongated spindle nuclei with tapered ends and having cytoplasm with eosinophilic filaments. The epithelioid type shows nest-like growth pattern composed of round-shaped tumor cells with eosinophilic or clear cytoplasm. These tumors are often found as a small nodule in the muscularis propria of the lower esophagus or in the muscularis mucosae or the muscularis propria of the mid-esophagus. This tumor shows positive immunohistochemical reactions for α-smooth muscle actin and desmin and a negative reaction for KIT (CD117).

Differentiation between benign and malignant tumor is based on the cellularity, nuclear pleomorphism, and mitosis. Leiomyoma has no nuclear pleomorphism or mitotic figures and shows low cellularity, whereas leiomyosarcoma has abundant mitotic figures and shows high cellularity.

2. Gastrointestinal stromal tumor (GIST) (Fig. 2-40)

**Fig. 2-40:**
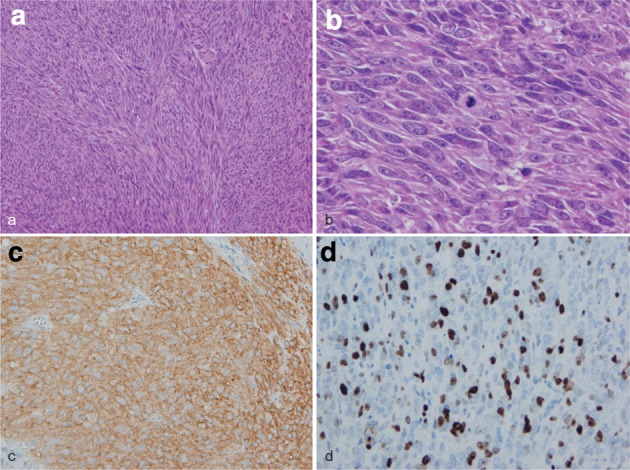
Gastrointestinal stromal tumor (GIST). **a** Spindle cells densely proliferate with fascicular arrangement. **b** Mitotic figures are shown among tumor cells with plump nuclei. **c** Immunohistochemically, the tumor cells are positive for KIT (CD117). **d** The Ki-67 (MIB-1) labeling index was 30%, indicating a high-risk tumor

This tumor shows histologic findings similar to a smooth muscle tumor. It is occasionally difficult to differentiate GIST from smooth muscle tumor without immunohistochemical staining. GIST is defined as a mesenchymal tumor that immunohistochemically exhibits KIT (CD117) and/or DOG1 positivity. CD34 is positive in about 80% of GISTs.

- Note: Risk assessment follows the Guidelines for the Diagnosis and Treatment of GIST.

3. Granular cell tumor (Fig. 2-41)

**Fig. 2-41:**
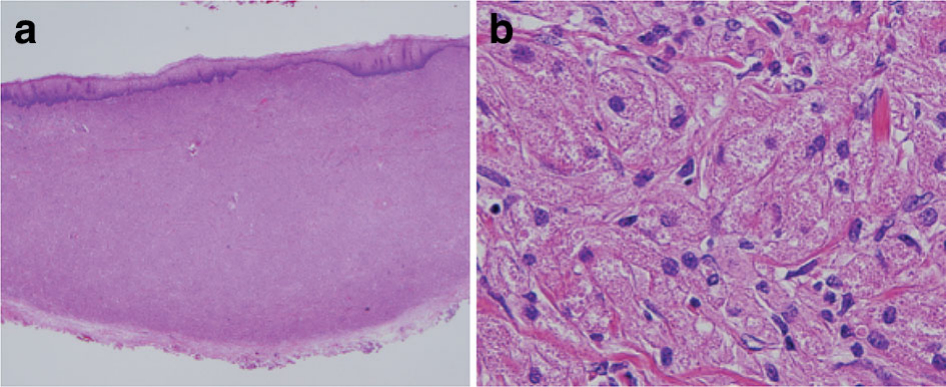
Granular cell tumor. **a** The tumor has grown mainly within the lamina propria mucosae and is covered by stratified squamous epithelium. **b** Large and round tumor cells exhibit abundant granular and eosinophilic cytoplasm

Granular cell tumors, which are composed of large round cells with eosinophilic granules in abundant cytoplasm, form solid nests of various sizes and grow predominantly in the lamina propria mucosae or submucosa. Stratified squamous epithelium commonly covers these tumors, and they occasionally exhibit pseudoepitheliomatous hyperplasia.

4.2.1.5. Lymphoid tumors

There have been a few case reports of B cell lymphoma of the esophagus. The latest version of the WHO classification is used for esophageal malignant lymphoma.

4.2.1.6. Other malignant tumors

1. Malignant melanoma (Fig. 2-42)

**Fig. 2-42:**
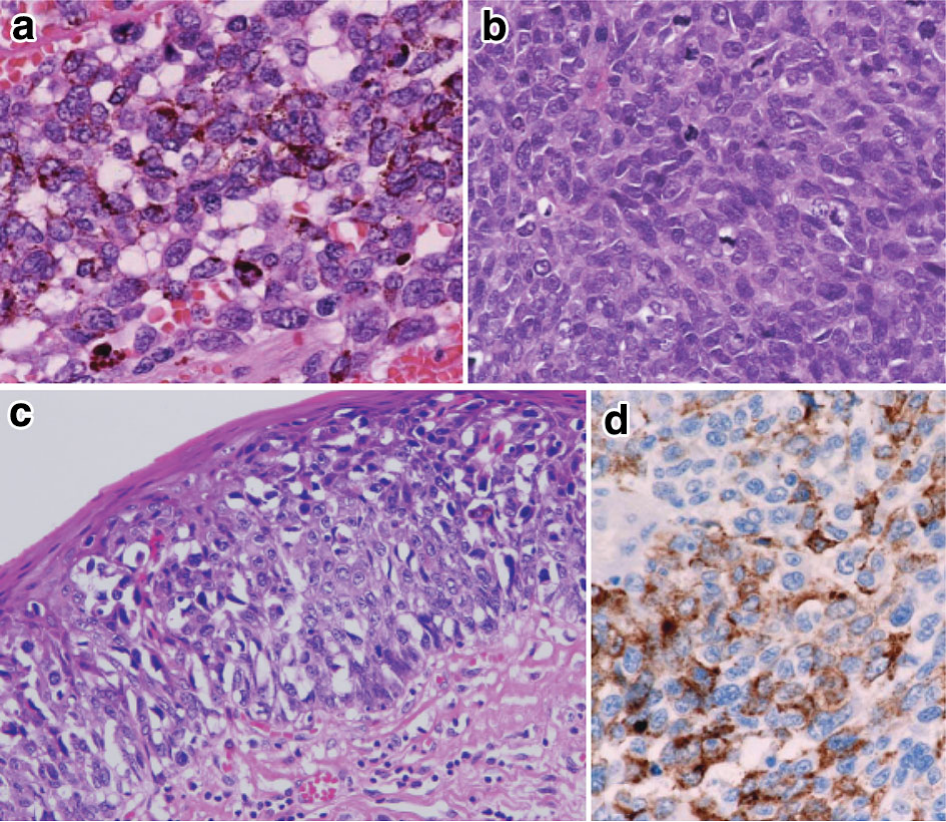
Malignant melanoma. **a** Short spindle-shaped or round tumor cells with abundant melanin granules have proliferated densely. **b** No melanin granules are observed in amelanotic melanoma. **c** Tumor cells with a clear cytoplasm show intraepithelial spread adjoining an invasive tumor. **d** Immunohistochemically, the tumor cells are positive for HMB-45 (Human Melanoma Black-45)

In order to make a diagnosis of primary malignant melanoma, histological identification of junctional activity that tumor cells producing melanin granules proliferate in the basal layer of the epithelium is needed.

2. Others

Choriocarcinoma has been reported in the esophagus.

*4.2.2. Depth of tumor invasion (pT)*

If the layers of the esophagus are destroyed by preoperative treatment, the depth of tumor invasion should be described based on the deepest layer where the primary tumor is considered to have been present before preoperative treatment.

*4.2.9. Pathological criteria for the effects of radiation and/or chemotherapy*

Some of the findings regarded as therapeutic effect due to radiation and chemotherapy are described below (Figs. 2-43, 2-44, 2-45, 2-46).

**Fig. 2-43:**
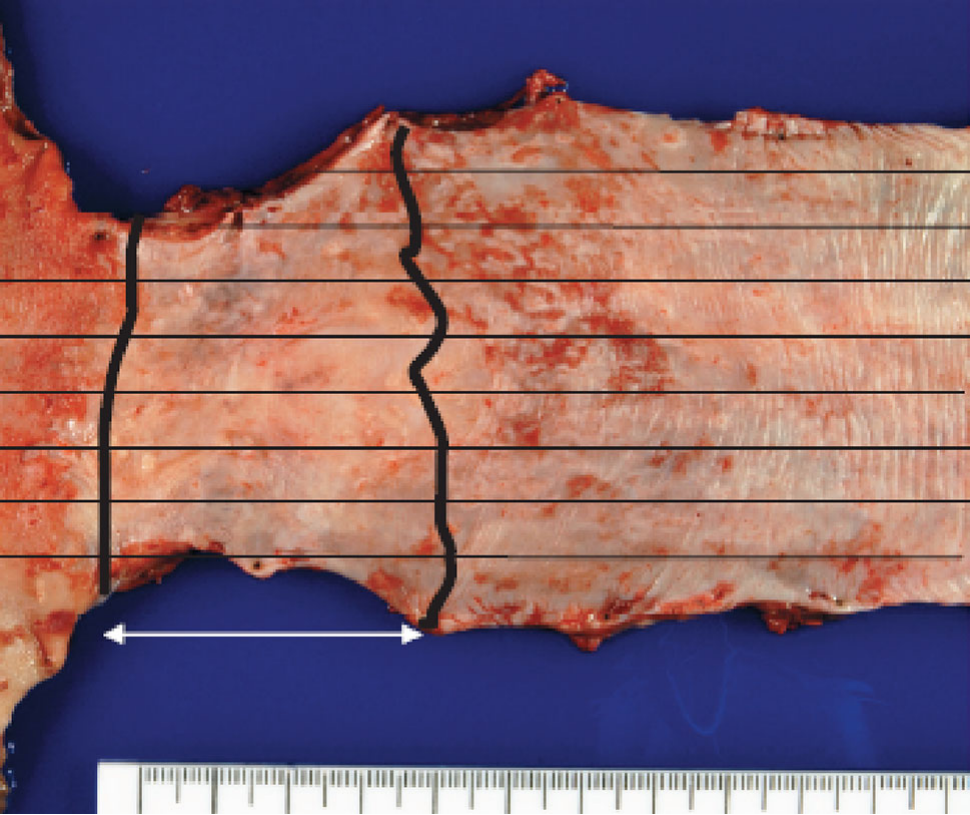
Resected specimen receiving chemoradiotherapy

**Fig. 2-44:**
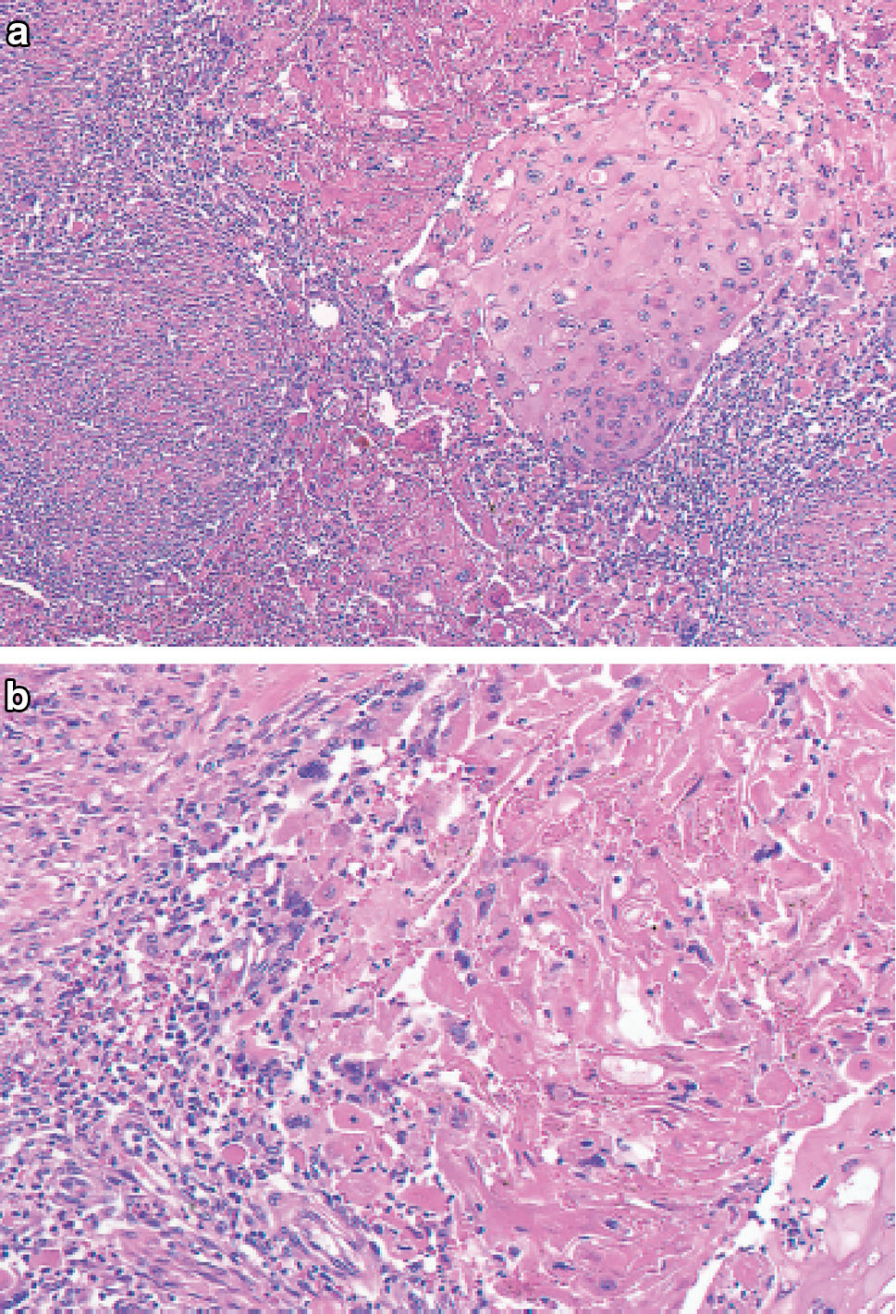
Grade 1: slightly effective. **a ** ×100. **b** ×200 high-magnification view of **a**

**Fig. 2-45:**
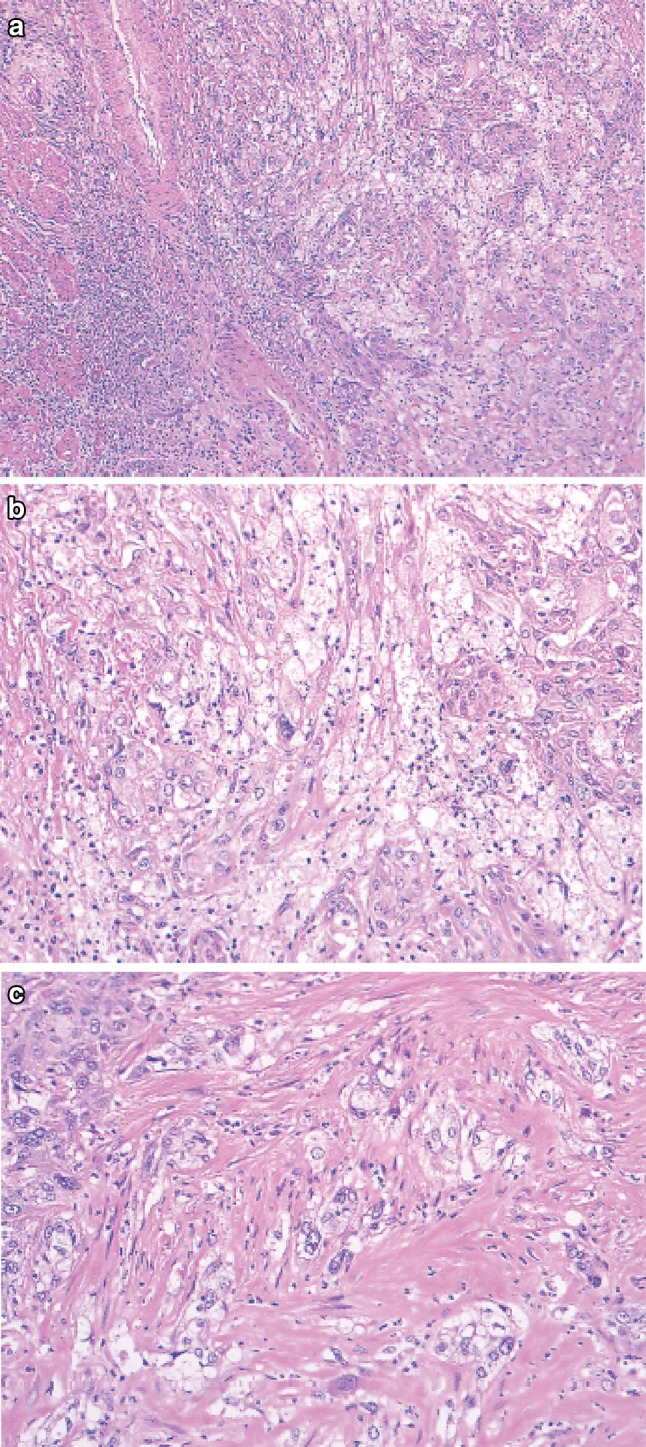
Grade 2: moderately effective. **a** ×100. **b** ×200 High-magnification view of ** a**. **a**, **b** Small nests of a tumor are surrounded by macrophages with foamy cytoplasm. Most of the residual cancer cells show degeneration, and decreased staining with eosin. Tumor nests are surrounded by inflammatory cells. The foamy cells are regarded as a reaction to liquefactive necrosis. **c** Another section of the same case. Scattered tumor cells show vacuolation of the cytoplasm and nuclei, and are surrounded by marked fibrosis

**Fig. 2-46:**
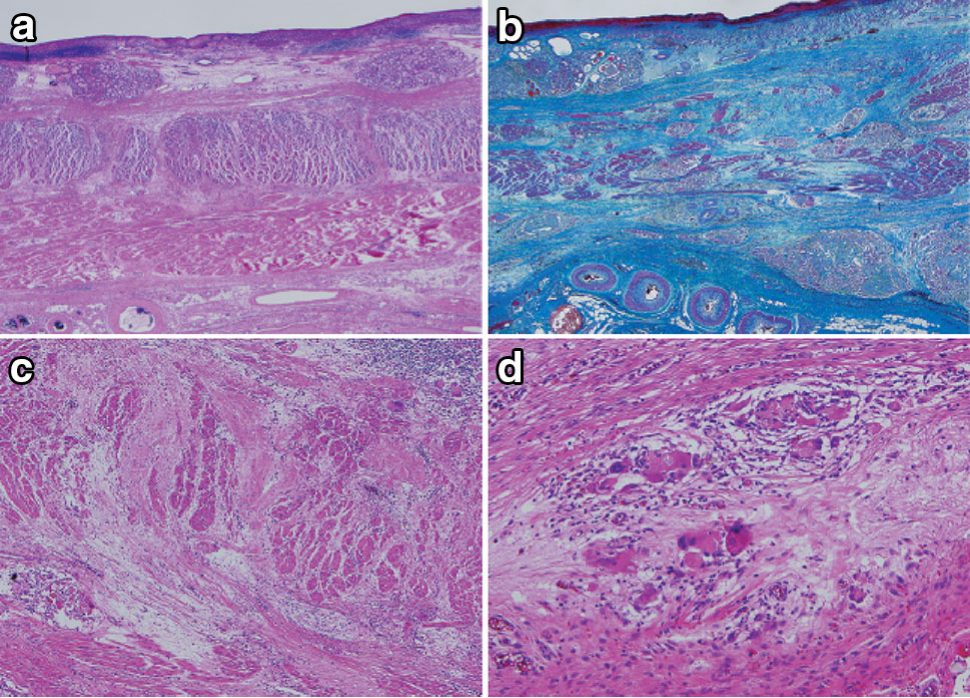
Grade 3: Markedly effective. **a** Marked fibrosis is noted beneath the squamous epithelium. **b** Masson staining reveals partial disruption of muscular layer. Fibrosis is noted throughout the esophageal wall. The extent of fibrosis can be regarded as the extent of the preexisting tumor. **c** Disruption of the muscularis propria and fibrosis are noted. **d** No viable cancer cell is observed, while foreign body giant cells are *scattered*. The therapeutic effect is evaluated as grade 3

Preoperative chemoradiotherapy was performed for a Type 2 advanced cancer. In the resected specimen, only mild stricture and wall thickening (arrow) are observed in the lower esophagus.

Serial sectioning was performed for pathological examinations. Histologically, fibrosis was observed in the stricture, which can be regarded as the extent of the preexisting tumor.

Tumor nests with abundant keratinization have unclear borders, and are surrounded by inflammatory cell infiltration, foreign body giant cells, and granulation tissue with marked fibrosis. Viable tumor cells are observed in the center of the tumor. It is a characteristic finding that keratinized material directly contacts the granulation tissue without a basal layer or prickle cell layer. Numerous viable tumor nests are observed also in other sections.

6. Barrett esophagus and adenocarcinoma in Barrett esophagus

*6.1.3. Barrett esophagus*

6.1.3.1. Macroscopic findings (Fig. 2-47)

**Fig. 2-47:**
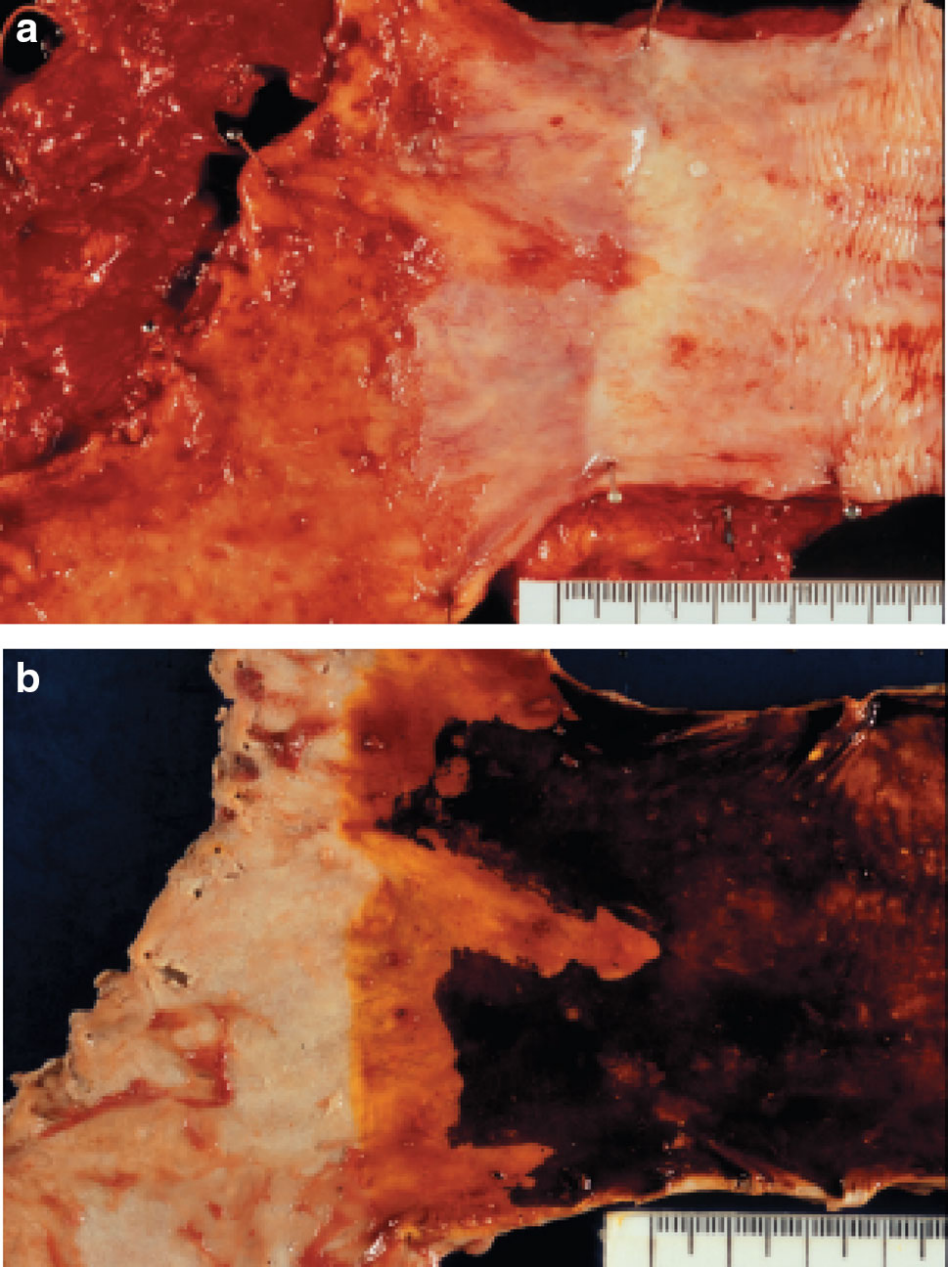
**a** Barrett esophagus and adenocarcinoma in Barrett esophagus, 0-IIb. The squamocolumnar junction shows an irregularity, with a tongue-like extension of the columnar-lined mucosa towards the esophagus. **b** Iodine-stained specimen. Iodine staining clearly shows a tongue-like extension of columnar-lined mucosa measuring 30 mm (short segment Barrett esophagus: SSBE). Cancerous lesion was detected pathologically in Barrett mucosa, but it is not visible macroscopically. 0-IIb, pT1a-SMM

6.1.3.2. Pathological findings

Any of the following findings can be observed in Barrett esophagus.

1. Esophageal glands or ducts beneath the overlying columnar epithelium.

2. Squamous epithelial islands located in the columnar epithelium

3. Double-layer muscularis mucosae beneath the overlying columnar epithelium

- Note: The presence of palisading vessels with a diameter of more than 100 m in the lamina propria mucosae of the lower esophagus suggests Barrett esophagus.

Types of Barrett mucosa

1. Specialized columnar epithelium (SCE)

Foveolar epithelium has goblet cell metaplasia (incomplete intestinal metaplasia). Paneth cells are rarely observed.

2. Junctional type

Cardiac gland-like epithelium sometimes includes parietal cells.

3. Gastric fundic type

The epithelium contains chief and parietal cells.

- Note: If intestinal metaplasia is observed in Barrett mucosa, it should be described

e.g.: Barrett mucosa with SCE (+) (Figs. 2-48, 2-49 and 2-50).

*6.1.4. Adenocarcinoma in Barrett esophagus (Figs.* 2-51, 2-52 *)*

**Fig. 2-48:**
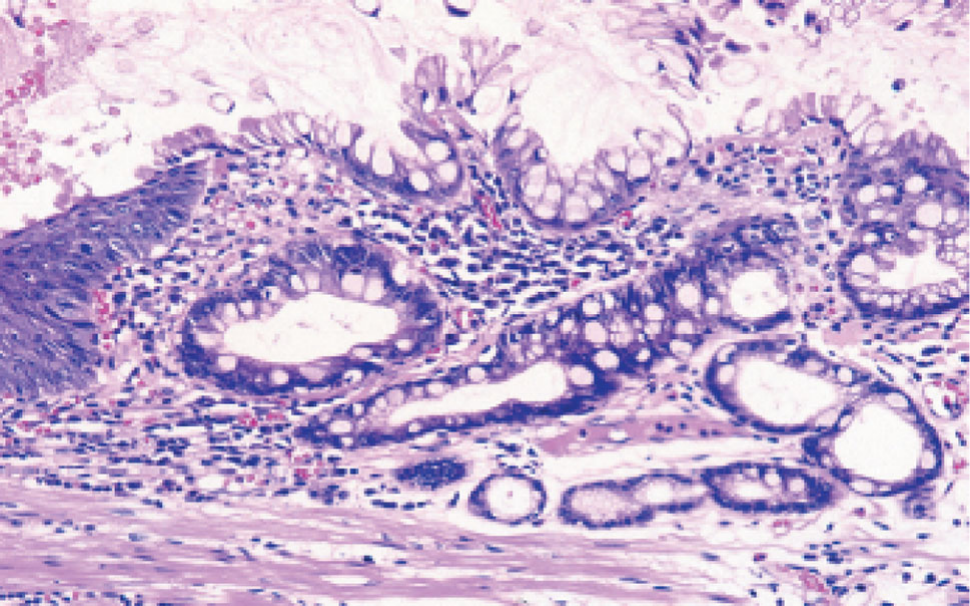
Barrett esophagus (specialized columnar epithelium): the esophagus is covered by specialized columnar epithelium with intestinal metaplasia. Squamous epithelium is visible on the distal side (squamous island)

**Fig. 2-49:**
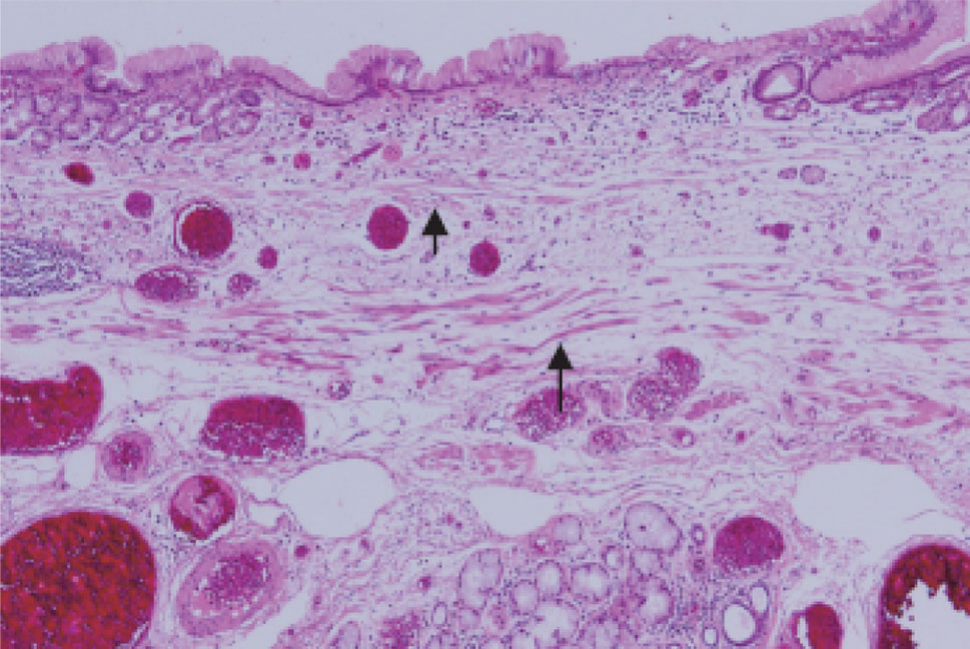
Barrett esophagus (junctional type): a double-layered muscularis mucosae (*arrows*) is present beneath the overlying columnar epithelium. Esophageal glands are also observed in the submucosal layer. Overlying columnar epithelium is of the cardiac gland type

**Fig. 2-50:**
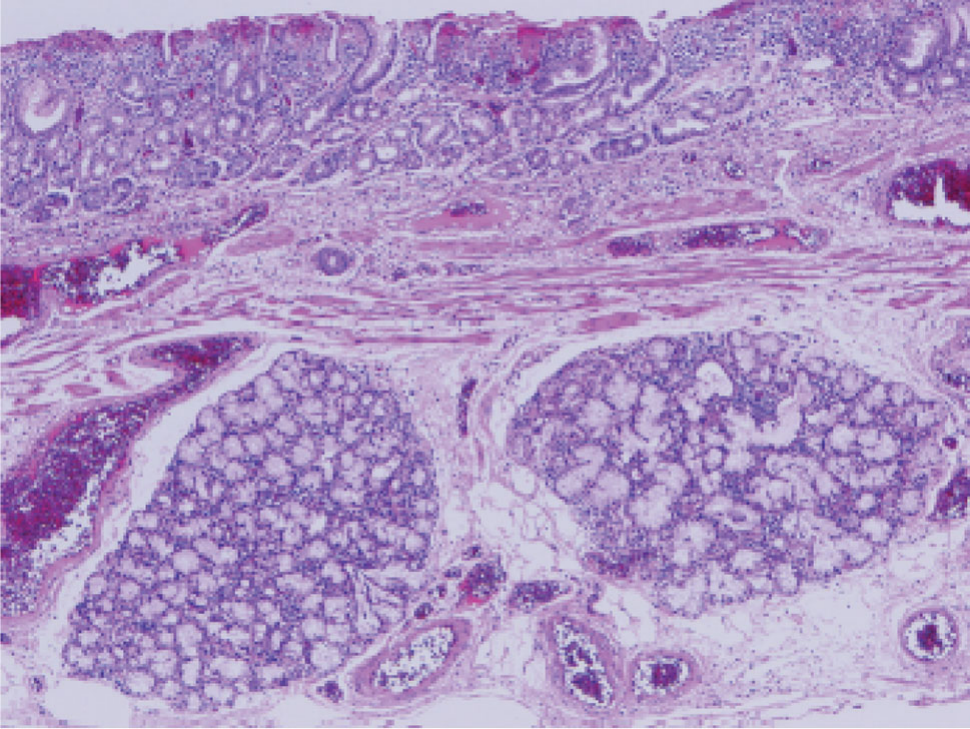
Barrett esophagus (gastric fundic type): the mucosa consists of fundic gland type columnar epithelium. Esophageal glands are observed in the submucosal layer

**Fig. 2-51:**
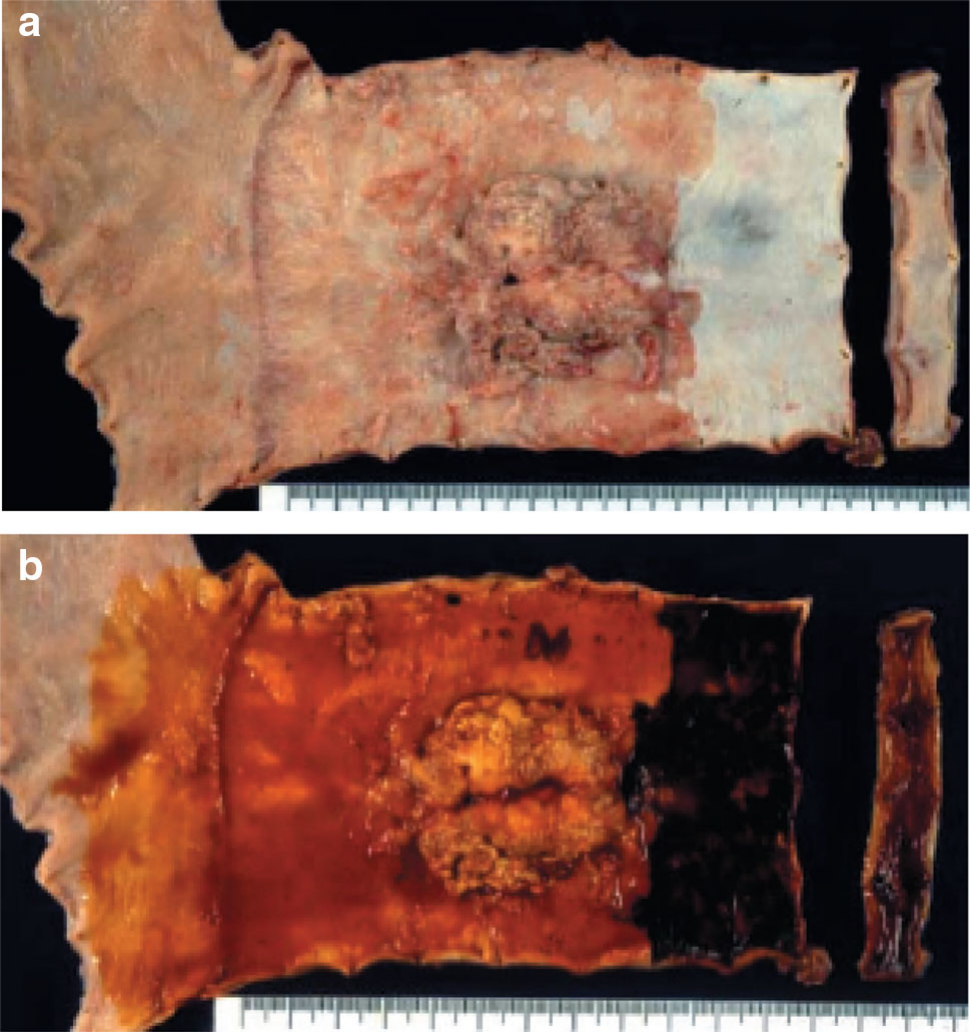
**a** Adenocarcinoma in Barrett esophagus. Grossly type 1. A section of columnar epithelium measuring 85 mm in length and continuously extending to the esophagus is regarded as long segment Barrett esophagus. A protruding tumor (Type 1) is visible within the section of Barrett esophagus. **b** Iodine-stained specimen shown in ** a**. Iodine staining clearly reveals the area of Barrett mucosa with scattered iodine-stained squamous islands. The depth of tumor invasion is pT1b

**Fig. 2-52:**
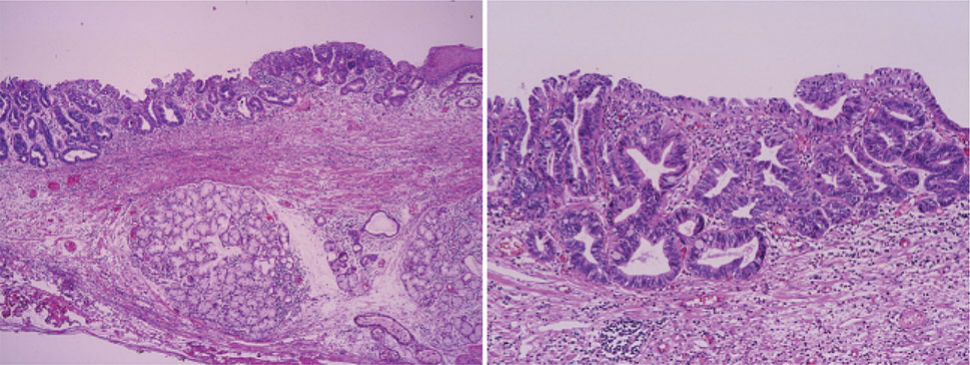
Adenocarcinoma in Barrett esophagus. Barrett esophagus with esophageal glands in the submucosal layer is covered with well differentiated adenocarcinoma. The tumor has invaded beyond the superficial muscularis mucosae, but has not reached the original (*deep*) muscularis mucosae. Therefore, the depth of tumor invasion should be assessed as pT1a-LPM

**Part III**

**Response Evaluation Criteria in Radiotherapy and Chemotherapy for esophageal cancer**

Introduction

The Japan Esophageal Society (JES) had adopted the “Response Evaluation Criteria in Chemotherapy for Solid Tumors” published by the Japan Society of Clinical Oncology (JSCO), which were based on the WHO evaluation criteria, as criteria for response evaluations. According to global changes from the WHO to the “Response Evaluation Criteria in Solid Tumors (RECIST)” criteria, JSCO has already adopted these criteria, which has led JES to the same decision. The response evaluation criteria for the primary site of the esophageal cancer are included in this revised version.

Concerning adapting RECIST into the present criteria, the policy of RECIST is described to ensure its proper use. Both the WHO and RECIST criteria are targeted to evaluate the effect of antitumor agents on tumor shrinkage. Their purposes are to estimate the response ‘as a surrogate end point in clinical trials’. RECIST can be used in daily clinical practice; however, it is not intended for making any decisions on treatment strategy. Achieving a “partial response (PR)” with 50% reduction in tumor size does not mean giving a definite advantage to the patient. “Clinical improvement” may not correspond to “objective tumor response” in daily practice. The RECIST guidelines clearly state that “the defined criteria are not necessarily developed further to be applicable or complete in such a context”. Physicians who are using the present criteria should recognize that the RECIST criteria are only for evaluating antitumor activity as a surrogate end point in clinical trials.

The present criteria added the evaluation criteria for primary tumor of the esophagus. These additional descriptions are needed to define complete response (CR) for the primary tumor, since the CR rate was more correlated with prognosis after definitive chemoradiotherapy or radiotherapy than response rate^2)^. It should be noted that these criteria are indicative not for pathological response but for clinical response as a surrogate end point related to prognosis. These criteria are defined for “evaluating tumor reduction as an end point in clinical trials” and do not attempt to determine the value of esophagography as an evaluation tool in daily clinical practice. Although the value of “PR” is not confirmed, this definition is described in “Notes” since it may be used in cases of preoperative therapy. The development of new imaging modalities, such as positron-emission tomography (PET), may allow the proposal of new criteria in the future.

**1. Subjects**

Subjects evaluated by the present criteria are patients with esophageal cancer and treated with chemotherapy and/or radiotherapy.

1.1. Classification of tumor lesions

*1.1.1. Measurable lesions*

Measurable lesions defined as lesions that can be accurately measured in at least one dimension (greatest dimension to be recorded) as ≥20 mm with conventional techniques or as ≥10 mm with spiral CT scan.

*1.1.2. Non-measurable lesions*

All lesions other than those defined in 1.1.1.

*1.1.3. Target lesions*

Measurable lesions detected at baseline. A maximum of five lesions per organ should be identified as “target lesion”.

*1.1.4. Non-target lesions*

Any other lesions (or sites of disease) should be identified as “non-target lesions”.

**2. Methods for response evaluation**

1. Because it is difficult to accurately measure the primary site of the esophagus, it is defined as a “non-measurable lesion”. Evaluation of reduction in tumor size of the primary site should be done by esophagoscopy (including biopsy, see 5.) in accordance with the definition of “non-target lesion”.
2. Either measurable or non-measurable lesions apart from the primary esophageal lesion should be evaluated according to the RECIST criteria.
3. Basically the same methods as used in baseline evaluation should be adopted.
4. All measurements should be recorded in metric notation.

**3. Response evaluation criteria for target lesions**

Evaluation of the target lesions should be done for up to 5 lesions greatest in dimension per organ and classified according to the following criteria. Rates of decrease or increase in the sum of the greatest dimensions are calculated by the following formula.

Rates of decrease of the greatest dimensions = {(sum of the greatest dimensions at baseline − sum of the greatest dimensions at evaluation)/sum of the greatest dimensions at baseline)} × 100%

Increasing rate of the greatest dimensions = {(sum of the greatest dimensions at evaluation − smallest sum of the greatest dimensions since the treatment started)/smallest sum of the greatest dimensions since the treatment started} × 100%

3.1. Complete response (CR)

The disappearance of all target lesions as well as secondary changes associated with the tumors.

- Note: In case of lymph node metastasis, CR is declared if the size decreases to normal size or less.

3.2. Partial response (PR)

At least a 30% decrease in the sum of the greatest dimensions of target lesions, taking as reference the baseline sum of greatest dimensions.

3.3. Progressive disease (PD)

At least a 20% increase in the sum of greatest dimensions of target lesions, taking as reference the smallest sum of greatest dimensions recorded since the treatment started.

3.4. Stable disease (SD)

Neither PR nor PD.

**4. Response evaluation criteria for non-target lesion**s

4.1. Complete response (CR)

The disappearance of all non-target lesions and normalization of tumor marker level. In addition, the response of the primary lesion must meet criterion 5.1: complete disappearance of primary lesion on endoscopy.

4.2. Incomplete response/stable disease (IR/SD)

The persistence of one or more non-target lesion(s) and/or the maintenance of tumor marker level above the normal limits. In addition, the response of the primary lesion meets criterion 5.2, incomplete response/stable disease of primary lesion by endoscopy.

4.3. Progressive disease (PD)

The appearance of one or more new lesions and/or unequivocal progression in existing non-target lesion(s). In addition, the response of the primary lesion meets criterion 5.3, progressive disease of the primary lesion on endoscopy.

**5. Response evaluation criteria for primary lesion using endoscopy**

5.1. Complete response of primary lesion (primary lesion CR)

When conditions satisfy all of 1 to 4, the response is judged as CR.

1. Disappearance of endoscopic findings suggesting the presence of a tumor
  The findings noted below should be judged as possible tumor lesions and should not be judged as a CR.
    Eroding changes of the mucosa with an irregular surface
    Ulcerative lesions
    Distinctly protruded changes (including protrusions suggestive of a submucosal tumor)
  The findings noted below should not be judged as possible tumor lesions and should be judged as a CR.
    Scars
    Stenosis
    Iodine-unstained areas or poorly stained areas
    Biopsy-negative granulomatous small elevated lesion
2. Negative endoscopic biopsy findings from the area of the primary tumor
3. Entire esophagus can be observed using endoscopy.
4. No endoscopic findings of active esophagitis (e.g., flat erosive findings, white coating) (Figs. 3-1, 3-2, 3-3, 3-4).

**Fig. 3-1:**
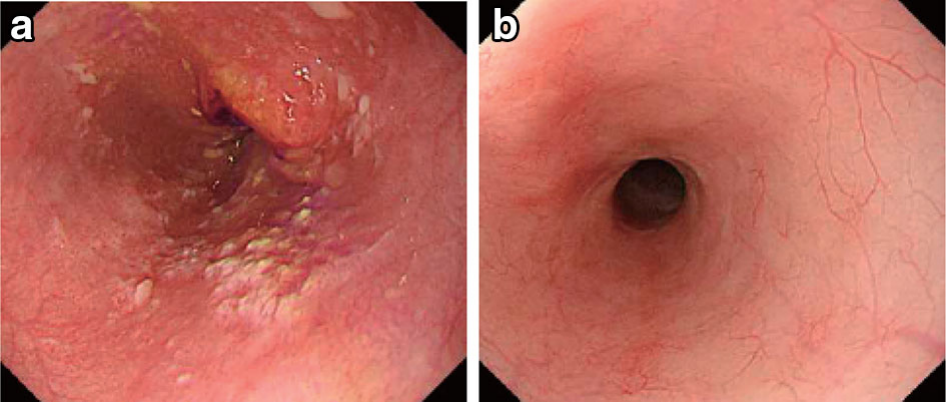
Endoscopic findings of CR cases.  **a** Before treatment: Type 3, cStage IV. **b** After treatment (5 months after CRT): scarring and mild stenosis are visible. The endoscope could be passed through the entire length of the esophagus. This case was judged as a CR

**Fig. 3-2:**
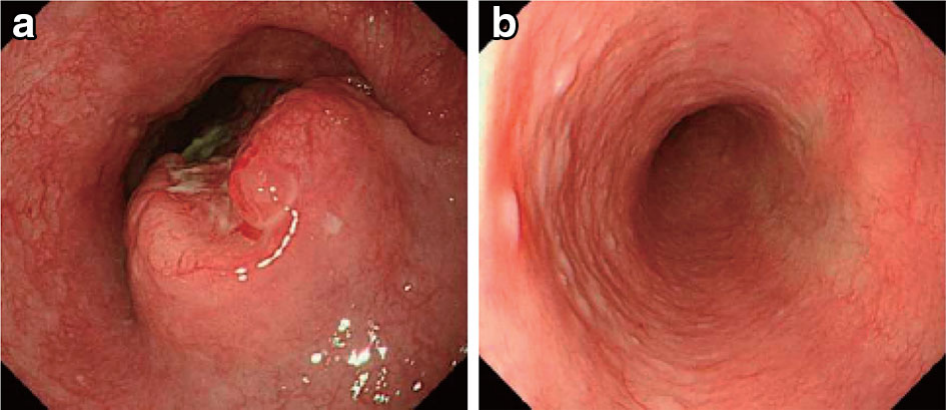
**a** Before treatment: Type 2, cStage III. **b** After treatment (chemotherapy): The tumor has disappeared and only a scar remains. This case was judged as a CR

**Fig. 3-3:**
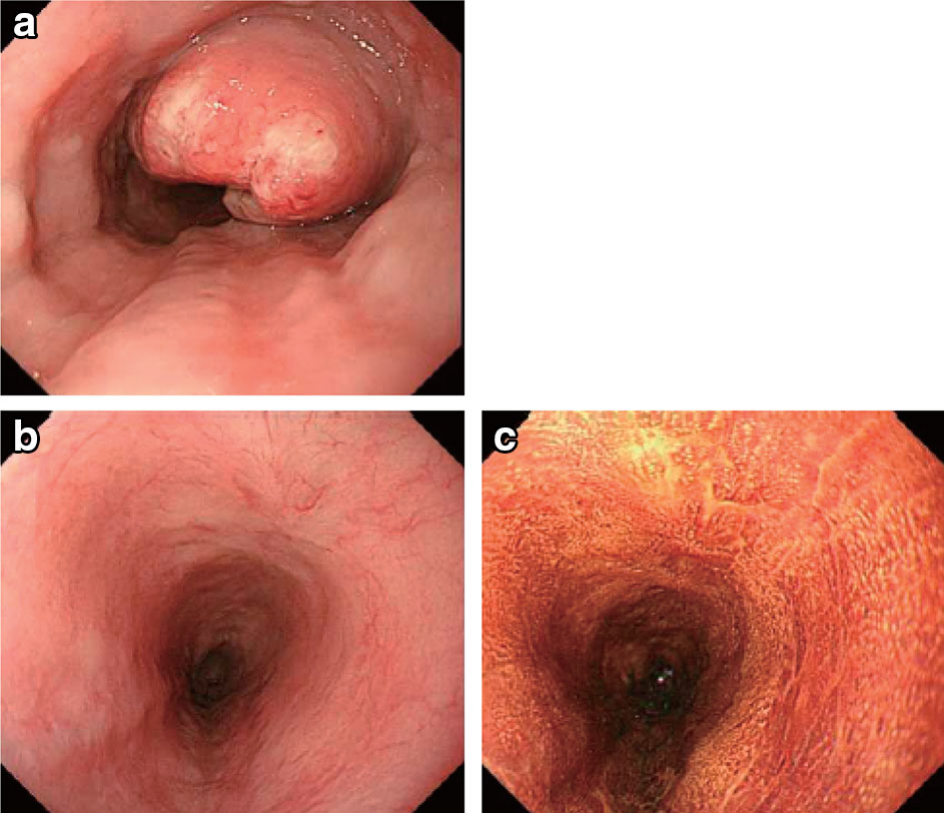
**a** Before treatment: Type 2, cStage III.** b** After treatment (6 months after CRT): the tumor has disappeared, and only a scar remains. **c** After treatment (6 months after CRT): the esophageal mucosa has been stained *brown*. No unstained areas are present. An endoscopic biopsy from the area of the primary tumor was negative

**Fig. 3-4:**
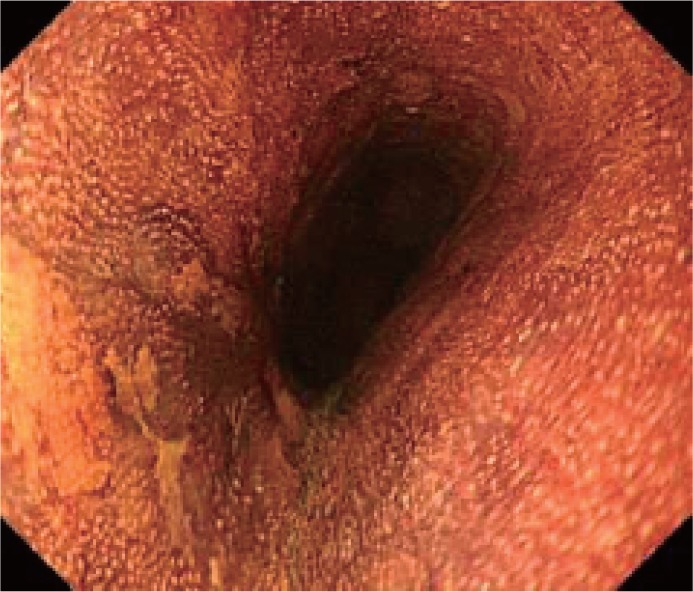
Iodine staining after treatment. The esophageal mucosa was stained unevenly by the iodine. Some areas stained poorly, while others are *dark brown*. An endoscopic biopsy was negative, strongly suggesting a CR

5.2. Incomplete response/stable disease of primary lesion (primary lesion IR/SD)

Response of the primary lesion is judged as IR/SD when its response does not meet the conditions for complete response (5.1) or progressive disease (5.3). PR is not defined in the *Japanese Classification* since endoscopic evaluation for partial response of the primary lesion is difficult. If it is necessary to define PR in cases of preoperative chemotherapy or chemoradiotherapy, refer to the criteria below (Figs. 3-5, 3-6, 3-7, 3-8).

1. Definition of PR in previous version of the guidelines (9th edition)
  Barium esophagography: marked shrinkage in tumor shadow.
  Endoscopy: shrinkage or flattening in the tumor or its surrounding ridge, and shrinkage in ulcerative lesion. Marked morphological improvement.
2. Adapt criteria of partial response defined in 3.2 for primary lesions more than 20 mm in greatest dimension by helical CT scan.

**Fig. 3-5:**
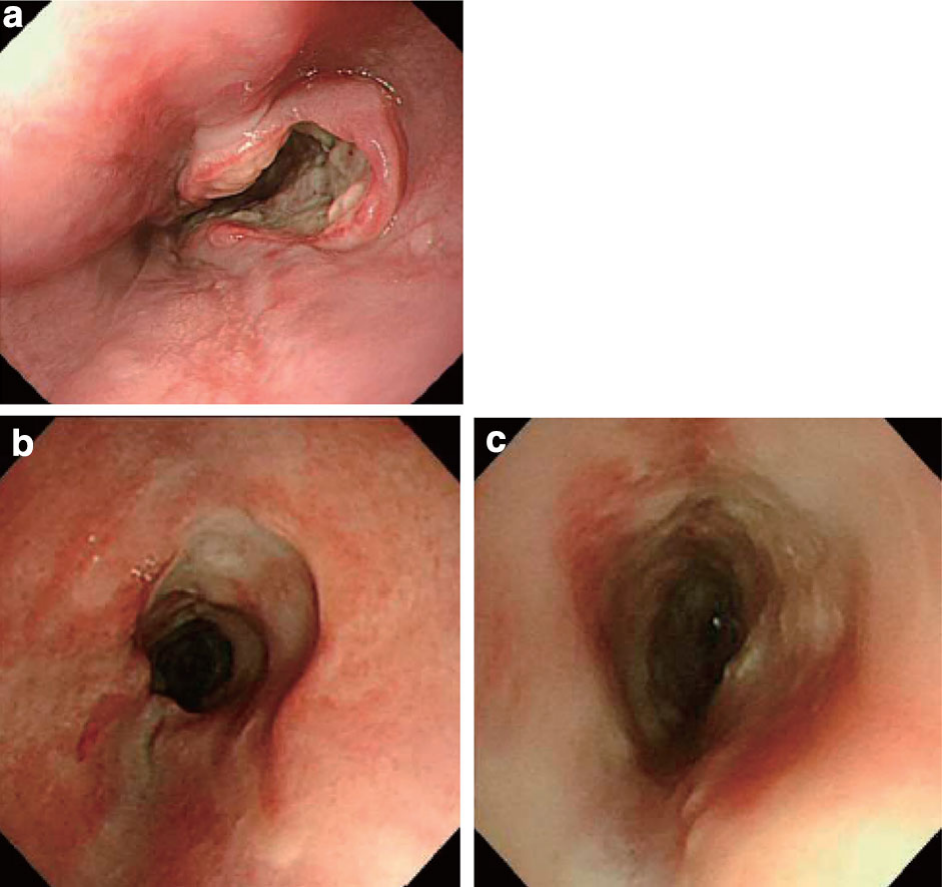
Endoscopic findings of IR/SD cases.  **a** Before treatment: Type 2, cStage III. **b**, **c** After treatment (1 month after CRT): an ulcer remains

**Fig. 3-6:**
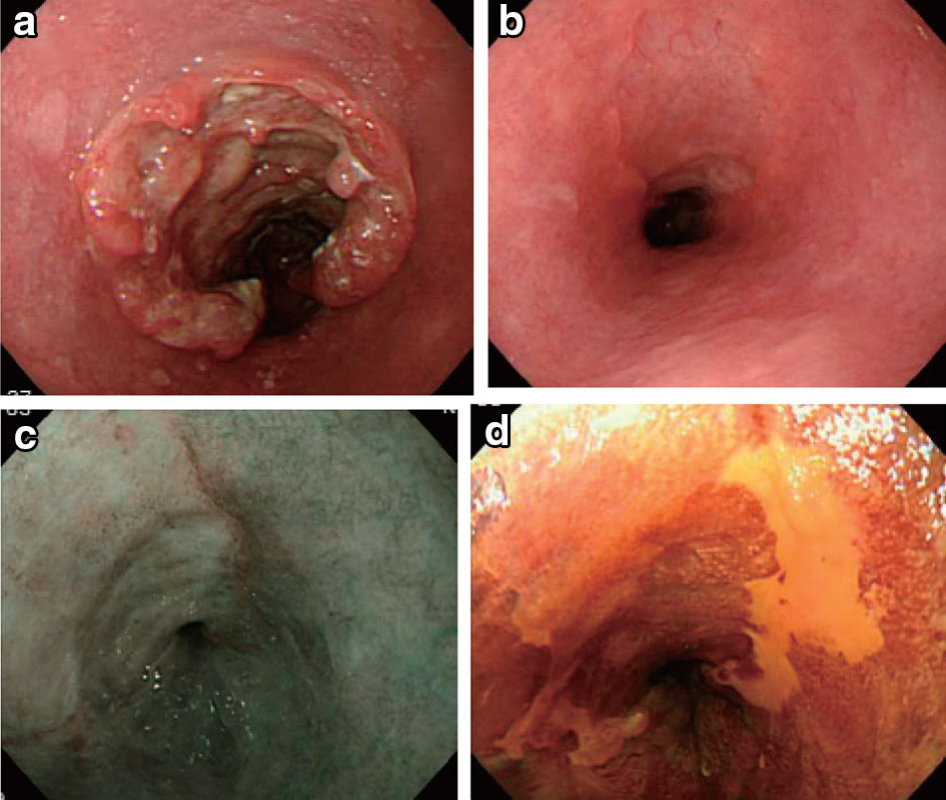
**a** Before treatment: Type 2, cStage IVa. **b** After treatment (CRT): the primary tumor has disappeared. A slightly depressed change surrounded by a marginal elevation is still present. **c** Narrow band imaging after treatment (CRT) shows a* brownish* area at the anal margin. **d** Iodine staining after treatment (CRT): the* brownish* area was identified as an unstained area. An endoscopic biopsy was positive for malignant cells

**Fig. 3-7:**
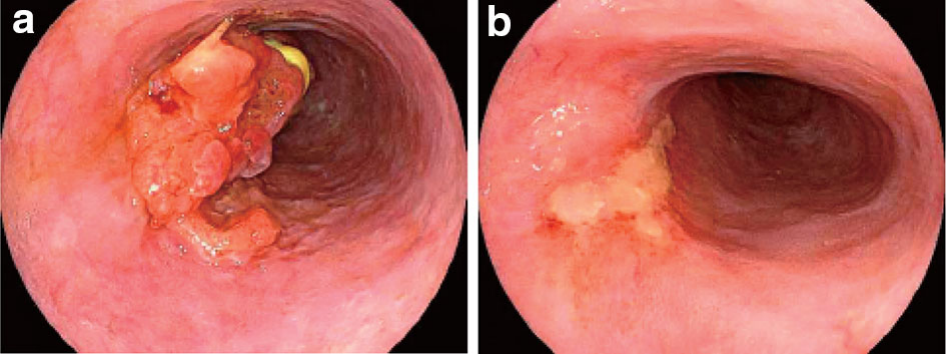
Endoscopic findings of PR cases. **a** Before treatment: Type 1 + 0-IIc cStage III. **b** After treatment (2 months after CRT): a marked tumor reduction is visible, although an ulcer with a marginal elevation is present

**Fig. 3-8:**
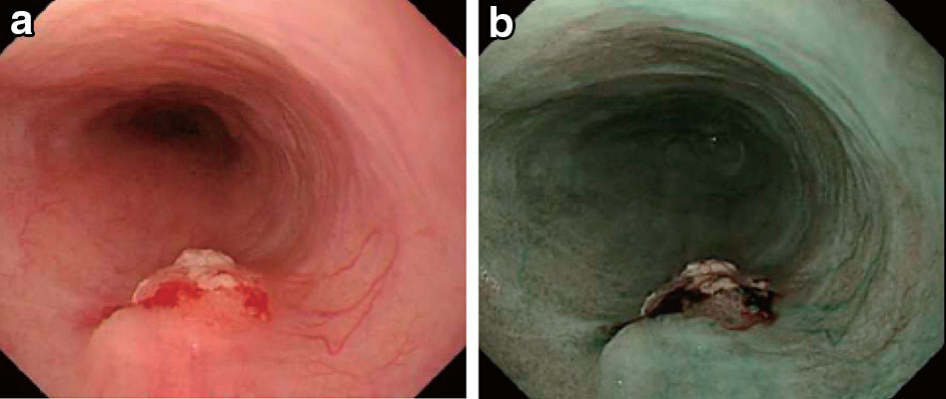
**a** After treatment (3 months after CRT): a residual tumor was strongly suggested by the presence of a submucosal tumor-like protrusion. Cancer tissue is probably exposed on the top and distal half of the tumor. **b** The exposed tumor was identified as a* brownish* area with a central* white* coating using narrow band imaging

[Reference]

Japanese Society for Esophageal Diseases. Guidelines for the Clinical and Pathological Studies on Carcinoma of the Esophagus (in Japanese). 9th ed. Kanehara Shuppan, Tokyo, 1999; 59–79.

5.3. Progressive disease of primary lesion (primary lesion PD)

Distinct tumor growth or progression in esophageal stenosis compared with the best condition during treatment (Fig. 3-9).

**Fig. 3-9:**
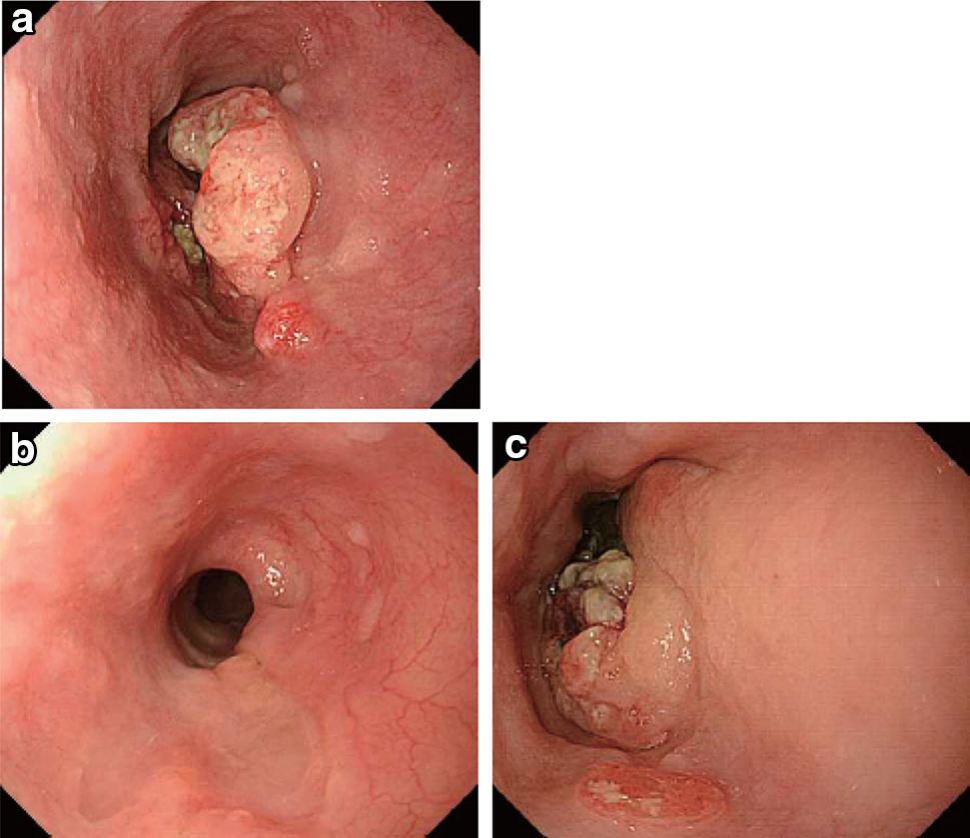
Endoscopic findings of a PD case. **a** Before treatment: Type 2, cStage III. **b** After treatment (2 months after CRT): the primary tumor has mostly disappeared. **c** After treatment (5 months after CRT): the relapsed tumor has increased in size

**6. Overall response**

Overall responses for all possible combinations of tumor responses in target and non-target lesions with or without the appearance of new lesions.

**Table:** 

Target lesions	Non-target lesions	New lesions	Overall response
CR	CR	No	CR
CR	Incomplete response/SD	No	PR
PR	Non-PD	No	PR
SD	Non-PD	No	SD
PD	Any	Yes or no	PD
Any	PD	Yes or no	PD
Any	Any	Yes	PD

If there is no target lesion and only non-target lesions are available (e.g. primary esophageal cancer without lymph node metastasis), overall response is determined only by the responses of the non-target lesions.

**7. Best overall response and confirmation**

7.1. Complete response (CR)

Overall response of CR in serial assessments at an interval of 4 weeks or more.

7.2. Partial response (PR)

Overall response of PR or better response (CR or PR) in serial assessments at an interval of 4 weeks or more.

Endoscopic findings for IR/SD cases

7.3. Stable disease (SD)

When one of the following criteria is met.

1. Best overall response CR or PR was obtained, but duration was less than 4 weeks.

2. Stable disease or better response in serial assessments at an interval of 4 weeks or more, at 4 weeks or more after the start of treatment. (If the first assessment is SD, and the second assessment is of PD, it should be judged as PD.)

- Note: If there is no target lesion (e.g. esophageal cancer without metastasis), IR/SD is used as a description in place of PD or SD.

7.4. Progressive disease (PD)

When none of above conditions is met.

- Note: In phase II studies in which response rates are used as end points, confirmation should be made with an interval of 4 weeks or more. In phase III, studies in which survival is used as the end point, the best overall response without confirming response can be used.

